# CRISPR editing of *TERMINAL FLOWER 1* recapitulates perpetual flowering in strawberry, mimicking repression by the PERPETUAL FLOWERING locus

**DOI:** 10.1093/plphys/kiag684

**Published:** 2026-10-09

**Authors:** Marta Bjornson, Michael A Hardigan, Juan M Debernardi, Hillel Brukental, Dominique D A Pincot, Mishi V Vachev, Randi A Famula, Glenn S Cole, Dianne Velasco, Maher Al Rwahnih, Mitchell J Feldmann, Steven J Knapp

**Affiliations:** Department of Plant Sciences, University of California Davis, One Shields Avenue, Davis, CA 95616, United States; Department of Plant Sciences, University of California Davis, One Shields Avenue, Davis, CA 95616, United States; U.S. Department of Agriculture, Agricultural Research Service, Horticultural Crops Production and Genetic Improvement Research Unit, 3420 NW Orchard Avenue, Corvallis, OR 97330, United States; Department of Plant Sciences, University of California Davis, One Shields Avenue, Davis, CA 95616, United States; Department of Plant Sciences, University of California Davis, One Shields Avenue, Davis, CA 95616, United States; Department of Fruit Tree Sciences, Newe Ya’ar Research Center, Agricultural Research Organization (ARO), Volcani Center, Derech HaMaccabim 68, Rishon LeZion, Israel; Department of Plant Sciences, University of California Davis, One Shields Avenue, Davis, CA 95616, United States; Department of Plant Sciences, University of California Davis, One Shields Avenue, Davis, CA 95616, United States; John Innes Centre, Norwich Research Park, Colney Ln, Norwich NR4 7UH, United Kingdom; Department of Plant Sciences, University of California Davis, One Shields Avenue, Davis, CA 95616, United States; Department of Plant Sciences, University of California Davis, One Shields Avenue, Davis, CA 95616, United States; Foundation Plant Services, University of California Davis, One Shields Ave, Davis, CA 95616, United States; Foundation Plant Services, University of California Davis, One Shields Ave, Davis, CA 95616, United States; Department of Plant Sciences, University of California Davis, One Shields Avenue, Davis, CA 95616, United States; Department of Plant Sciences, University of California Davis, One Shields Avenue, Davis, CA 95616, United States

## Abstract

The development of perpetual flowering (PF) cultivars revolutionized octoploid strawberry (*Fragaria*  *×*  *ananassa*) cultivation by enabling nearly year-round fruit production and doubling fruit yields compared to historic seasonal flowering (SF) cultivars, which flower in spring. Most commercial PF cultivars result from the introgression of a dominant *PERPETUAL FLOWERING* (*PF*) allele at the PERPETUAL FLOWERING RUNNERING (PFRU) locus. PFRU has been genetically and physically mapped to chromosome 4B, but the causal gene(s) remain unknown. Here we used the haplotype-phased “UCD Royal Royce” (*PFpf*) genome, next-generation sequencing–based *k*-mer genome-wide association study (*k*-mer GWAS), and RNAseq to investigate PFRU. We narrowed the locus down to a precise physical address and revealed abundant genetic and transcriptomic differences in the locus and associated with flowering. Outside the PFRU locus, flowering regulation diverges at expression of *TERMINAL FLOWER 1* (*TFL1*) in all 4 subgenomes, analogous to diploid *Fragaria vesca*. This critical step of the flowering pathway is altered by *PF* in diverse cultivars, and gene editing knockout of *TFL1* is sufficient to induce flowering under long days in a seasonal flowering cultivar. Together, these results shed light on the mechanism of flowering regulation by the PF locus and expand biotechnological approaches to achieve extended flowering in any strawberry cultivar.

## Introduction

Strawberry (*Fragaria* × *ananassa*) is cultivated across a wide range of temperate, subtropical, and protected production systems on every inhabited continent. Global strawberry production now exceeds 9 million metric tons annually, with major contributions from Asia, Europe, Africa, and the Americas, reflecting the crop's adaptability to diverse climates and management regimes (FAO 2021). Over the past half-century, the expansion and geographic diversification of strawberry production has been driven in part by genetic innovations, including the discovery and deployment of the PERPETUAL FLOWERING/RUNNERING (PF/PFRU) locus, originally introgressed via phenotypic selection from wild germplasm and subsequently incorporated into cultivated backgrounds (Feldmann et al 2024). Seasonal flowering (SF) strawberry cultivars determine floral meristems in the cool short days of fall and winter, produce fruit from these meristems in the next year, and stop flowering as day length and temperature increase (Gaston et al 2021), with the precise timing dependent on environmental factors. In contrast, PF cultivars continue flowering through summer, into fall, and even into winter, depending on the planting date (Knapp et al 2023). Thus, the deployment of PF-carrying cultivars enabled extended, near-year-round fruit production, facilitating the spread of strawberry cultivation into new regions and production systems, including protected and off-season summer culture (Holmes 2024). These advances not only transformed regional production calendars but also reshaped global strawberry supply chains, laying the foundation for the modern, continuously producing strawberry industry (Menzel 2025).

Perpetual flowering has been a trait of interest in strawberry cultivation almost as long as the octoploid cultivated strawberry has existed, and several presumably independent sources of PF have been recorded. In 1866, probably less than 100 years after the hybridization that gave rise to *F.* × *ananassa*, the cultivar “Gloede's seedling” was introduced as a remontant octoploid strawberry in Europe (Stewart and Folta 2010). Not long after, Iowa State University breeder Harlow Rockhill released several everbearing accessions with the source that later came to be known as the “Rockhill” source, which dominated American and Canadian everbearing strawberry production in the mid-1900s (Darrow 1966). In the 1950s, the University of California (UC) strawberry breeder Royce Bringhurst collected a day-neutral accession of *Fragaria virginia* ssp. *glauca* in the Wasatch mountains of Utah. Through introgression into UC Davis breeding material, this “Wasatch” source became the widespread economically dominant donor of day neutrality in modern cultivation. “Wasatch”-source perpetual flowering segregates as a single Mendelian trait, with perpetual flowering conferred by the dominant allele (Ahmadi et al 1990). Historically, the trait has been called remontancy, everbearing, or day neutrality; these may, in fact, refer to different sources and expressions of flowering regulation, but pedigree and comparable phenotyping records to clearly delineate these are lacking. Here we use flowering habit to refer to the trait, PF vs. SF as the phenotypic classes, and *PF* (vs. *pf*) to denote the dominant perpetual flowering allele.

Considerable work has gone into understanding the nature of daylength and temperature regulation of flowering in perpetual flowering and seasonal flowering accessions (Durner et al 1984; Sonsteby and Heide 2007; Stewart and Folta 2010; Hytönen and Kurokura 2020; Darby and Islam 2025). In brief, at low temperatures (≲ 12 °C) most strawberries will flower regardless of daylength. Between ∼ 12 and 20 °C, SF plants will flower only under short days, while PF plants will flower under long or short days, and ≳ 25 °C, PF plants require long days for flowering. Thus, between floral induction in fall and the typical growing temperatures on the California coast, PF plants can produce flowers nearly year-round in commercial culture (Holmes 2024).

To understand the biology of octoploid cultivated strawberry, a valuable tool is the diploid wild relative, *Fragaria vesca*. Much like *F.* × *ananassa*, most *F. vesca* accessions require short-day conditions to induce flowering, but some produce floral meristems under long days. Unlike *F.* × *ananassa*, in *F. vesca* this trait is recessive and has been molecularly characterized; *F. vesca* perpetual flowering is conferred by mutation in the *Terminal Flower 1* (*FvTFL1*) gene (Iwata et al 2012; Koskela et al 2012). More broadly, in *F. vesca* long days induce the expression of *Flowering Locus T 1* (*FvFT1*, 1 of 3 *FT* homologs in *F. vesca*; Koskela et al 2012). FvFT1 then induces *Suppressor of Constans 1* (*FvSOC1*), which in turn induces *FvTFL1* and represses flowering (Mouhu et al 2013). In the absence of *FvTFL1*, FvFT1 can induce flowering under long days. Under short days, flowering is induced by FvFT2, although this gene has extremely low expression in critical organs (Gaston et al 2021).

In *F.* × *ananassa*, control of perpetual flowering (or perpetual flowering and runnering) has been mapped many times to a single quantitative trait locus (QTL): PF/PFRU (Gaston et al 2013; Castro et al 2015; Perrotte et al 2016a; Saiga et al 2023; Brukental et al 2025), although some studies suggest other loci can affect expression or intensity of flowering under long days (Perrotte et al 2016b; Lewers et al 2019; Cockerton et al 2023). In the haplotype-phased “FaRR1” genome of the cultivar “UCD Royal Royce” (“Royal Royce”), this locus is located on chromosome 4B. “Royal Royce” is heterozygous *PFpf* and thus this genome contains both SF (FaRR1-SF) and PF (FaRR1-PF) sequences (Hardigan et al 2021) for comparison to determine PF variants. Beyond these 2 haplotypes, more than 100 octoploid strawberry accessions with known flowering habit have now been sequenced (Hardigan et al 2020; Fan and Whitaker 2024). The 4B location of the PF locus may suggest that the mechanism of PF in *F.* × *ananassa* differs from *F. vesca*, as homologs of *TFL1* are found on chromosomes 6A to 6D. Moreover, *TFL1* expression was observed in flowering PF plants (Muñoz-Avila et al 2022), and it has been speculated that in octoploid strawberry PF cultivars, an independent pathway allows flowering under long days (Nakajima et al 2014; Hytönen and Kurokura 2020). Expression of other flowering pathway genes appeared similar in *F.* × *ananassa* and *F. vesca*, with the lowest *TFL1* expression under short days and low temperatures in an SF cultivar (Nakano et al 2015).

Although characterization of the PF locus has not, to date, uncovered the mechanism of flowering control, DNA markers have been developed to predict the trait for plant breeding and can achieve high prediction accuracy. However, several heirloom strawberry cultivars with desirable fruit and disease resistance traits (eg “Earliglow”) are misclassified when using these DNA markers (Salinas et al 2017; Brukental et al 2025). An alternate approach to control flowering in octoploid strawberry is to avoid the PF locus and focus instead on engineering of the flowering pathway. Indeed, silencing of *TFL1* in *F.* × *ananassa* was able to generate a new, synthetic PF trait in the SF cultivar “Elsanta” (Koskela et al 2016). CRISPR-Cas9 gene editing has the potential to update this result by generating new variation affecting strawberry flowering, improving both biological understanding and breeding accuracy for this critical trait.

Here, we use *k*-mer genome-wide association study (GWAS) of 137 sequenced *F.* × *ananassa* accessions to find PF-associated genomic loci: *k*-mer GWAS agrees with single nucleotide polymorphism (SNP)-based mapping approaches and improves precision for a candidate region. By analyzing annotation and expression differences, we identify numerous variants that may cause perpetual flowering in *F.* × *ananassa*. We further find that *TFL1* is repressed throughout the day in PF but not SF meristematic tissue at floral induction under long days, and further that this *TFL1* expression difference at floral induction is conserved across multiple sources of PF and in accessions where existing DNA markers fail. Finally, we show that editing of *TFL1* is sufficient to induce perpetual flowering in *pfpf* strawberry.

## Results

### *k*-mer GWAS precisely delineates the boundaries of the “Wasatch” introgression harboring the *PF* locus

To refine our understanding of the PF locus, we began by conducting *k*-mer GWAS on a population of *n* = 137 individuals published or freshly sequenced for this work (Hardigan et al 2020; Table S1) and aligning *k*-mers to the FaRR1 genome. In *k*-mer GWAS, nucleotide sequences of length *k* are extracted from whole-genome sequencing reads and the presence/absence of each *k*-mer is associated with traits of interest, providing a reference-free approach that can be associated to any genome, identify insertion/deletion (indel) events, and directly identify causative variants (Voichek and Weigel 2020; Gaurav et al 2022; Karikari et al 2023; Cavalet-Giorsa et al 2024). *k*-mer GWAS has most commonly been published using *k*-mers of length 31 (Voichek and Weigel 2020); however, we found that, at this length, there was significant noise across the 4 subgenomes of strawberry, even after filtering for high mapping quality. We extended *k*-mer length to 51 (Gaurav et al 2022; Cavalet-Giorsa et al 2024) and still found some cross-homoeolog contamination, which was largely eliminated when analyzing 101-mers (Figure S1). We thus continued our analysis using 101-mers.

To assess the usefulness of *k*-mer GWAS, we compared *k*-mer results with SNP-based GWAS. We used a population of *n* = 833 accessions phenotyped for PF and genotyped using the FanaSNP Axiom 50 k SNP array (Table S2, Hardigan et al 2020). *k*-mer GWAS identified the same PF-associated peak region on chromosome 4B as SNP-GWAS, albeit with lower statistical certainty as expected given the decreased population size and increased number of observations (Fig. 1a). *k*-mer GWAS also identified peaks on chromosomes 3A and 4A, each with ∼25 *k*-mers passing a *P* < 0.01 threshold. Because many more *k*-mers (>9,000) pass this threshold on chromosome 4B and that peak is also supported by SNP-based GWAS, we focused our attention on this region as the PF locus. We then assessed *k*-mer GWAS as compared to SNP-GWAS at the PF locus (approx. 26 to 29 Mbp on chromosome 4B) and again found similar results. Notably, both in this study and previous mapping with the 50-k and 850-k Axiom SNP arrays, we observed “stair-steps” of association, possibly due to stretches of low recombination interspersed with recombination hotspots (Brukental et al 2025). The extreme density of *k*-mer loci provided a greater resolution for the peak “step” of the PF region and recombination around it despite the considerably smaller population (Fig. 1b). Finally, we mapped PF-associated *k*-mers to FaRR1 haplotype 2—FaRR1-PF. This revealed a homologous region between the 2 haplotypes but did not improve the resolution of the peak region, which likely remains limited by recombination events within the population (Brukental et al 2025, Fig. 1c and 1d). Using these results and a −log_10_(adjusted *P* value) = 5 cutoff, we were able to narrow the segment mostly likely to harbor PF down to region of ∼1 Mbp: FaRR1-PF 26,714,680 to 27,565,700 Mbp and FaRR1-SF 26,491,180 to 27,288,205 Mbp (Fig. 1b and 1d, Table S3).

**Figure 1:**
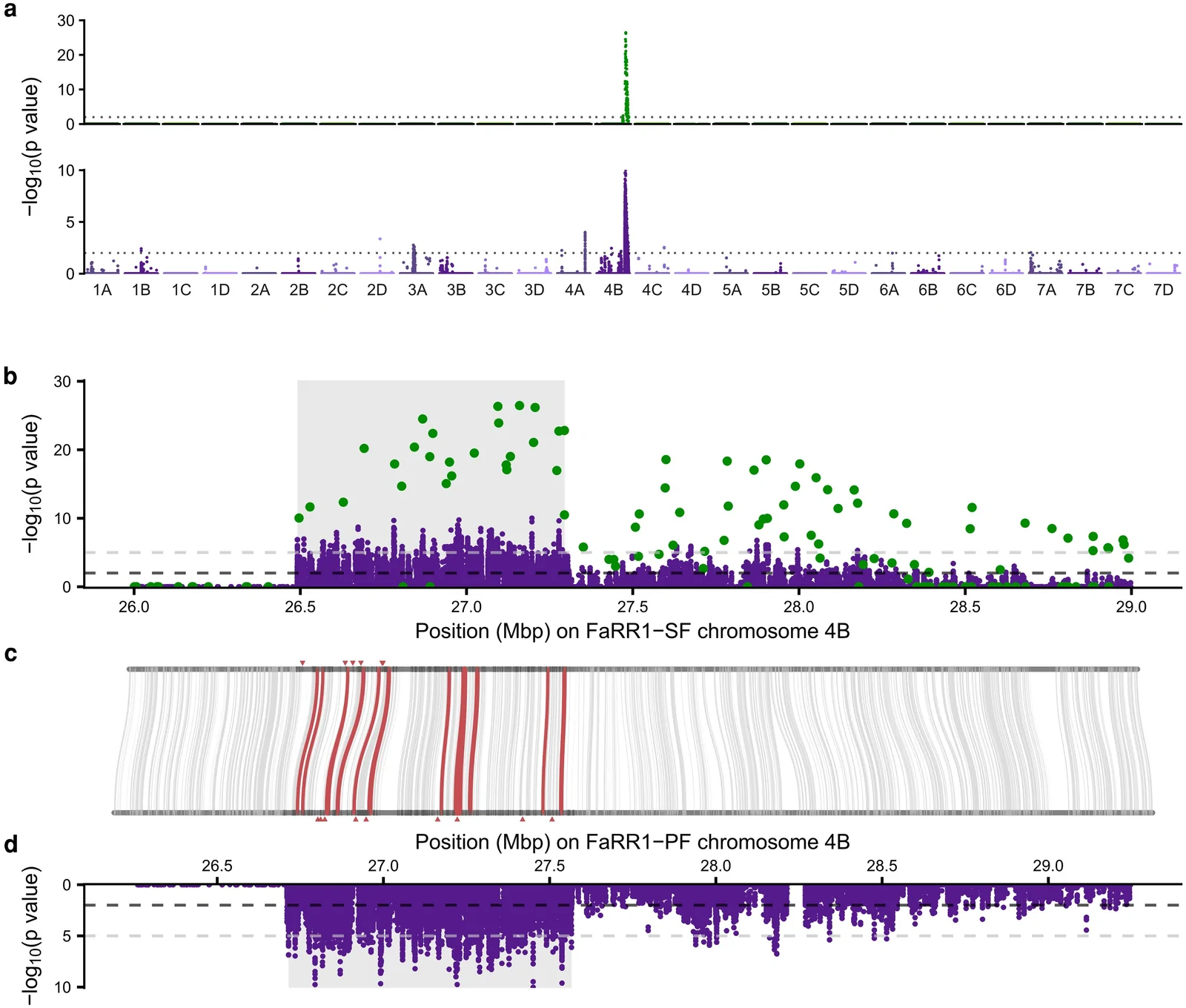
*k*-mer GWAS agrees with SNP GWAS but provides greater precision and flexibility. a) Axiom FanaSNP 50-k SNP array genotyping of 833 accessions reveals a single peak on chromosome 4B (above), also found in (below) *k*-mer GWAS of 137 resequenced accessions. b) Focusing on the 3-Mbp peak region confirms the concordance and highlights the precision of *k*-mer GWAS, used to define a peak region (gray box). c) Synteny between FaRR1-SF (panel b) and FaRR1-PF (panel d) highlights global similarity but a proliferation of sequence differences in the PF region. d) PF-associated *k*-mers mapped to FaRR1-PF. Homologs between haplotypes are connected by curved gray lines, red lines denote annotations with predicted high-effect sequence differences between haplotypes, and red triangles highlight annotations present in one haplotype and absent in the other. Dotted black lines indicate *P* = 0.01, and dotted gray line indicates *P* = 0.00001 used to delineate peak region.

### Extensive variation between SF and PF haplotypes inside and outside of annotated gene models

The PF locus peak region as defined by *k*-mer GWAS contains a total of 157 genes across both haplotypes; 139 genes have clear homologous copies on both haplotypes and 18 genes have copies unique to either the FaRR1-SF or FaRR1-PF haplotype (Table S4). These may represent true differences in gene content, but differences in annotation alone have not been ruled out. Comparison of “Royal Royce” WGS reads to FaRR1-PF revealed 3,416 single nucleotide or small indel variants between FaRR1-SF and FaRR1-PF in the PF region. Of these variants, 490 lie in predicted coding regions—the impacts of these variants were annotated using SNPeff (Cingolani et al 2012). Fifteen genes in the peak region on FaRR1-PF were predicted to have one or more high-impact variants (eg splice site or stop codon altered, Table S5), while 99 were predicted to have moderate-impact variants (eg amino acid change). Of these 2 sets of genes, 12 overlap and contain both variants with predicted high and moderate impact. We cannot rule out that some of these changes reflect alternative splicing, and not altered protein structure, between haplotypes. The preponderance of putative protein differences between FaRR1-SF and FaRR1-PF reflects the evolutionary distance between cultivated strawberry and the *F. virginiana* ssp. *glauca* donor of the “Wasatch” *PF* allele and linked DNA—extensive work has shown that a large region has survived years of breeding with very little recombination (Brukental et al 2025). Unfortunately, this large number of potentially impactful sequence-level variants in addition to presence-absence, promoter, and structural variation between haplotypes did not support a clear causal variant between FaRR1-PF and FaRR1-SF.

### RNAseq highlights circadian regulation in both PF and SF strawberry

To better understand differences between flowering control in SF and PF strawberry, and how the PF/PFRU locus alters this process, we undertook an RNA sequencing approach using 2 recently released UC cultivars: “UCD Victor” (“Victor,” *pfpf)* and “UCD Mojo” (“Mojo,” *PFPF*). Both cultivars were grown as runner-derived clones under long days and sampled at 10 weeks, a time at which only strawberry with a PF flowering habit is thought to be differentiating floral meristems (Perrotte et al 2016b). Because alteration of flowering regulation by day length is frequently associated with changes in circadian regulation (Yang et al 2024), we examined the transcriptomes of both cultivars over a 24-h time-course (6 time points, collected every 4 h). Finally, because *PF* could act through a difference in day length perception or a difference in signal transduction and physiological response, we examined the transcriptome of both leaves and crowns, the latter being the central stem of the strawberry plant containing the shoot apical meristem and axillary meristems (Fig. 2a). To improve the recall of potentially interesting genes in the PF region, bulk reads from this experiment representing all genotypes, tissues, and timepoints were used to manually adjust the annotation of the functionally annotated FaRR1-SF. This resulted in 13 new transcribed region annotations, and 11 new annotations derived from splitting incorrectly fused gene models (Tables S4 and S6). These new annotations may include protein-coding genes or other transcribed products, such as lncRNAs, and were not subjected to SNPeff analysis, although a putative coding sequence (CDS) was BLASTed to suggest protein function if translated (Table S4).

**Figure 2:**
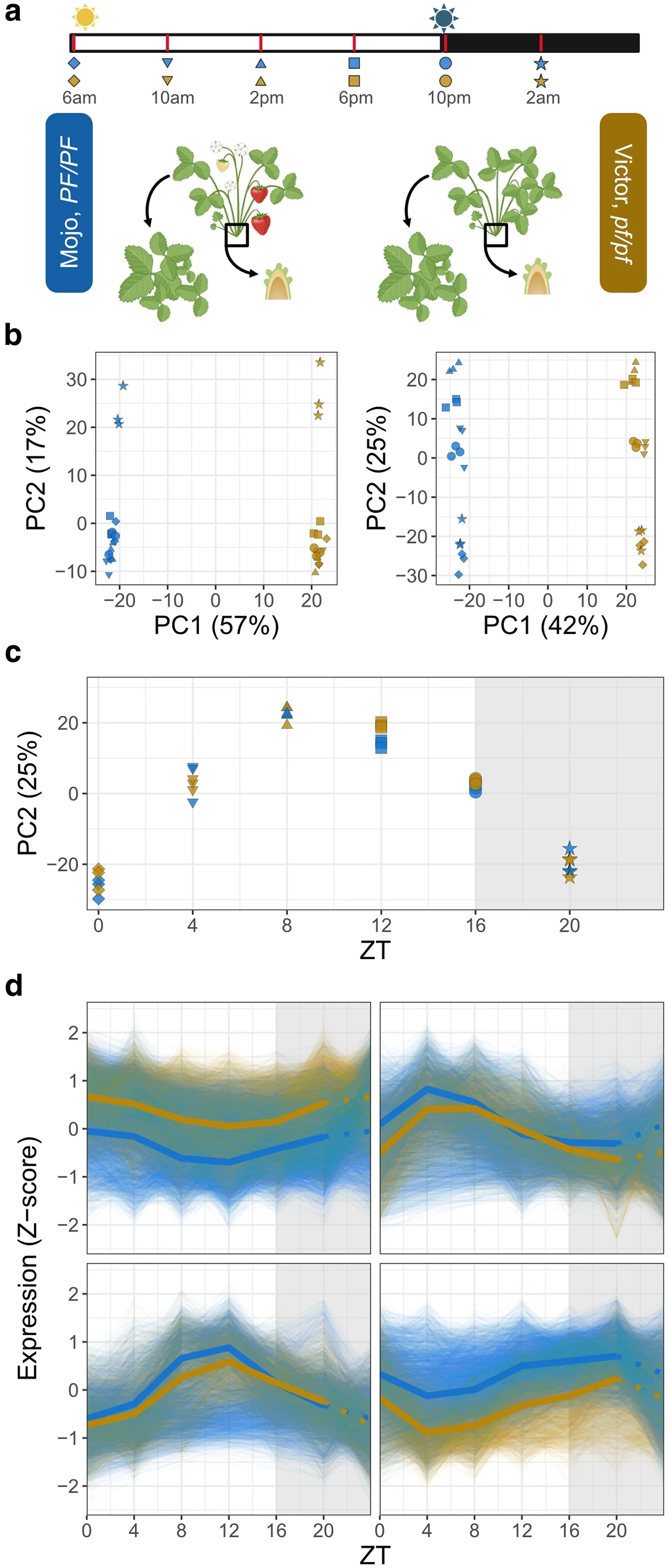
RNAseq reflects strong and similar circadian regulation in PF and SF leaves and crowns. a) Schematic of RNA-seq experiment. Leaves and crowns of “Victor” (*pfpf)* and “Mojo” (*PFPF)* were harvested for RNAseq over a 6-point time-course during long-day growth. b) Principal component analysis shows strong separation of “Victor” and “Mojo” in PC1 for both crowns (left) and leaves (right), with PC2 reflecting circadian-regulated transcriptome differences. c) Principal component 2 in leaves is strongly associated with time (ZT; zeitgeber time). d) In clusters with peak expression at dawn, mid-morning, mid-afternoon, or night, the crown transcriptomes of “Victor” and “Mojo” behave similarly. In panels (b) and (c), each point represents one sample; in panel (d) the expression of individual genes in a cluster at each time is connected by semi-transparent thin line, while cluster average gene expression at each time is connected by a bold line. Expression from ZT20—ZT24 is connected by a dotted line to represent an assumed return to the expression level measured the previous dawn. Panel A modified from BioRender.

Principal component analysis revealed strong differences between leaf and crown transcriptomes, obscuring within-tissue differences. When examining the 2 tissues separately, the primary discriminating factor was genotype (principal component 1, 42% to 57% variation explained). Principal component 2 (17 to 25% variation explained) aligned with collection time (Fig. 2b). The influence of time of day, and thus circadian rhythm, was particularly striking in leaves (Fig. 2c), as would be expected given the light perception and harvesting function of that organ.

We found a total of 10,095 differentially expressed genes (DEGs) between “Mojo” and “Victor” in either crowns or leaves in at least one timepoint (|log_2_(fold change)| *>* 1, *P*  *<* 0.05, Table S7). Of those, 3,111 were differentially expressed in both crowns and leaves, 2,850 DEGs were specific to leaves, and 4,134 DEGs were specific to crowns. Of the 5,961 DEGs found in leaves, 1,497 were found in all time points. Of the 7,245 DEGs found in crowns, 1,804 were found in all time points. A more comprehensive examination of DEGs across all possible timepoint intersections (Figure S2) showed that the largest set of DEGs between “Mojo” and “Victor” was the set shared among all timepoints and both tissues, with no evidence of global circadian dysregulation in either cultivar relative to the other.

To more deeply investigate potential differences in circadian regulation between “Mojo” (*PFPF*) and “Victor” (*pfpf)*, we examined expression patterns of members of the core circadian clock (Chen et al 2018; Faehn et al 2023; Ishibashi et al 2024). All identified members of the clock behaved similarly between this study and others, and between “Mojo” and “Victor” (Figure S3, Table S8). We further evaluated and compared the rhythmicity of gene expression in “Mojo” and “Victor.” We found 50,802 genes, the majority of expressed genes, to have rhythmic expression in either accession or tissue: 21,474 of these were common across both tissues with 16,681 found rhythmic only in leaves and 12,647 found rhythmic only in crowns. Across both tissues, 26,259 genes were found rhythmic in both “Mojo” and “Victor,” while 13,875 were found rhythmic only in “Mojo” and 10,668 only in “Victor.” To fully investigate these genes, we clustered the expression patterns of those found to be rhythmic in both genotypes in either tissue and found that clusters overall had similar amplitude and phase (Fig. 2d, Figure S4). Taken together, these results suggest that in both leaf and crown tissue, the day length is being sensed similarly in PF and SF accessions.

We finally examined differential gene expression in the PF peak region to identify candidate genes underlying PF locus regulation of flowering. Of 168 genes, including new annotations in the PF region (FaRR1-SF 26.5 to 27.3 Mbp), 26 were differentially regulated at one or more time points in leaves, and 30 were differentially regulated at one or more time points in crowns. Eleven of these DEGs were shared; thus, 45 genes—a little more than a quarter of the genes in the PF region—were differentially regulated between “Victor” and “Mojo” during at least one time in at least one tissue (Table S4). These genes encompass a wide range of suggested molecular functions, with no obvious single candidate gene connected to flowering pathways in model organisms, and may not encompass all the expression differences in SF vs PF meristems as the relatively coarse crown tissue sampling we used may dilute meristematic expression differences. “Victor” and “Mojo” are cousins, sharing the grandparent “05C165P001,” and might be expected to have more similar transcriptomes. To investigate whether the number of DEGs in the PF region was typical, we divided the FaRR1 annotation into 168-gene bins, to match the number of genes in the PF locus. This resulted in 403 bins with expression detected in this experiment. Of these, only 36 (9%) contained 45 or more DEGs. Thus, similar to the sequence-level differences, movement of a large segment of DNA from *F. virginiana* has led to many conserved expression-level changes, obscuring potentially causative differences.

### Flowering pathway expression diverges at *TFL1*

To directly assess the effect of the PF locus regulation of flowering, we examined the expression patterns of the central flowering pathway in “Mojo” and “Victor” (Koskela et al 2012; Gaston et al 2021). *FT1* expression was observed primarily in leaves of both accessions, from all homoeologs, with the least expression from subgenome D, and peaks in the evening, consistent with previous reports in *F. vesca* and *F.* × *ananassa* (Koskela et al 2012; Rantanen et al 2014; Nakano et al 2015). Downstream of *FT1*, *SOC1* was found at similar levels in both leaves and crowns, with slightly more expression in “Victor” than “Mojo,” though the difference was only significant for the subgenome D homoeolog in the crown and for this gene was significant at all time points. Strikingly, however, *TFL1* expression was only observed in crowns of “Victor” (average pseudocount *<* 10 in “Mojo,” log_2_(fold change) ranges from −2.4 to −10 in “Mojo” compared to “Victor”), and primarily from subgenome D. Correspondingly, the flowering reporter *AP1* was only observed in crowns of “Mojo” (Fig. 3). In *F. vesca*, FvFT2 is reported to induce flowering, despite low expression in crowns, and FvFT3 to regulate shoot branching (Gaston et al 2021); we did not detect expression of any homoeolog of either gene in either cultivar or tissue. These results indicate that *TFL1* expression in crowns is the critical step delineating PF from SF cultivars at the time of floral initiation under long days, and furthermore that TFL1-D may play the most significant role.

**Figure 3:**
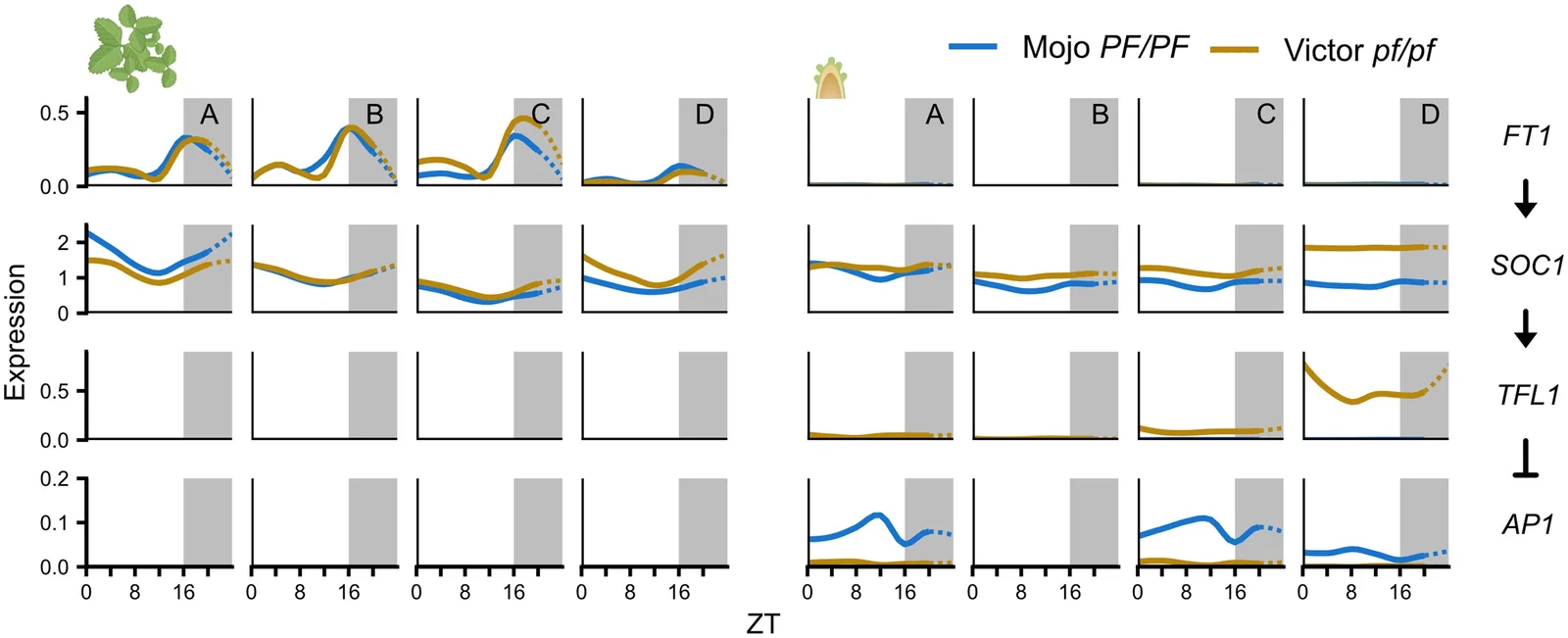
Flowering pathway gene expression diverges at *TFL1*. The average gene expression (×1,000, pseudocounts) for “Victor” (yellow) and “Mojo” (blue) is shown across the day/night cycle for key genes in flowering regulation. Expression of 4 homoeologs across the subgenomes of octoploid strawberry (A, B, C, D) are shown for leaves (left) and crowns (right). Tissue collection timepoints were 6 AM = ZT0, 10 AM, 2 PM, 6 PM (light) and 10 PM, 2 AM (darkness, gray background). Expression from 2 AM to 6 AM is connected by a dotted line to represent an assumed return to the expression level measured the previous dawn.

To further explore this observation, we used 2 other RNAseq datasets. In the first, we examined crown tissue of 4 genotypes: 2 SF (*pfpf*) siblings and 2 homozygous PF (*PFPF*) siblings, each with one high-runnering and one low-runnering individual. Three replicate crowns of each individual were harvested in a greenhouse under long days for RNA-seq. The dominant PF allele has a negative effect on runner production, although there is wide variability within each genotypic class (*PFPF*, *PFpf*, or *pfpf*, Gaston et al 2013; Brukental et al 2025). In these individuals *TFL1* levels correlated with flowering habit (Spearman's *ρ* = −0.87; *P_ρ_* = 5 × 10^−16^) but had no significant correlation with ordinal high/low runnering phenotype (Spearman's *ρ* = 0.04; *P_ρ_* = 0.81, Figure S5, Table S9). Next, we extended these results to field-grown plants, using RNAseq of runner tips from accessions “Camarosa” (*pfpf*), “Victor” (*pfpf*), and “Royal Royce” (*PFpf*) harvested during long days of summer. Notably, in these samples *TFL1* was expressed at a moderate level in all samples, and mostly from subgenome C, with expression also in subgenomes A and D (Figure S5, Table S9). In other species TFL1 acts to suppress juvenile flowering (Kotoda and Wada 2005); these results suggest that TFL1, and perhaps specifically TFL1-C, may play a similar role in *F.* × *ananassa*. Thus, differential *TFL1/TFL1-D* expression between SF and PF may become visible specifically in mature crowns during the period of floral transition.

### *TFL1* expression is an accurate predictor of flowering across PF sources

To assess how widely *TFL1* expression predicts flowering habit, we assayed *TFL1* expression in a panel of 7 SF and 8 PF accessions. These accessions were phenotyped, tracking inflorescences produced under long days, and genotyped with 2 DNA markers: “Bx215,” the most accurate single SSR marker in the PF locus (Perrotte et al 2016a; Lewers et al 2019), and “PF002,” a KASP marker commonly used in the UC Davis breeding program tracking a SNP in the PF locus (Table S10). The 7 SF accessions included 3 heirloom SF cultivars (“Earliglow,” “Sparkle,” and “Howard 17”), 2 recently released varieties (“Warrior” and “Victor”), a historic breeding individual (“71C098P605”) and “Stevens Pass,” a *Fragaria virginiana* subsp. *platypetala* ecotype, each with flowering habit phenotypes that were incorrectly predicted by one of these DNA markers. We also included UC cultivars with “Wasatch”-source perpetual flowering correctly predicted by DNA markers, as well as 2 accessions predicted from pedigrees to carry a “Rockhill”-source perpetual flowering allele (Fig. 4a). In all these genetic backgrounds, the number of inflorescences was negatively correlated with *TFL1* expression (Spearman *ρ* = −0.9, *P_ρ_* = 3.24 × 10^−6^, Fig. 4b and 4c). Hence, we found that *TFL1* expression was an accurate predictor of flowering habit across PF sources.

**Figure 4:**
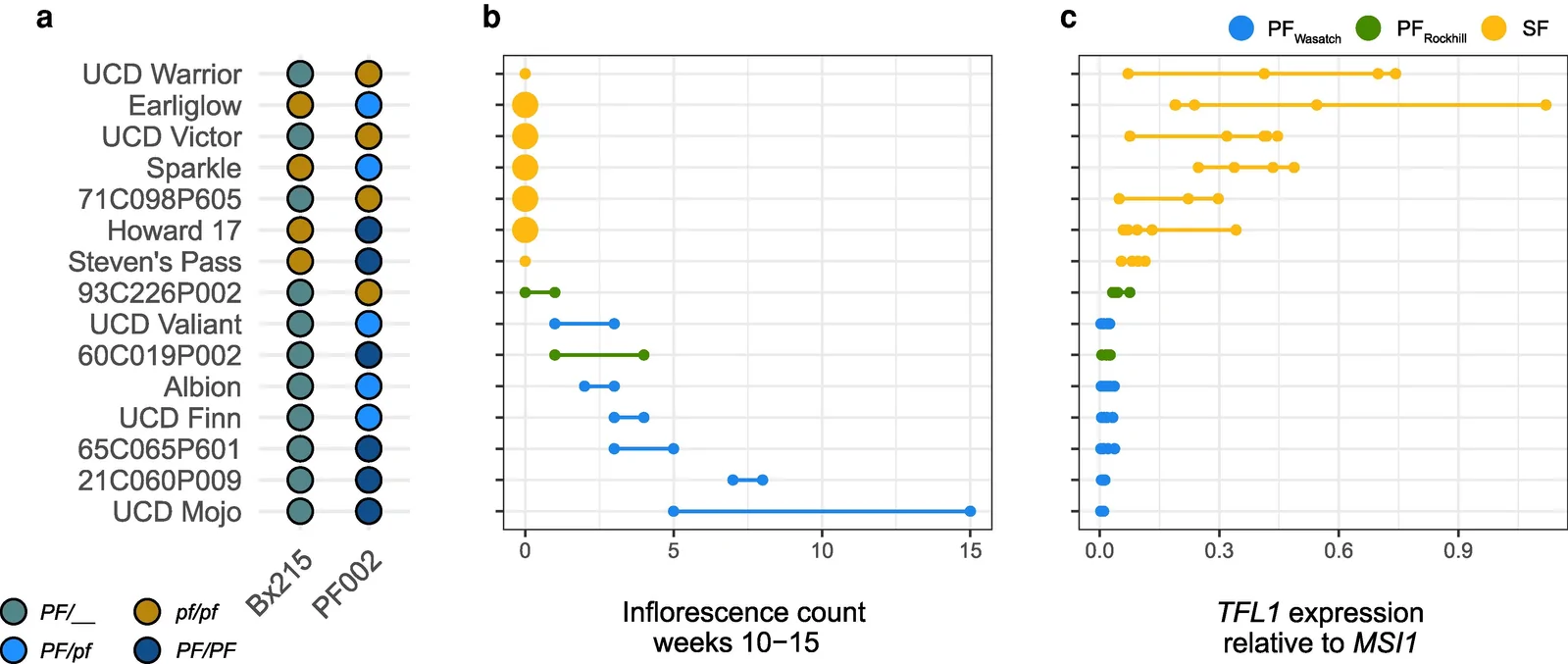
*TFL1* expression is negatively correlated with long-day-initiated inflorescences. a) Genotype by common PF markers. b) Inflorescences produced during assay period. c) *TFL1* expression in crowns measured at 10 weeks. Points are colored by marker-measured genotype (panel a) or by observed phenotype and theoretical donor of PF (panels b and c) and point size in panel b corresponds to the number of clones observed with a particular inflorescence count (1 or 2).

### Gene editing to modify flowering habit in *F.* × *ananassa*

RNAi of *TFL1* has been shown to induce perpetual flowering without affecting runnering in *F.* × *ananassa* (Koskela et al 2016). However, the construct to induce RNAi was developed from *F. vesca* and should suppress all 4 homoeologs in *F.* × *ananassa*, without subgenome resolution. Conversely, *FT2* has recently been shown to be necessary for floral induction in *F. vesca*, despite low expression, and it is unknown whether this same pathway may occur in *F.* × *ananassa* (Gaston et al 2021). To address these questions, and to assess whether CRISPR-Cas9 could be used to generate PF plants with a directly tracked causative locus, we undertook to edit *TFL1* (predicted to cause perpetual flowering phenotypes) and *FT2* (predicted to cause non-flowering phenotypes) in *F.* × *ananassa* cultivars “Royal Royce” (*PFpf*) and “Camarosa” (*pfpf*). These cultivars were selected for their contrasting flowering habits, genetic resources, and ease of transformation.

We obtained at least one large truncation of each homoeolog of each gene in each accession (Fig. 5a and 5d, Figure S6). Edited plants were genotyped in the greenhouse, subjected to an artificial winter, and phenotyped over 15 weeks. Inflorescences emerging during the first 10 weeks were considered to have been initiated during cool, short-day conditions pre-phenotyping, dependent on FT2 (Fig. 5b and 5e), while inflorescences emerging during weeks 10 to 15 were considered to have undergone floral initiation under warm long days, induced by FT1 and inhibited by TFL1 (Fig. 5c and 5f). “Royal Royce” plants edited in *ft2* produced fewer inflorescences in the first 10 weeks (Dunnett's test *P* = 0.001, Fig. 5a and 5c). In weeks 10 to 15, when inflorescences induced by *FT1* emerge (Fig. 3), *ft2* and unedited “Royal Royce” plants produced a similar number of inflorescences. A similar trend, though not significant, was observed in “Camarosa” in weeks 1 to 10 (Figure S6). Conversely, “Camarosa” plants edited in *tfl1* produced significantly more inflorescences than unedited control plants under long-day conditions (Dunnett's test *P* = 0.0496, Fig. 5d and 5f), although statistical certainty in this result is reduced by low sample size of unedited “Camarosa.” In “Royal Royce,” which is not expected to express *TFL1* based on its *PFpf* genotype, no significant differences were observed (Figure S6).

**Figure 5:**
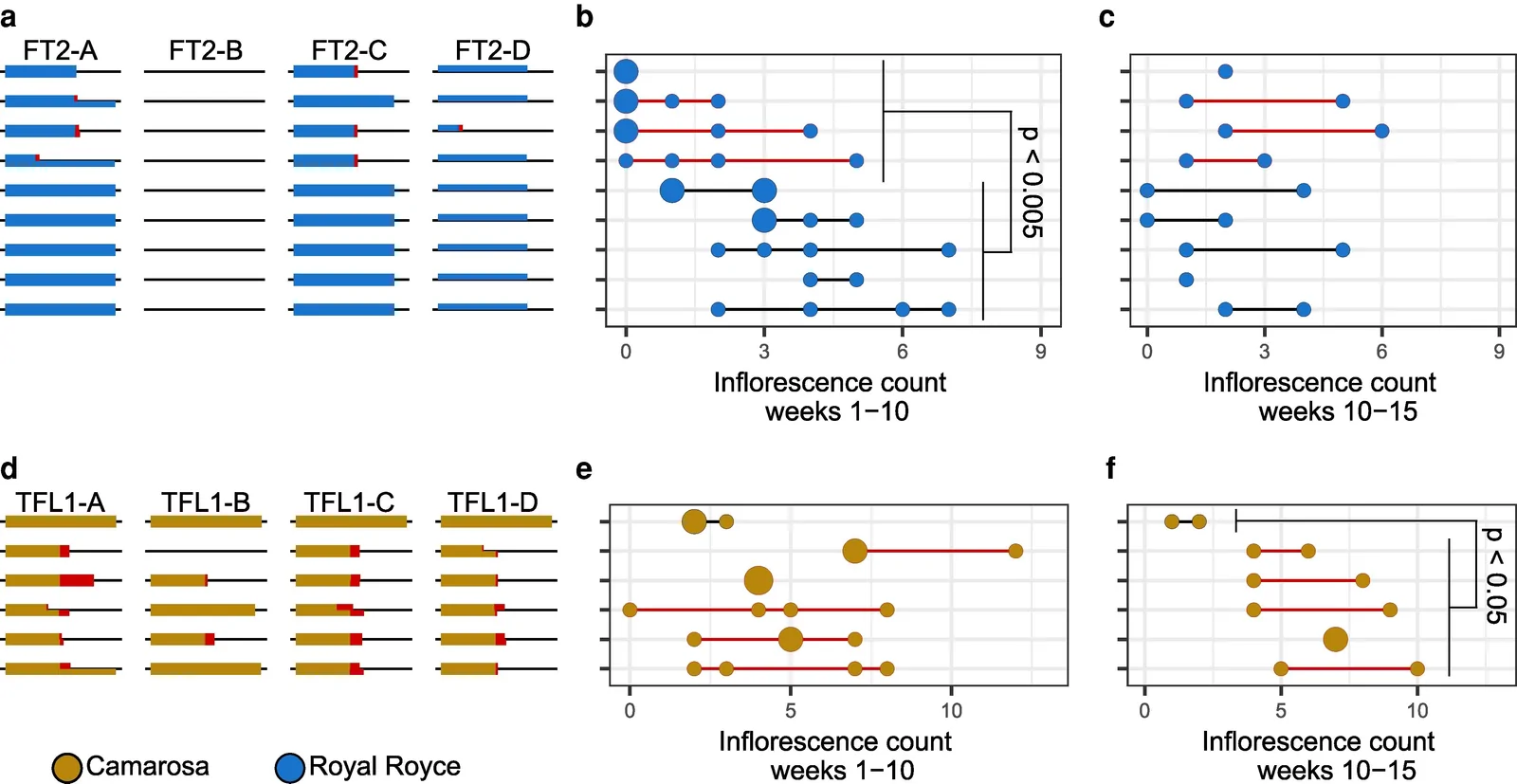
Gene editing of flowering pathway genes modifies *F.*  *×*  *ananassa* flowering behavior. a, d) Diagram of FT2 (a) or TFL1 (d) protein from the A, B, C, and D subgenomes in each assayed genotype (row) of “Camarosa” (*pfpf*, yellow) or “Royal Royce” (*PFpf*, blue). Yellow or blue indicates parental sequence and red indicates mis-sense translation; a shorter box indicates a premature stop and smaller protein product. Where a plant is heterozygous or bi-allelic, the 2 products are represented above and below the horizontal black line. b, e) Inflorescences produced during weeks 1 to 10 of assay, expanding from floral meristems differentiated during cool short-day pre-experimental conditions. c, f) Inflorescences produced during weeks 10 to 15 of assay, expanding from floral meristems differentiated during warm long-day experimental conditions. In panels b, c, e, and f point size indicates the number of clones observed at a particular inflorescence count (1, 2, or 3); edited plants are outlined in red while unedited plants are outlined in black; significant differences from unedited control (ANOVA followed by Dunnett's post hoc comparison) are highlighted.

## Discussion

The PF trait has been transformative to the strawberry industry. Yet persistent challenges with inconsistent phenotyping, description, and genetic resources have impeded progress toward developing a deeper understanding of the genetics controlling this trait (Gaston et al 2013; Castro et al 2015; Perrotte et al 2016a, 2016b; Lewers et al 2019; Cockerton et al 2023; Saiga et al 2023). *k*-mer GWAS and the “Royal Royce” genome offer an unprecedented combination—ubiquitous tagging of significant variants combined with the ability to compare PF and SF haplotypes. *k*-mer GWAS has not previously been used in cultivated strawberry, and our results validate the method with longer *k*-mers in this octoploid crop but did not reveal a causative variant. Instead, they revealed another barrier: limited recombination and a high density of genes and variants throughout the “Wasatch” introgression. To break down this barrier will be a significant undertaking: in an assessment of more than 4,500 seedlings of 92 crosses segregating for PF (*PFpf × pfpf* or *PFpf × PFpf*) among 2023 breeding program populations, we found only 45 (1%) plants with high-confidence recombination events between 27.05 and 27.3 Mb on FaRR1-SF. Further compounding the issue, these plants did not help narrow the PF region of interest as haplotypic diversity in their pedigrees precluded informative interpretation of the data (Figure S7).

Despite the lingering difficulty in identifying causal variants, this study has advanced our understanding of the PF locus' flowering regulation through characterization of DNA variants and expression differences through the locus. Beyond the PF locus, SF and PF cultivars showed similar circadian regulation transcriptome-wide and of core clock components, suggesting that differential perception of daylength signals does not underlie differential flowering habit in octoploid strawberry. Indeed, the mechanism of perpetual flowering in *F.* × *ananassa* appears to be functionally similar to that of *F. vesca*, in that both involve an absence of expression of the flowering repressor *TFL1* (Iwata et al 2012; Koskela et al 2012). Similarly, although *FT2* expression is very low in both *F.* × *ananassa* and *F. vesca* crowns (Koskela et al 2012, no expression observed in this study), loss of functional *FT2* results in reduced or abolished flowering in both plants (Gaston et al 2021). An edited FT2 in *F. vesca* truncated to encode only the first 63 amino acids showed only a partial loss in flowering (Gaston et al 2021). In *F.* × *ananassa* edited here, most individuals retained 116 amino acids, ∼2/3 functional FT2; nevertheless, we observed a quantitative decrease in inflorescence induction under short days.

Although most studies have identified PF/PFRU on chromosome 4B (accounting for nomenclature variation) as the sole locus controlling perpetual flowering, some have found multiple loci, or variable sites on chromosome 4B (Gaston et al 2013; Lewers et al 2019; Cockerton et al 2023). This may be partially due to true differences in loci, if, for example, one study utilized “Wasatch”-source while another used “Rockhill”-source, “Gloede's seedling”-source, or uncharacterized PF sources. Further complicating the issue, modern strawberry breeding germplasm frequently has multiple PF sources in each plant's pedigree, making it difficult to determine which source controls the observed phenotype. Genetic characterization is certainly further confounded by phenotyping variation—particularly when experiencing stress, a strawberry plant may produce one or a few inflorescences even during non-inductive temperature/daylength regime. Moreover, control of strawberry flowering may differ in an individual plant's first seedling spring when compared to runner-derived clones (Lewers et al 2019). The strong correlation of *TFL1* (and particularly *TFL1-D*) expression with flowering habit, in individuals representing 2 sources of PF and where genetic markers do not make accurate predictions, may help future researchers untangle these complications and determine whether, and to what extent the sources of the PF trait are related to each other. The PF locus as defined here contains 18 putative transcription factors, with 9 showing either differential expression or coding sequence differences between “Mojo” and “Victor.” One of these may directly or indirectly regulate TFL1, providing new leverage to untangle this regulation. However, even this must be taken with caution, as young, runner-tip crowns expressed *TFL1* (and particularly *TFL1-C*) regardless of their PF genotype (Figure S5). This, and environmental effects, may explain why *TFL1* has been reported to be expressed in PF plants under long days (Muñoz-Avila et al 2022) or repressed in some SF plants under long days (Koskela et al 2016). Finally, as AP1 can exert negative feedback on TFL1 (Kaufmann et al 2010), and other transcription factors can integrate environmental signals to directly regulate flowering pathway genes (Huang et al 2025), we cannot rule out external flowering regulators leading to the expression observed here. Crown floral regulator expression through a long-scale time-course of strawberry plant maturation and in response to flowering inductive conditions may help to clarify this in the future.

Beyond understanding the flowering biology of *F.* × *ananassa*, the strong correlation of *TFL1* expression with flowering has biotechnology implications. While the dominant “Wasatch” PF allele enables nearly year-round flowering, it can reduce vegetative vigor and runner count (Perrotte et al 2016a). In contrast to these results, recent work has shown that extensive variation in runner production exists in each genotypic class (*PFPF*, *PFpf*, or *pfpf*) (Brukental et al 2025), and that *PFPF* individuals can have extremely high vigor and yield (Knapp et al 2023). Nevertheless, the dominant *PF* allele may still be associated with negative agronomic traits in PF strawberry, and neither *tfl1* mutation in *F. vesca* nor *tfl1* knockdown by RNAi are reported to affect runner production (Koskela et al 2012, 2016). In this study, we observed low overall runner production, possibly related to the artificial winter required by transgenic plant containment. However, consistent with previous publication, *TFL1* editing did not appear to affect the minimal runners observed (Koskela et al 2016; Welch two-sample *t* test *t* = −0.33, *p_t_* = 0.76). Our results and others must be taken with caution, as flowering and runnering are also highly environmentally determined; the artificial winter and growth-chamber phenotyping may not be representative of strawberry performance under the agronomic conditions of plant or fruit production.

The gene editing approach used here offers biological and regulatory advantages over the previously used RNAi approach—the first relating to subgenome specificity. Through RNAseq, we were able to identify that *TFL1-D* accounts for most *TFL1* gene expression. A key question then arises: whether *TFL1-A*, -*B*, or -*C* are sufficient to repress flowering in the absence of *TFL1-D*. Likely at least one is sufficient, or natural variation in *TFL1-D* would lead to an alternate, chromosome 6D-localized PF source. Unfortunately, our CRISPR construct targeting all 4 *TFL1* homoeologs had high editing efficiency, and all assayed plants had edits in all subgenomes. However, some plants contain only 1-to-3 amino acid deletions in TFL1-A and/or TFL1-B, which may encode a functional protein. This suggests that the A and B subgenomes, at least, may not be sufficient to repress long-day flowering.

CRISPR-Cas9 gene editing also provides advantages in deployment. Gene edits can be easily tracked and used for marker-assisted selection via high-throughput markers like KASP, facilitating traditional hybridization-based breeding of a new synthetic PF. This gene edit could be selected independently of Cas9 transgene, and face minimal regulatory hurdles in much of the world. This will be particularly valuable if only *TFL1-D* editing is necessary to induce PF, although it becomes more complicated if multiple loci must be selected together. If multiple edits are needed, editing could be done directly in a strawberry accession intended for commercial release. For this approach, a transgene-free editing technique would be required to avoid transgenic regulation, but PF could be directly engineered into well-known and beloved, or recent and elite seasonal flowering strawberry cultivars.

The perpetual flowering trait enables near-year-round strawberry production around the world and has been the focus of decades of genetic mapping studies. Despite the presence of a well-characterized and long-studied genetic locus, the molecular mechanism remains a mystery. Through this work we have confirmed a critical flowering step at *TERMINAL FLOWER 1* regulated by the PF locus in cultivated strawberry and have shown that *TFL1* is both a key to understanding natural variation and a viable target for gene editing to produce synthetic perpetual flowering in strawberry varieties.

## Experimental procedures

### GWAS

*k*-mer GWAS was performed primarily according to the kGWAS pipeline, with some modifications (Gaurav et al 2022). Briefly, *k*-mers of length 31, 51, or 101 were counted from Illumina sequencing data (Hardigan et al 2020), and compiled into a *k*-mer presence/absence table for all accessions using scripts developed for Cavalet-Giorsa et al (2024) and published at GitHub. To reduce computational burden, *k*-mers were prefiltered to those with a Spearman correlation coefficient greater than 0.2 or less than −0.2. Prefiltered *k*-mers were then associated with flowering habit using gemma (Zhou and Stephens 2012) with a kinship matrix calculated from FanaSNP genotype data on the same accessions using rrBLUP (Endelman 2011). For visualization, *k*-mers were mapped to the Royal Royce genome using bowtie2 with the parameters -p 8 -r -D 24 -R 3 -N 0 -L 18 -i S,1,0.30 (Langmead and Salzberg 2012), and mappings filtered by mapping quality (q40) with samtools (Danecek et al 2021).

SNP GWAS was performed using rrBLUP (Endelman 2011) and a population of 833 accessions phenotyped for PF and genotyped using the Axiom FanaSNP 50-k array (Hardigan et al 2020; Brukental et al 2025, Tables S1 and S2).

SNP and *k*-mer association *P* values were corrected for multiple testing using the p.adjust function in R (R Core Team 2025) with method “Bonferroni.”

### Haplotype comparisons

To evaluate similarity and differences between *k*-mer tagged regions on FaRR1-SF vs FaRR1-PF, we first calculated homologous genes genome-wide using GENESPACE (Lovell et al 2022). Microsynteny at the PF region was then visualized using JCVI (Tang et al 2024). To evaluate variants that may affect protein function, WGS reads of Royal Royce were aligned to FaRR1-PF using bwa-mem (Li 2013) and mappings filtered to mapping quality > 40. Variants were called using bcftools (Danecek et al 2021) and filtered to quality > 20 and read depth > 10. Putative effects of variants were predicted using SNPeff (Cingolani et al 2012).

### Manual annotation updates

To update annotation in the PF region, the first replicate of each genotype/tissue combination was combined (6 samples from 6 timepoints) and aligned to FaRR1. Alignments from these 4 files were manually scanned across the PF region, and new transcribed regions added to the FaRR1 gtf file (Table S6). Simultaneously, several existing annotations were identified to be gene fusions (no reads aligned across an annotated intron) and corrected to 2 independent annotations. Transcribed regions that did not encode at least 100 bp without a stop codon in at least one frame were discarded, and the longest ORF identified in a transcribed region was searched using blastx against the ClusteredNR database at NCBI.

*TFL1* homoeolog annotation was updated to correct an inaccurate splice site based on *F. vesca* homology and RNAseq read alignment (Data S1).

### Plant growth and RNA extraction

For the circadian time-course and *TFL1* expression panel, bare-root clones of the specified accessions were dug from the Wolfskill Experimental Orchard in Winters, CA in December/January, trimmed, and stored at −2 °C. Experiments were begun within 3 months when clones were removed from storage and potted in SunGro media. For CRISPR edited plants, *in vitro* plantlets were acclimated to growth chamber and then greenhouse conditions. Four clones of each plant of interest were obtained by rooting runners. Once established, runners were removed from mother plants and subjected to artificial winter: 9 weeks of short days, with temperatures gradually shifting from 20/20 °C to 10/4 °C day/night. Runners were then trimmed, repotted, and transferred to evaluation chamber.

Plants were grown under 16 h light/8 h dark at 18 °C, 40% humidity, light intensity 200 to 250 µE. Flowers and runners were trimmed and counted approximately weekly, as all genotypes produced flowers initiated prior to digging. Plants were destructively sampled for leaf and crown tissue when SF individuals had stopped producing inflorescences and PF individuals were just beginning to increase inflorescence production—at 9 to 10 weeks. For the circadian time-course, 2 plants for each genotype were combined into one sample, and 3 samples collected per timepoint (6 AM, 10 AM, 2 PM, 6 PM, 10 PM, 2 AM). The dawn and dusk time points were collected just after the chamber lights had turned on or off, respectively. For assessing *TFL1* expression in diverse accessions, 5 plants were started; 3 harvested as individual samples for RNA at 10 weeks and 2 kept for subsequent phenotyping. Each leaf sample consisted of a collection of mixed old and young leaves; for crowns the apical ∼0.5 cm of tissue was collected, containing apical/axillary meristem and stem tissue without further dissection (Koembuoy et al 2017). This sampling technique was used, rather than apical meristem dissection, to balance meristematic tissue enrichment with sampling time for rapid harvesting. In the case of multiple crowns in one plant (branch crowns), all crowns were harvested into one sample. All tissue was flash-frozen in liquid nitrogen and stored at −80 °C.

For high- and low-runnering siblings, plants were grown in a greenhouse and crowns harvested in January, with supplemental light to maintain 16/8 h light/dark and supplemental heat to maintain >15 °C. For runner tips, plants were grown in-field at Wolfskill Experimental Orchard. Runner tips were harvested on 20 July 2020; day length was ∼14.5/8.5 h light/dark and temperatures ranged from 14 to 30 °C. To harvest runner tips the distal 2 cm of an immature stolon was selected, prior to leaf or root primordia emergence, and immediately frozen in liquid nitrogen.

### RNA extraction, sequencing, and analysis

For RNAseq and reverse transcription quantitative PCR (RT-qPCR), frozen samples were ground in liquid nitrogen with mortar and pestle and RNA extracted as in Blanco-Ulate et al (2013) with some modifications. Briefly, 100 to 200 mg tissue was suspended in 800 µL extraction buffer (2 M NaCl, 100 mM Tris-HCl, 25 mM EDTA, 2% CTAB, 2% PVP, 0.05% spermidine, 0.02% beta-mercaptoethanol) per 100 mg tissue and incubated at 65 °C for 5 min, followed by 2 chloroform:isoamyl alcohol (24:1) extractions. RNA was precipitated in 2.6 M LiCl at −20 °C for 2 days, followed by ethanol precipitation. For RNA sequencing, RNA was further cleaned via Qiagen RNA column purification.

### RNAseq analysis

RNA sequencing was performed at Novogene. All samples passed RNA integrity and concentration quality control and were sequenced on NovaSeq to a depth of >20 million 150 bp paired-end reads per sample. RNAseq reads were assessed for quality with FastQC (Andrews 2010) and aligned to FaRR1 (Hardigan et al 2021) using HiSat2 (Kim et al 2019). Approximately 76% to 78% of reads mapped uniquely to one position (subgenome specific), and these reads were assigned to genes using featureCounts in R (Liao et al 2014; R Core Team 2025). Leaf and crown samples were analyzed independently in DeSeq2 using the model “counts ∼ Genotype + Time” and individual contrasts of interest specified to obtain DEGs for comparisons between genotypes at each time (Love et al 2014). Gene counts were subjected to a variance-stabilizing transformation, and pseudocounts used for visualization. Principal component analysis was performed with prcomp() in R, accessed through the plotPCA() wrapper of DESEq2.

To evaluate and compare circadian expression patterns we used the package circacompare in R with pseudocounts as input data (Parsons et al 2020). To investigate circadian clock genes, we used a combination of homolog identification from *F. vesca* using GENESPACE and Blast-Align-Tree to identify candidates based on *Arabidopsis thaliana* and *F. vesca* homology (Sheppard et al 2026).

### RT-qPCR

For RT-qPCR, cDNA was synthesized with RevertAid reverse transcriptase (ThermoFisher EP0441) and oligo-dT priming. cDNA was diluted 1:40 and 4 µL were used as template with 1 µL primer mix and 5 µL 2× PowerUp qPCR mastermix (ThermoFisher A25472). The qPCR cycle conditions were as follows: 95 °C 20 s denature; 40 cycles of 95 °C 1 s, 60 °C 20 s; melting rise from 60 °C to 95 °C at 0.15 °C/s. *MSI1* was used as reference gene (Koskela et al 2016); TFL1 primers were designed to amplify all homoeologous copies of *TFL1*, modified from Koskela et al (2016) with efficiency 96.3%. All primer sequences are in Table S10.

### PF markers

PF002 KASP marker was used to genotype selected accessions (Table S10) with 2× KASP mastermix (LGC Biosearch Technologies), 2% PVP in a 10 µL reaction using the standard KASP protocol on a QuantStudio3 (Applied Biosystems). For Bx215, PCR was run in a total volume of 10 µL consisting of 1× OneTaq Hot Start buffer, 0.20 mM each dNTP, 0.05 µM forward primer labeled with 6-FAM fluorescent reporter, 0.05 µM unlabeled reverse primer (Table S10), 0.25 U OneTaq Hot Start DNA Polymerase (New England Biolabs), and 2 µL diluted template DNA. Amplification consisted of a 3-step cycling procedure consisting of initial denaturation at 94 °C for 3 min, 40 cycles of denaturation at 94 °C for 40 s, annealing at 58 °C for 45 s, and extension at 72 °C for 45 s followed by a final extension at 72 °C for 7 min as modified from Perrotte et al (2016a).

We conducted capillary electrophoresis using 0.5 µL of the resulting PCR product mixed with 6 µL Hi-Di Formamide and 0.2 µL GeneScan 600 LIZ size standard (ThermoFisher Scientific/Life Technologies). Samples were denatured at 96 °C for 10 min followed by chilling for 10 min at −20 °C, then loaded on to a 3,500 × L Genetic Analyzer (ThermoFisher Scientific/Life Technologies) with a 50-cm capillary array and POP-7 matrix. Electropherograms were scored using STRand Analysis Software v2.4.110 (Toonen and Hughes 2001; UC Davis Veterinary Genetics Laboratory).

PCR produced multiple amplicons per sample: several samples had amplicons of both 128 bp (marker for perpetual flowering) and 130 bp with inconsistent amplification. Typically, if the shorter amplicon has weaker amplification than the longer amplicon, it is disregarded as an artifact of DNA polymerase. Samples where this pattern was consistent across multiple runs, were not scored as having the 128-bp allele. However, for multiple samples, the amplification strength of the 128- and 130-bp amplicons were nearly equal and fluctuated across runs, such that scoring was inconsistent. For inconsistent samples, composite scores were based on the data quality and the score seen more frequently.

### CRISPR-Cas9 edited plant generation and analysis

To knock out components of the flowering pathway, we used Chop-Chop to generate guides and selected 2 guides for each gene (Table S10), each of which targeted conserved sequence among the 4 homoeologs (Labun et al 2019). The two-guide construct was cloned via BsaI digestion/ligation into a binary vector developed in-house at the UC Davis Plant Transformation facility using MoClo assembly (Weber et al 2011). This binary vector confers resistance to kanamycin in bacteria; in the T-DNA region the vector contained NptII conferring kanamycin resistance in planta with NOS promoter/terminator, an *Arabidopsis thaliana* codon optimized Cas9 with a 5′ UTR intron of the castor bean *cat1* gene under the control of the 35 s constitutive promoter, 2 guide RNAs with associated scaffold sequence driven by a single U6 and connected by tRNA linker for in planta cleavage (Tanaka et al 1990; Xie et al 2015; Castel et al 2019). Transgenic strawberry plants in the “Royal Royce” (*PFpf)* and “Camarosa” (*pfpf)* backgrounds were generated by the UC Davis Plant Transformation Facility. Transformants were genotyped through amplicon sequencing using a Golang implementation of the CRISpy pipeline (Connelly and Pruett-Miller 2019; Zhang 2020). Individual plants transformed with Cas9 yet not containing edits in the genes of interest were selected as controls (Data S1).

To assess inflorescence differences, individual runner-derived clones were treated as independent observations of each edited/unedited genotype. Genotypes were nested within the categories of non-edited, *TFL1*-edited, or *FT2*-edited for statistical comparisons. We then performed ANOVA followed by Dunnett's test comparing *TFL1*-edited or *FT2*-edited plants to shared non-edited controls within each genotype-time period combination.

## Supplementary Material

kiag684_Supplementary_Data

## Data Availability

WGS data first published here are available at the SRA under bioproject PRJNA1418671, and time-course RNAseq data of “Mojo” and “Victor” crowns are available at PRJNA1417769.
