# Supplementary material for: CRISPR editing of TERMINAL FLOWER 1 recapitulates perpetual flowering in strawberry, mimicking repression by the PERPETUAL FLOWERING locus
Source: Plant Physiol. 2026 Oct 9;202(2):kiag684. doi: 10.1093/plphys/kiag684 (PMC13650783; doi:10.1093/plphys/kiag684)
Supplement: Supplementary file 1 [file kiag684_supplementary_data.zip › DataS1_CRISPRfasta.docx]

**Dataset S1.** Pseudofasta of CRISPR edits in FT2 and TFL1. For each of FT2-A, FT2-B, FT2-C, FT2-D, TFL1-A, TFL1-D, TFL1-C, TFL1-D, the FaRR1 DNA sequence is presented first with gRNA sequences highlighted in blue, PAM in green, and exons in capital letters while introns are lowercase. Translated protein sequences are immediately below the respective DNA sequences. If an edited line is biallelic or heterozygous, both haplotypes are presented. In case edits cannot be confidently phased (i.e. biallelic or heterozygous edits at both sites), the hap1 and hap2 sequence IDs are highlighted in yellow. If homozygous, only one sequence is presented with the label ‘hap1-2’. CRISPR edits are highlighted in bold text, with insertions in red, deletions represented by ‘-‘, and missense amino acid translation in red.

>LineXXXX_FT2-A_DNA

ATGTATCAGCTTTTGGCTATATATACTCGGTGGCTTGTGTTTTCAATTCAGTTAATGCTTAAATCTATGGCGAGGGCTAGAGATCAGGAGCCTCTTGTCGTCGGGAGAGTGATCGGAGATGTTATAGAGCCGTTCACGAAGTCTGTGTCTTTGAGGATGACTTACAGTAATAACAGGGAGGTTACCAGTGGCTGTGAGCTTAAACCTTCTCATGTTATCAACCGACCTCGAGTTCAGATAGGCGGAGATGATCTTAGGAACTTCTACACTCTGgtaagcaattacttgcatacatatacccatctgtatattgcatgcatatatcatgctctttgtcttgtttgtttagcctacagcttccatttatactttattgcagGTCATGGTAGATCCTGATGCGCCCAGTCCAAGTGATCCCAACCTCAAGGAATACTTGCATTGgtaatattgctctgttttccctctttttagttatcaaacgcataggagatagcaaaagcatgagctataagccctgatacgtagtattattgcatgctaagctttttgaggtttcgaattccatcttcaagtcaagatggcaagaactctaaggaaacgatctagctagaccaagaaggatatgtgtaaaggagaagaacatgcatccgttcattttgattgattaattttcttttttatttcagaattagatatgtctacgctaggttgatgatgattgaggtattcatcctttacatgttttttcagGTTGGTTACTGATATTCCGGCAACAACTGGAGCAAGCTTCGgtgagtacttctacatttcatccactttgttgaattgggttacttctaataccagCAAGATACTATGTAAACGCACCAACTCATGCAGGCCAAGAGATTGTGAGCTATGAAAGTCCAAGGCCATCTATTGGGATCCATCGCTTTGTTTCGGTTTTGTTTCGCCAATTGGGAAGGCAAACAGTGTATGCTCCAGGATGGCGCCAAAATTTTAACACCAGAGAGTTTGCTGAGAACTATAATCTTGGATCACCGGTGGCCGCTGTCTTTTTTAACTGCCAGAGGGAGAGCGGCTCCGGTGGAAGGAGAATGTAG

>LineXXXX_FT2-A_protein

MYQLLAIYTRWLVFSIQLMLKSMARARDQEPLVVGRVIGDVIEPFTKSVSLRMTYSNNREVTSGCELKPSHVINRPRVQIGGDDLRNFYTLVMVDPDAPSPSDPNLKEYLHWLVTDIPATTGASFARYYVNAPTHAGQEIVSYESPRPSIGIHRFVSVLFRQLGRQTVYAPGWRQNFNTREFAENYNLGSPVAAVFFNCQRESGSGGRRM*

>LineC17_FT2-A_DNA

ATGTATCAGCTTTTGGCTATATATACTCGGTGGCTTGTGTTTTCAATTCAGTTAATGCTTAAATCTATGGCGAGGGCTAGAGATCAGGAGCCTCTTGTCGTCGGGAGAGTGATCGGAGATGTTATAGAGCCGTTCACGAAGTCTGTGTCTTTGAGGATGACTTACAGTAATAACAGGGAGGTTACCAGTGGCTGTGAGCTTAAACCTTCTCATGTTATCAACCGACCTCGAGTTCAGATAGGCGGAGATGATCTTAGGAACTTCTACACTCTGgtaagcaattacttgcatacatatacccatctgtatattgcatgcatatatcatgctctttgtcttgtttgtttagcctacagcttccatttatactttattgcagGTCATGGTAGATCCTGATGCGCCCAGTCCAAGTGATCCCAACCTCAAGGAATACTTGCATTGgtaatattgctctgttttccctctttttagttatcaaacgcataggagatagcaaaagcatgagctataagccctgatacgtagtattattgcatgctaagctttttgaggtttcgaattccatcttcaagtcaagatggcaagaactctaaggaaacgatctagctagaccaagaaggatatgtgtaaaggagaagaacatgcatccgttcattttgattgattaattttcttttttatttcagaattagatatgtctacgctaggttgatgatgattgaggtattcatcctttacatgttttttcagGTTGGTTACTGATATTCCGGCAACAACTGGAGCAAGCTTCGgtgagtacttctacatttcatccactttgttgaattgggttacttctaataccagCAAGATACTAT**GTAAACGCACCAACTCA‑GCAGG**CCAAGAGATTGTGAGCTATGAAAGTCCAAGGCCATCTATTGGGATCCATCGCTTTGTTTCGGTTTTGTTTCGCCAATTGGGAAGGCAAACAGTGTATGCTCCAGGATGGCGCCAAAATTTTAACACCAGAGAGTTTGCTGAGAACTATAATCTTGGATCACCGGTGGCCGCTGTCTTTTTTAACTGCCAGAGGGAGAGCGGCTCCGGTGGAAGGAGAATGTAG

>LineC17_FT2-A_protein

MYQLLAIYTRWLVFSIQLMLKSMARARDQEPLVVGRVIGDVIEPFTKSVSLRMTYSNNREVTSGCELKPSHVINRPRVQIGGDDLRNFYTLVMVDPDAPSPSDPNLKEYLHWLVTDIPATTGASFARYYVNAPT**QQAKRL***

>Line09_FT2-A_DNA_hap1

ATGTATCAGCTTTTGGCTATATATACTCGGTGGCTTGTGTTTTCAATTCAGTTAATGCTTAAATCTATGGCGAGGGCTAGAGATCAGGAGCCTCTTGTCGTCGGGAGAGTGATCGGAGATGTTATAGAGCCGTTCACGAAGTCTGTGTCTTTGAGGAT**GACTTACAGTAATAACAAGGGAGG**TTACCAGTGGCTGTGAGCTTAAACCTTCTCATGTTATCAACCGACCTCGAGTTCAGATAGGCGGAGATGATCTTAGGAACTTCTACACTCTGgtaagcaattacttgcatacatatacccatctgtatattgcatgcatatatcatgctctttgtcttgtttgtttagcctacagcttccatttatactttattgcagGTCATGGTAGATCCTGATGCGCCCAGTCCAAGTGATCCCAACCTCAAGGAATACTTGCATTGgtaatattgctctgttttccctctttttagttatcaaacgcataggagatagcaaaagcatgagctataagccctgatacgtagtattattgcatgctaagctttttgaggtttcgaattccatcttcaagtcaagatggcaagaactctaaggaaacgatctagctagaccaagaaggatatgtgtaaaggagaagaacatgcatccgttcattttgattgattaattttcttttttatttcagaattagatatgtctacgctaggttgatgatgattgaggtattcatcctttacatgttttttcagGTTGGTTACTGATATTCCGGCAACAACTGGAGCAAGCTTCGgtgagtacttctacatttcatccactttgttgaattgggttacttctaataccagCAAGATACTATGTAAACGCACCAACTCATGCAGGCCAAGAGATTGTGAGCTATGAAAGTCCAAGGCCATCTATTGGGATCCATCGCTTTGTTTCGGTTTTGTTTCGCCAATTGGGAAGGCAAACAGTGTATGCTCCAGGATGGCGCCAAAATTTTAACACCAGAGAGTTTGCTGAGAACTATAATCTTGGATCACCGGTGGCCGCTGTCTTTTTTAACTGCCAGAGGGAGAGCGGCTCCGGTGGAAGGAGAATGTAG

>Line09_FT2-A_protein_hap1

MYQLLAIYTRWLVFSIQLMLKSMARARDQEPLVVGRVIGDVIEPFTKSVSLRMTYSNN**KGGYQWL***

>Line09_FT2-A_DNA_hap2

ATGTATCAGCTTTTGGCTATATATACTCGGTGGCTTGTGTTTTCAATTCAGTTAATGCTTAAATCTATGGCGAGGGCTAGAGATCAGGAGCCTCTTGTCGTCGGGAGAGTGATCGGAGATGTTATAGAGCCGTTCACGAAGTCTGTGTCTTTGAGGATGACTTACAGTAATAACAGGGAGGTTACCAGTGGCTGTGAGCTTAAACCTTCTCATGTTATCAACCGACCTCGAGTTCAGATAGGCGGAGATGATCTTAGGAACTTCTACACTCTGgtaagcaattacttgcatacatatacccatctgtatattgcatgcatatatcatgctctttgtcttgtttgtttagcctacagcttccatttatactttattgcagGTCATGGTAGATCCTGATGCGCCCAGTCCAAGTGATCCCAACCTCAAGGAATACTTGCATTGgtaatattgctctgttttccctctttttagttatcaaacgcataggagatagcaaaagcatgagctataagccctgatacgtagtattattgcatgctaagctttttgaggtttcgaattccatcttcaagtcaagatggcaagaactctaaggaaacgatctagctagaccaagaaggatatgtgtaaaggagaagaacatgcatccgttcattttgattgattaattttcttttttatttcagaattagatatgtctacgctaggttgatgatgattgaggtattcatcctttacatgttttttcagGTTGGTTACTGATATTCCGGCAACAACTGGAGCAAGCTTCGgtgagtacttctacatttcatccactttgttgaattgggttacttctaataccagCAAGATACTATGTAAACGCACCAACT‑‑‑GCAGGCCAAGAGATTGTGAGCTATGAAAGTCCAAGGCCATCTATTGGGATCCATCGCTTTGTTTCGGTTTTGTTTCGCCAATTGGGAAGGCAAACAGTGTATGCTCCAGGATGGCGCCAAAATTTTAACACCAGAGAGTTTGCTGAGAACTATAATCTTGGATCACCGGTGGCCGCTGTCTTTTTTAACTGCCAGAGGGAGAGCGGCTCCGGTGGAAGGAGAATGTAG

>Line09_FT2-A_protein_hap2

MYQLLAIYTRWLVFSIQLMLKSMARARDQEPLVVGRVIGDVIEPFTKSVSLRMTYSNNREVTSGCELKPSHVINRPRVQIGGDDLRNFYTLVMVDPDAPSPSDPNLKEYLHWLVTDIPATTGASFARY

YVNAPTAGQEIVSYESPRPSIGIHRFVSVLFRQLGRQTVYAPG**‑**WRQNFNTREFAENYNLGSPVAAVFFNCQRESGSGGRRM*

>Line34_FT2-A_DNA_hap1

ATGTATCAGCTTTTGGCTATATATACTCGGTGGCTTGTGTTTTCAATTCAGTTAATGCTTAAATCTATGGCGAGGGCTAGAGATCAGGAGCCTCTTGTCGTCGGGAGAGTGATCGGAGATGTTATAGAGCCGTTCACGAAGTCTGTGTCTTTGAGGATGACTTACAGTAATAACAGGGAGGTTACCAGTGGCTGTGAGCTTAAACCTTCTCATGTTATCAACCGACCTCGAGTTCAGATAGGCGGAGATGATCTTAGGAACTTCTACACTCTGgtaagcaattacttgcatacatatacccatctgtatattgcatgcatatatcatgctctttgtcttgtttgtttagcctacagcttccatttatactttattgcagGTCATGGTAGATCCTGATGCGCCCAGTCCAAGTGATCCCAACCTCAAGGAATACTTGCATTGgtaatattgctctgttttccctctttttagttatcaaacgcataggagatagcaaaagcatgagctataagccctgatacgtagtattattgcatgctaagctttttgaggtttcgaattccatcttcaagtcaagatggcaagaactctaaggaaacgatctagctagaccaagaaggatatgtgtaaaggagaagaacatgcatccgttcattttgattgattaattttcttttttatttcagaattagatatgtctacgctaggttgatgatgattgaggtattcatcctttacatgttttttcagGTTGGTTACTGATATTCCGGCAACAACTGGAGCAAGCTTCGgtgagtacttctacatttcatccactttgttgaattgggttacttctaataccagCAAGATACTAT**GTAAACGCACCAACTC‑TGCAGG**CCAAGAGATTGTGAGCTATGAAAGTCCAAGGCCATCTATTGGGATCCATCGCTTTGTTTCGGTTTTGTTTCGCCAATTGGGAAGGCAAACAGTGTATGCTCCAGGATGGCGCCAAAATTTTAACACCAGAGAGTTTGCTGAGAACTATAATCTTGGATCACCGGTGGCCGCTGTCTTTTTTAACTGCCAGAGGGAGAGCGGCTCCGGTGGAAGGAGAATGTAG

>Line34_FT2-A_protein_hap1

MYQLLAIYTRWLVFSIQLMLKSMARARDQEPLVVGRVIGDVIEPFTKSVSLRMTYSNNREVTSGCELKPSHVINRPRVQIGGDDLRNFYTLVMVDPDAPSPSDPNLKEYLHWLVTDIPATTGASFARYYVNAPT**LQAKRL***

>Line34_FT2-A_DNA_hap2

ATGTATCAGCTTTTGGCTATATATACTCGGTGGCTTGTGTTTTCAATTCAGTTAATGCTTAAATCTATGGCGAGGGCTAGAGATCAGGAGCCTCTTGTCGTCGGGAGAGTGATCGGAGATGTTATAGAGCCGTTCACGAAGTCTGTGTCTTTGAGGATGACTTACAGTAATAACAGGGAGGTTACCAGTGGCTGTGAGCTTAAACCTTCTCATGTTATCAACCGACCTCGAGTTCAGATAGGCGGAGATGATCTTAGGAACTTCTACACTCTGgtaagcaattacttgcatacatatacccatctgtatattgcatgcatatatcatgctctttgtcttgtttgtttagcctacagcttccatttatactttattgcagGTCATGGTAGATCCTGATGCGCCCAGTCCAAGTGATCCCAACCTCAAGGAATACTTGCATTGgtaatattgctctgttttccctctttttagttatcaaacgcataggagatagcaaaagcatgagctataagccctgatacgtagtattattgcatgctaagctttttgaggtttcgaattccatcttcaagtcaagatggcaagaactctaaggaaacgatctagctagaccaagaaggatatgtgtaaaggagaagaacatgcatccgttcattttgattgattaattttcttttttatttcagaattagatatgtctacgctaggttgatgatgattgaggtattcatcctttacatgttttttcagGTTGGTTACTGATATTCCGGCAACAACTGGAGCAAGCTTCGgtgagtacttctacatttcatccactttgttgaattgggttacttctaataccagCAAGATACTATGTAAACGCACCA‑‑TCATGCAGGCCAAGAGATTGTGAGCTATGAAAGTCCAAGGCCATCTATTGGGATCCATCGCTTTGTTTCGGTTTTGTTTCGCCAATTGGGAAGGCAAACAGTGTATGCTCCAGGATGGCGCCAAAATTTTAACACCAGAGAGTTTGCTGAGAACTATAATCTTGGATCACCGGTGGCCGCTGTCTTTTTTAACTGCCAGAGGGAGAGCGGCTCCGGTGGAAGGAGAATGTAG

>Line34_FT2-A_protein_hap2

MYQLLAIYTRWLVFSIQLMLKSMARARDQEPLVVGRVIGDVIEPFTKSVSLRMTYSNNREVTSGCELKPSHVINRPRVQIGGDDLRNFYTLVMVDPDAPSPSDPNLKEYLHWLVTDIPATTGASFARY

YVNAP**SCRPRDCEL***

>Line07_FT2-A_DNA

ATGTATCAGCTTTTGGCTATATATACTCGGTGGCTTGTGTTTTCAATTCAGTTAATGCTTAAATCTATGGCGAGGGCTAGAGATCAGGAGCCTCTTGTCGTCGGGAGAGTGATCGGAGATGTTATAGAGCCGTTCACGAAGTCTGTGTCTTTGAGGATGACTTACAGTAATAACAGGGAGGTTACCAGTGGCTGTGAGCTTAAACCTTCTCATGTTATCAACCGACCTCGAGTTCAGATAGGCGGAGATGATCTTAGGAACTTCTACACTCTGgtaagcaattacttgcatacatatacccatctgtatattgcatgcatatatcatgctctttgtcttgtttgtttagcctacagcttccatttatactttattgcagGTCATGGTAGATCCTGATGCGCCCAGTCCAAGTGATCCCAACCTCAAGGAATACTTGCATTGgtaatattgctctgttttccctctttttagttatcaaacgcataggagatagcaaaagcatgagctataagccctgatacgtagtattattgcatgctaagctttttgaggtttcgaattccatcttcaagtcaagatggcaagaactctaaggaaacgatctagctagaccaagaaggatatgtgtaaaggagaagaacatgcatccgttcattttgattgattaattttcttttttatttcagaattagatatgtctacgctaggttgatgatgattgaggtattcatcctttacatgttttttca‑‑‑unknown large deletion‑‑‑ACCAGAGAGTTTGCTGAGAACTATAATCTTGGATCACCGGTGGCCGCTGTCTTTTTTAACTGCCAGAGGGAGAGCGGCTCCGGTGGAAGGAGAATGTAG

>Line07_FT2-A_protein

MYQLLAIYTRWLVFSIQLMLKSMARARDQEPLVVGRVIGDVIEPFTKSVSLRMTYSNNREVTSGCELKPSHVINRPRVQIGGDDLRNFYTLVMVDPDAPSPSDPNLKEYLHWLVTDIPATTGASF

>Line32_FT2-A_DNA_hap1

ATGTATCAGCTTTTGGCTATATATACTCGGTGGCTTGTGTTTTCAATTCAGTTAATGCTTAAATCTATGGCGAGGGCTAGAGATCAGGAGCCTCTTGTCGTCGGGAGAGTGATCGGAGATGTTATAGAGCCGTTCACGAAGTCTGTGTCTTTGAGGATGACTTACAGTAATAACAGGGAGGTTACCAGTGGCTGTGAGCTTAAACCTTCTCATGTTATCAACCGACCTCGAGTTCAGATAGGCGGAGATGATCTTAGGAACTTCTACACTCTGgtaagcaattacttgcatacatatacccatctgtatattgcatgcatatatcatgctctttgtcttgtttgtttagcctacagcttccatttatactttattgcagGTCATGGTAGATCCTGATGCGCCCAGTCCAAGTGATCCCAACCTCAAGGAATACTTGCATTGgtaatattgctctgttttccctctttttagttatcaaacgcataggagatagcaaaagcatgagctataagccctgatacgtagtattattgcatgctaagctttttgaggtttcgaattccatcttcaagtcaagatggcaagaactctaaggaaacgatctagctagaccaagaaggatatgtgtaaaggagaagaacatgcatccgttcattttgattgattaattttcttttttatttcagaattagatatgtctacgctaggttgatgatgattgaggtattcatcctttacatgttttttcagGTTGGTTACTGATATTCCGGCAACAACTGGAGCAAGCTTCGgtgagtacttctacatttcatccactttgttgaattgggttacttctaataccagCAAGATACTAT**GTAAACGCAC‑‑‑‑‑‑‑‑‑‑AGG**CCAAGAGATTGTGAGCTATGAAAGTCCAAGGCCATCTATTGGGATCCATCGCTTTGTTTCGGTTTTGTTTCGCCAATTGGGAAGGCAAACAGTGTATGCTCCAGGATGGCGCCAAAATTTTAACACCAGAGAGTTTGCTGAGAACTATAATCTTGGATCACCGGTGGCCGCTGTCTTTTTTAACTGCCAGAGGGAGAGCGGCTCCGGTGGAAGGAGAATGTAG

>Line32_FT2-A_protein_hap1

MYQLLAIYTRWLVFSIQLMLKSMARARDQEPLVVGRVIGDVIEPFTKSVSLRMTYSNNREVTSGCELKPSHVINRPRVQIGGDDLRNFYTLVMVDPDAPSPSDPNLKEYLHWLVTDIPATTGASFARYYVNA**QAKRL***

>Line32_FT2-A_DNA_hap2

ATGTATCAGCTTTTGGCTATATATACTCGGTGGCTTGTGTTTTCAATTCAGTTAATGCTTAAATCTATGGCGAGGGCTAGAGATCAGGAGCCTCTTGTCGTCGGGAGAGTGATCGGAGATGTTATAGAGCCGTTCACGAAGTCTGTGTCTTTGAGGATGACTTACAGTAATAACAGGGAGGTTACCAGTGGCTGTGAGCTTAAACCTTCTCATGTTATCAACCGACCTCGAGTTCAGATAGGCGGAGATGATCTTAGGAACTTCTACACTCTGgtaagcaattacttgcatacatatacccatctgtatattgcatgcatatatcatgctctttgtcttgtttgtttagcctacagcttccatttatactttattgcagGTCATGGTAGATCCTGATGCGCCCAGTCCAAGTGATCCCAACCTCAAGGAATACTTGCATTGgtaatattgctctgttttccctctttttagttatcaaacgcataggagatagcaaaagcatgagctataagccctgatacgtagtattattgcatgctaagctttttgaggtttcgaattccatcttcaagtcaagatggcaagaactctaaggaaacgatctagctagaccaagaaggatatgtgtaaaggagaagaacatgcatccgttcattttgattgattaattttcttttttatttcagaattagatatgtctacgctaggttgatgatgattgaggtattcatcctttacatgttttttcagGTTGGTTACTGATATTCCGGCAACAACTGGAGCAAGCTTCGgtgagtacttctacatttcatccactttgttgaattgggttacttctaataccagCAAGATACTATGTAAACGCACCAACTCATGCAGGCCAAGAGATTGTGAGCTATGAAAGTCCAAGGCCATCTATTGGGATCCATCGCTTTGTTTCGGTTTTGTTTCGCCAATTGGGAAGGCAAACAGTGTATGCTCCAGGATGGCGCCAAAATTTTAACACCAGAGAGTTTGCTGAGAACTATAATCTTGGATCACCGGTGGCCGCTGTCTTTTTTAACTGCCAGAGGGAGAGCGGCTCCGGTGGAAGGAGAATGTAG

>Line-32_FT2-A_protein_hap2

MYQLLAIYTRWLVFSIQLMLKSMARARDQEPLVVGRVIGDVIEPFTKSVSLRMTYSNNREVTSGCELKPSHVINRPRVQIGGDDLRNFYTLVMVDPDAPSPSDPNLKEYLHWLVTDIPATTGASFARYYVNAPTHAGQEIVSYESPRPSIGIHRFVSVLFRQLGRQTVYAPGWRQNFNTREFAENYNLGSPVAAVFFNCQRESGSGGRRM*

>LineXXXX_FT2-C_DNA

ATGCTTAAATCTATGGCGAGGGCTAGAGATCAGGAGCCTCTTGTCATCGGGAGAGTGATCGGAGATGTTATAGAGCCGTTCACGAAGTCTGTGTCTTTGAGGATGACTTACAGTAATAACAGGGAGGTTACCAGTGGCTGTGAGCTTAAACCTTCTCATGTTATCAACCGACCTCGAGTTCAGATAGGTGGAGATGATCTTAGGATCTTCTACACTCTGgtaagcaattacttgcatacatatacccatctgtatattgcatgcatatatcatgctctttgtcttgtttgtttagaaatgcacgagtttagcctacagcttccatttatacttgattgcagGTCATGGTAGATCCTGATGCGCCCAGTCCAAGTGATCCCAACCTCAAGGAATACTTGCATTGgtaatattgctctgttttccctctttttagttatcaaacgcacaggagagagcaaaagcatgagctataagccctgatacgtagtattattgcatgctaagctttttgacgtttcgaattccatcttcaagtcaagatggcaagaactctaaggaaacgatcttgctagaccaagaaggatatgtgtaaaggagaagaacatgcatccgttcattttgattgattaattttctttttatttcagaattagatatgtctacgctaggttgatgatgattgaggtattcatcctttacatgttttttcagGTTGGTTACTGATATTCCGGCAACAACTGGAGCAAGCTTCGgtgagtacttatacatttcatccactttgttgaatttgggttacttctaataccagCAAGATACTATGTAAACGCACCAACTCATGCAGGCCAAGAGATTTTGAGCTATGAAAGTCCAAGGCCATCTATGGGGATCCATCGCTTTGTTTTGGTTTTGTTTCACCAATTGGGAAGGCAAACAGTGTATGCTCCAGGATGGCGCCAAAATTTTAACACCAGAGAGTTTGCTGAGAACTGTAATCTTGGATCACCGGTGGCCGCTGTCTTTTGTAACTGCCAGAGGGAGAGCGGCTCCGGCGGAAGGAGAATGTAG

>LineXXXX_FT2-C_protein

MLKSMARARDQEPLVIGRVIGDVIEPFTKSVSLRMTYSNNREVTSGCELKPSHVINRPRVQIGGDDLRIFYTLVMVDPDAPSPSDPNLKEYLHWLVTDIPATTGASFARYYVNAPTHAGQEILSYESPRPSMGIHRFVLVLFHQLGRQTVYAPGWRQNFNTREFAENCNLGSPVAAVFCNCQRESGSGGRRM*

>LineC17_FT2-C_DNA_hap1

ATGCTTAAATCTATGGCGAGGGCTAGAGATCAGGAGCCTCTTGTCATCGGGAGAGTGATCGGAGATGTTATAGAGCCGTTCACGAAGTCTGTGTCTTTGAGGATGACTTACAGTAATAACAGGGAGGTTACCAGTGGCTGTGAGCTTAAACCTTCTCATGTTATCAACCGACCTCGAGTTCAGATAGGTGGAGATGATCTTAGGATCTTCTACACTCTGgtaagcaattacttgcatacatatacccatctgtatattgcatgcatatatcatgctctttgtcttgtttgtttagaaatgcacgagtttagcctacagcttccatttatacttgattgcagGTCATGGTAGATCCTGATGCGCCCAGTCCAAGTGATCCCAACCTCAAGGAATACTTGCATTGgtaatattgctctgttttccctctttttagttatcaaacgcacaggagagagcaaaagcatgagctataagccctgatacgtagtattattgcatgctaagctttttgacgtttcgaattccatcttcaagtcaagatggcaagaactctaaggaaacgatcttgctagaccaagaaggatatgtgtaaaggagaagaacatgcatccgttcattttgattgattaattttctttttatttcagaattagatatgtctacgctaggttgatgatgattgaggtattcatcctttacatgttttttcagGTTGGTTACTGATATTCCGGCAACAACTGGAGCAAGCTTCGgtgagtacttatacatttcatccactttgttgaatttgggttacttctaataccagCAAGATACTAT**GTAAACGCACCAACT‑ATGCAGG**CCAAGAGATTTTGAGCTATGAAAGTCCAAGGCCATCTATGGGGATCCATCGCTTTGTTTTGGTTTTGTTTCACCAATTGGGAAGGCAAACAGTGTATGCTCCAGGATGGCGCCAAAATTTTAACACCAGAGAGTTTGCTGAGAACTGTAATCTTGGATCACCGGTGGCCGCTGTCTTTTGTAACTGCCAGAGGGAGAGCGGCTCCGGCGGAAGGAGAATGTAG

>LineC17_FT2-C_protein_hap1

MLKSMARARDQEPLVIGRVIGDVIEPFTKSVSLRMTYSNNREVTSGCELKPSHVINRPRVQIGGDDLRIFYTLVMVDPDAPSPSDPNLKEYLHWLVTDIPATTGASFARYYVNAPT**MQAKRF***

>LineC17_FT2-C_DNA_hap2

ATGCTTAAATCTATGGCGAGGGCTAGAGATCAGGAGCCTCTTGTCATCGGGAGAGTGATCGGAGATGTTATAGAGCCGTTCACGAAGTCTGTGTCTTTGAGGATGACTTACAGTAATAACAGGGAGGTTACCAGTGGCTGTGAGCTTAAACCTTCTCATGTTATCAACCGACCTCGAGTTCAGATAGGTGGAGATGATCTTAGGATCTTCTACACTCTGgtaagcaattacttgcatacatatacccatctgtatattgcatgcatatatcatgctctttgtcttgtttgtttagaaatgcacgagtttagcctacagcttccatttatacttgattgcagGTCATGGTAGATCCTGATGCGCCCAGTCCAAGTGATCCCAACCTCAAGGAATACTTGCATTGgtaatattgctctgttttccctctttttagttatcaaacgcacaggagagagcaaaagcatgagctataagccctgatacgtagtattattgcatgctaagctttttgacgtttcgaattccatcttcaagtcaagatggcaagaactctaaggaaacgatcttgctagaccaagaaggatatgtgtaaaggagaagaacatgcatccgttcattttgattgattaattttctttttatttcagaattagatatgtctacgctaggttgatgatgattgaggtattcatcctttacatgttttttcagGTTGGTTACTGATATTCCGGCAACAACTGGAGCAAGCTTCGgtgagtacttatacatttcatccactttgttgaatttgggttacttctaataccagCAAGATACTATGTAAACGCACCAACTCA**‑**GCAGGCCAAGAGATTTTGAGCTATGAAAGTCCAAGGCCATCTATGGGGATCCATCGCTTTGTTTTGGTTTTGTTTCACCAATTGGGAAGGCAAACAGTGTATGCTCCAGGATGGCGCCAAAATTTTAACACCAGAGAGTTTGCTGAGAACTGTAATCTTGGATCACCGGTGGCCGCTGTCTTTTGTAACTGCCAGAGGGAGAGCGGCTCCGGCGGAAGGAGAATGTAG

>LineC17_FT2-C_protein_hap2

MLKSMARARDQEPLVIGRVIGDVIEPFTKSVSLRMTYSNNREVTSGCELKPSHVINRPRVQIGG

DDLRIFYTLVMVDPDAPSPSDPNLKEYLHWLVTDIPATTGASFARYYVNAPT**QQAKRF***

>Line09_FT2-C_DNA_hap1

ATGCTTAAATCTATGGCGAGGGCTAGAGATCAGGAGCCTCTTGTCATCGGGAGAGTGATCGGAGATGTTATAGAGCCGTTCACGAAGTCTGTGTCTTTGAGGATGACTTACAGTAATAACAGGGAGGTTACCAGTGGCTGTGAGCTTAAACCTTCTCATGTTATCAACCGACCTCGAGTTCAGATAGGTGGAGATGATCTTAGGATCTTCTACACTCTGgtaagcaattacttgcatacatatacccatctgtatattgcatgcatatatcatgctctttgtcttgtttgtttagaaatgcacgagtttagcctacagcttccatttatacttgattgcagGTCATGGTAGATCCTGATGCGCCCAGTCCAAGTGATCCCAACCTCAAGGAATACTTGCATTGgtaatattgctctgttttccctctttttagttatcaaacgcacaggagagagcaaaagcatgagctataagccctgatacgtagtattattgcatgctaagctttttgacgtttcgaattccatcttcaagtcaagatggcaagaactctaaggaaacgatcttgctagaccaagaaggatatgtgtaaaggagaagaacatgcatccgttcattttgattgattaattttctttttatttcagaattagatatgtctacgctaggttgatgatgattgaggtattcatcctttacatgttttttcagGTTGGTTACTGATATTCCGGCAACAACTGGAGCAAGCTTCGgtgagtacttatacatttcatccactttgttgaatttgggttacttctaataccagCAAGATACTATGTAAACGCACCAACTCA**‑**GCAGGCCAAGAGATTTTGAGCTATGAAAGTCCAAGGCCATCTATGGGGATCCATCGCTTTGTTTTGGTTTTGTTTCACCAATTGGGAAGGCAAACAGTGTATGCTCCAGGATGGCGCCAAAATTTTAACACCAGAGAGTTTGCTGAGAACTGTAATCTTGGATCACCGGTGGCCGCTGTCTTTTGTAACTGCCAGAGGGAGAGCGGCTCCGGCGGAAGGAGAATGTAG

>Line09_FT2-C_protein_hap1

MLKSMARARDQEPLVIGRVIGDVIEPFTKSVSLRMTYSNNREVTSGCELKPSHVINRPRVQIGG

DDLRIFYTLVMVDPDAPSPSDPNLKEYLHWLVTDIPATTGASFARYYVNAPT**QQAKRF***

>Line09_FT2-C_DNA_hap2

ATGCTTAAATCTATGGCGAGGGCTAGAGATCAGGAGCCTCTTGTCATCGGGAGAGTGATCGGAGATGTTATAGAGCCGTTCACGAAGTCTGTGTCTTTGAGGATGACTTACAGTAATAACAGGGAGGTTACCAGTGGCTGTGAGCTTAAACCTTCTCATGTTATCAACCGACCTCGAGTTCAGATAGGTGGAGATGATCTTAGGATCTTCTACACTCTGgtaagcaattacttgcatacatatacccatctgtatattgcatgcatatatcatgctctttgtcttgtttgtttagaaatgcacgagtttagcctacagcttccatttatacttgattgcagGTCATGGTAGATCCTGATGCGCCCAGTCCAAGTGATCCCAACCTCAAGGAATACTTGCATTGgtaatattgctctgttttccctctttttagttatcaaacgcacaggagagagcaaaagcatgagctataagccctgatacgtagtattattgcatgctaagctttttgacgtttcgaattccatcttcaagtcaagatggcaagaactctaaggaaacgatcttgctagaccaagaaggatatgtgtaaaggagaagaacatgcatccgttcattttgattgattaattttctttttatttcagaattagatatgtctacgctaggttgatgatgattgaggtattcatcctttacatgttttttcagGTTGGTTACTGATATTCCGGCAACAACTGGAGCAAGCTTCGgtgagtacttatacatttcatccactttgttgaatttgggttacttctaataccagCAAGATACTATGTAAACGCACCAACTC**‑**TGCAGGCCAAGAGATTTTGAGCTATGAAAGTCCAAGGCCATCTATGGGGATCCATCGCTTTGTTTTGGTTTTGTTTCACCAATTGGGAAGGCAAACAGTGTATGCTCCAGGATGGCGCCAAAATTTTAACACCAGAGAGTTTGCTGAGAACTGTAATCTTGGATCACCGGTGGCCGCTGTCTTTTGTAACTGCCAGAGGGAGAGCGGCTCCGGCGGAAGGAGAATGTAG

>Line09_FT2-C_protein_hap2

MLKSMARARDQEPLVIGRVIGDVIEPFTKSVSLRMTYSNNREVTSGCELKPSHVINRPRVQIGG

DDLRIFYTLVMVDPDAPSPSDPNLKEYLHWLVTDIPATTGASFARYYVNAPT**LQAKRF***

>Line34_FT2-C_DNA

ATGCTTAAATCTATGGCGAGGGCTAGAGATCAGGAGCCTCTTGTCATCGGGAGAGTGATCGGAGATGTTATAGAGCCGTTCACGAAGTCTGTGTCTTTGAGGAT**GACTTACAGTAATAACAGGGAGG**TTACCAGTGGCTGTGAGCTTAAACCTTCTCATGTTATCAACCGACCTCGAGTTCAGATAGGTGGAGATGATCTTAGGATCTTCTACACTCTGgtaagcaattacttgcatacatatacccatctgtatattgcatgcatatatcatgctctttgtcttgtttgtttagaaatgcacgagtttagcctacagcttccatttatacttgattgcagGTCATGGTAGATCCTGATGCGCCCAGTCCAAGTGATCCCAACCTCAAGGAATACTTGCATTGgtaatattgctctgttttccctctttttagttatcaaacgcacaggagagagcaaaagcatgagctataagccctgatacgtagtattattgcatgctaagctttttgacgtttcgaattccatcttcaagtcaagatggcaagaactctaaggaaacgatcttgctagaccaagaaggatatgtgtaaaggagaagaacatgcatccgttcattttgattgattaattttctttttatttcagaattagatatgtctacgctaggttgatgatgattgaggtattcatcctttacatgttttttcagGTTGGTTACTGATATTCCGGCAACAACTGGAGCAAGCTTCGgtgagtacttatacatttcatccactttgttgaatttgggttacttctaataccagCAAGATACTAT**GTAAACGCACCAAC‑‑‑‑GCAGG**CCAAGAGATTTTGAGCTATGAAAGTCCAAGGCCATCTATGGGGATCCATCGCTTTGTTTTGGTTTTGTTTCACCAATTGGGAAGGCAAACAGTGTATGCTCCAGGATGGCGCCAAAATTTTAACACCAGAGAGTTTGCTGAGAACTGTAATCTTGGATCACCGGTGGCCGCTGTCTTTTGTAACTGCCAGAGGGAGAGCGGCTCCGGCGGAAGGAGAATGTAG

>Line34_FT2-C_protein

MLKSMARARDQEPLVIGRVIGDVIEPFTKSVSLRMTYSNNREVTSGCELKPSHVINRPRVQIGGDDLRIFYTLVMVDPDAPSPSDPNLKEYLHWLVTDIPATTGASFARYYVNAPT**QAKRF***

>Line07_FT2-C_DNA

ATGCTTAAATCTATGGCGAGGGCTAGAGATCAGGAGCCTCTTGTCATCGGGAGAGTGATCGGAGATGTTATAGAGCCGTTCACGAAGTCTGTGTCTTTGAGGATGACTTACAGTAATAACAGGGAGGTTACCAGTGGCTGTGAGCTTAAACCTTCTCATGTTATCAACCGACCTCGAGTTCAGATAGGTGGAGATGATCTTAGGATCTTCTACACTCTGgtaagcaattacttgcatacatatacccatctgtatattgcatgcatatatcatgctctttgtcttgtttgtttagaaatgcacgagtttagcctacagcttccatttatacttgattgcagGTCATGGTAGATCCTGATGCGCCCAGTCCAAGTGATCCCAACCTCAAGGAATACTTGCATTGgtaatattgctctgttttccctctttttagttatcaaacgcacaggagagagcaaaagcatgagctataagccctgatacgtagtattattgcatgctaagctttttgacgtttcgaattccatcttcaagtcaagatggcaagaactctaaggaaacgatcttgctagaccaagaaggatatgtgtaaaggagaagaacatgcatccgttcattttgattgattaattttctttttatttcagaattagatatgtctacgctaggttgatgatgattgaggtattcatcctttacatgttttttcagGTTGGTTACTGATATTCCGGCAACAACTGGAGCAAGCTTCGgtgagtacttatacatttcatccactttgttgaatttgggttacttctaataccagCAAGATACTATGTAAACGCACCAACTCA**‑**GCAGGCCAAGAGATTTTGAGCTATGAAAGTCCAAGGCCATCTATGGGGATCCATCGCTTTGTTTTGGTTTTGTTTCACCAATTGGGAAGGCAAACAGTGTATGCTCCAGGATGGCGCCAAAATTTTAACACCAGAGAGTTTGCTGAGAACTGTAATCTTGGATCACCGGTGGCCGCTGTCTTTTGTAACTGCCAGAGGGAGAGCGGCTCCGGCGGAAGGAGAATGTAG

>Line07_FT2-C_protein

MLKSMARARDQEPLVIGRVIGDVIEPFTKSVSLRMTYSNNREVTSGCELKPSHVINRPRVQIGG

DDLRIFYTLVMVDPDAPSPSDPNLKEYLHWLVTDIPATTGASFARYYVNAPT**QQAKRF***

>Line32_FT2-C_DNA

ATGCTTAAATCTATGGCGAGGGCTAGAGATCAGGAGCCTCTTGTCATCGGGAGAGTGATCGGAGATGTTATAGAGCCGTTCACGAAGTCTGTGTCTTTGAGGATGACTTACAGTAATAACAGGGAGGTTACCAGTGGCTGTGAGCTTAAACCTTCTCATGTTATCAACCGACCTCGAGTTCAGATAGGTGGAGATGATCTTAGGATCTTCTACACTCTGgtaagcaattacttgcatacatatacccatctgtatattgcatgcatatatcatgctctttgtcttgtttgtttagaaatgcacgagtttagcctacagcttccatttatacttgattgcagGTCATGGTAGATCCTGATGCGCCCAGTCCAAGTGATCCCAACCTCAAGGAATACTTGCATTGgtaatattgctctgttttccctctttttagttatcaaacgcacaggagagagcaaaagcatgagctataagccctgatacgtagtattattgcatgctaagctttttgacgtttcgaattccatcttcaagtcaagatggcaagaactctaaggaaacgatcttgctagaccaagaaggatatgtgtaaaggagaagaacatgcatccgttcattttgattgattaattttctttttatttcagaattagatatgtctacgctaggttgatgatgattgaggtattcatcctttacatgttttttcagGTTGGTTACTGATATTCCGGCAACAACTGGAGCAAGCTTCGgtgagtacttatacatttcatccactttgttgaatttgggttacttctaataccagCAAGATACTAT**GTAAACGCACCAAC‑‑‑TGCAGG**CCAAGAGATTTTGAGCTATGAAAGTCCAAGGCCATCTATGGGGATCCATCGCTTTGTTTTGGTTTTGTTTCACCAATTGGGAAGGCAAACAGTGTATGCTCCAGGATGGCGCCAAAATTTTAACACCAGAGAGTTTGCTGAGAACTGTAATCTTGGATCACCGGTGGCCGCTGTCTTTTGTAACTGCCAGAGGGAGAGCGGCTCCGGCGGAAGGAGAATGTAG

>Line32_FT2-C_protein

MLKSMARARDQEPLVIGRVIGDVIEPFTKSVSLRMTYSNNREVTSGCELKPSHVINRPRVQIGGDDLRIFYTLVMVDPDAPSPSDPNLKEYLHWLVTDIPATTGASFARYYVNAPT‑AGQEILSYESPRPSMGIHRFVLVLFHQLGRQTVYAPGWRQNFNTREFAENCNLGSPVAAVFCNCQRESGSGGRRM*

>LineXXXX_FT2-D_DNA

ATGCTTAAATCTATGGCGAGGGCTAGAGATCAGGAGCCTCTTGTCGTCGGGAGAGTGATCGGAGATGTTATAGAGCCGTTCACGAAGTCTGTGTCTTTGAGGATGACTTACAGTAATAACAGGGAGGTTACCAGTGGCTGTGAGCTTAAACCTTCTCATGTTATCAACCGACCTCGAGTTCAGATAGGTGGAGATGATCTTAGGAACTTCTACACTCTGgtaagcaattacttgcatacatatacccatctgtatatcgcatgcatatatcatgctctttgtcttgtttgtttagaaatgcatgagtttagcgtacagcttccatttatacttgattgcagGTCATGGTAGATCCTGATGCGCCCAGTCCAAGTGATCCCAACCTCAAGGAATACTTGCATTGgtaatattgctctgttttccctctttttagttatcaaacgcataggagagagcaaaagcatgagctataagccctgatacgtagtattattgcatgctatgctttttgagatttcgaattccatcttcaagtcaagatggcaagaactctaaggaaacgatctagctagcccaagaaggatatgtgtaaaggagaagaacatgcatccgttcattttgattgattaattttctttttatttcagaattagatgtctacactaggttgatgatgattgaggtattcatcctttacatgttttttcagGTTGGTTACTGATATTCCGGCAACAACTGGAGCAAGCTTCGgtgagtacttatacatttcatccactttgttgaattgggttacttctaataccagcaagatactatgtaaacgcaccatttcatgcaggccaagagattgtgagctatgaaagtccaaGGCCATCTATGGGGATCCATCGCTTTGTTTCGGTTTTGTTTCGCCAATTGGGAAGGCAAACAGTGTATGCTCCAGGATGGCGCCAAAATTTTAACACCAGAGAGTTTGCTGAGAACTATAATCTTGGATCACCGGTGGCCGCTGTCTATTTTAACTGCCAGAGGGAGAGCGGCTCCGGCGGAAGGAGAATGTAG

>LineXXXX_FT2-D_protein

MLKSMARARDQEPLVVGRVIGDVIEPFTKSVSLRMTYSNNREVTSGCELKPSHVINRPRVQIGGDDLRNFYTLVMVDPDAPSPSDPNLKEYLHWLVTDIPATTGASFGPSMGIHRFVSVLFRQLGRQTVYAPGWRQNFNTREFAENYNLGSPVAAVYFNCQRESGSGGRRM*

>Line09_FT2-D_DNA

ATGCTTAAATCTATGGCGAGGGCTAGAGATCAGGAGCCTCTTGTCGTCGGGAGAGTGATCGGAGATGTTATAGAGCCGTTCACGAAGTCTGTGTCTTTGAGGATGACTTACAGT**‑‑‑‑‑‑**AGGGAGGTTACCAGTGGCTGTGAGCTTAAACCTTCTCATGTTATCAACCGACCTCGAGTTCAGATAGGTGGAGATGATCTTAGGAACTTCTACACTCTGgtaagcaattacttgcatacatatacccatctgtatatcgcatgcatatatcatgctctttgtcttgtttgtttagaaatgcatgagtttagcgtacagcttccatttatacttgattgcagGTCATGGTAGATCCTGATGCGCCCAGTCCAAGTGATCCCAACCTCAAGGAATACTTGCATTGgtaatattgctctgttttccctctttttagttatcaaacgcataggagagagcaaaagcatgagctataagccctgatacgtagtattattgcatgctatgctttttgagatttcgaattccatcttcaagtcaagatggcaagaactctaaggaaacgatctagctagcccaagaaggatatgtgtaaaggagaagaacatgcatccgttcattttgattgattaattttctttttatttcagaattagatgtctacactaggttgatgatgattgaggtattcatcctttacatgttttttcagGTTGGTTACTGATATTCCGGCAACAACTGGAGCAAGCTTCGgtgagtacttatacatttcatccactttgttgaattgggttacttctaataccagcaagatactatgtaaacgcaccatttcatgcaggccaagagattgtgagctatgaaagtccaaGGCCATCTATGGGGATCCATCGCTTTGTTTCGGTTTTGTTTCGCCAATTGGGAAGGCAAACAGTGTATGCTCCAGGATGGCGCCAAAATTTTAACACCAGAGAGTTTGCTGAGAACTATAATCTTGGATCACCGGTGGCCGCTGTCTATTTTAACTGCCAGAGGGAGAGCGGCTCCGGCGGAAGGAGAATGTAG

>Line09_FT2-D_protein

MLKSMARARDQEPLVVGRVIGDVIEPFTKSVSLRMTYS**‑‑**REVTSGCELKPSHVINRPRVQIGGDDLRNFYTLVMVDPDAPSPSDPNLKEYLHWLVTDIPATTGASFGPSMGIHRFVSVLFRQLGRQTVYAPGWRQNFNTREFAENYNLGSPVAAVYFNCQRESGSGGRRM*

>Line34_FT2-D_DNA

ATGCTTAAATCTATGGCGAGGGCTAGAGATCAGGAGCCTCTTGTCGTCGGGAGAGTGATCGGAGATGTTATAGAGCCGTTCACGAAGTCTGTGTCTTTGAGGATGACTTACAGTAATAAC**A**AGGGAGGTTACCAGTGGCTGTGAGCTTAAACCTTCTCATGTTATCAACCGACCTCGAGTTCAGATAGGTGGAGATGATCTTAGGAACTTCTACACTCTGgtaagcaattacttgcatacatatacccatctgtatatcgcatgcatatatcatgctctttgtcttgtttgtttagaaatgcatgagtttagcgtacagcttccatttatacttgattgcagGTCATGGTAGATCCTGATGCGCCCAGTCCAAGTGATCCCAACCTCAAGGAATACTTGCATTGgtaatattgctctgttttccctctttttagttatcaaacgcataggagagagcaaaagcatgagctataagccctgatacgtagtattattgcatgctatgctttttgagatttcgaattccatcttcaagtcaagatggcaagaactctaaggaaacgatctagctagcccaagaaggatatgtgtaaaggagaagaacatgcatccgttcattttgattgattaattttctttttatttcagaattagatgtctacactaggttgatgatgattgaggtattcatcctttacatgttttttcagGTTGGTTACTGATATTCCGGCAACAACTGGAGCAAGCTTCGgtgagtacttatacatttcatccactttgttgaattgggttacttctaataccagcaagatactatgtaaacgcaccatttcatgcaggccaagagattgtgagctatgaaagtccaaGGCCATCTATGGGGATCCATCGCTTTGTTTCGGTTTTGTTTCGCCAATTGGGAAGGCAAACAGTGTATGCTCCAGGATGGCGCCAAAATTTTAACACCAGAGAGTTTGCTGAGAACTATAATCTTGGATCACCGGTGGCCGCTGTCTATTTTAACTGCCAGAGGGAGAGCGGCTCCGGCGGAAGGAGAATGTAG

>Line34_FT2-D_protein

MLKSMARARDQEPLVVGRVIGDVIEPFTKSVSLRMTYSNN**KGGYQWL***

>LineXXXX_TFL1-A_DNA

ATGGCAAGAATGTCGGAACCTCTAGCTGTTGGAAGAGTCATAGGAGATGTTCTTGATTCCTTCACCCCCACTACAAAAATGATTGTCTCTTACAACAGCAAGCTCGTCTGCAATGGACATGAGCTCTTCCCTTCTGCAGTCACCGCCAAACCTAGAGTTGAGATTCAAGGAGGCGA**CATGAGATCATTCTTCACTCTGg**tatacatcattttctttctggcctcctttttccgccttttgttttatatcagatctaccagatcgctaactttagttattttcacagGTAATGACAGACCCAGATGTTCCTGGCCCTAGTGATCCTT**ATTTGAAAGAGCACCTGCACTGg**tatatatcttacagttaatactgtatattatcaaacctagaaacgaagaaacaaagacaataacaattcagataattaataacttcaatgttcaaactagatatatatgttgtcatattctagaaatttgcagtattgaccaaacaaattgtccaggttaatttatagatcaattgcactcctgcttgtaatttccagattcaaagaaaagattagttatattaattattgttaatttattctttctcctttacatggtcattcacgtatatattcagaattgtaatcagaaaatctttggatctggaaataaaagcaagtaaaaattacatattatgtcacgaactcttaactagaaaaactagcaaggtataattgtggagtatatacatgccaataagcttggatctaacagttttgccatgaaattgaactatacagGATTGTGACAGACATTCCTGGCACCACAGATGCAACATTTGgtaggttaattcaaactagttcatgagctaccagtacgttatacagctagtgatgatcagcactgatattgtgtatatactacattaattaacagGAAGAGAAGTGGTGAGCTACGAGATGCCAAGGCCAAACATAGGCATCCACAGGTTTGTGTTTGTTCTCTTCATGCAAAAACGAAGGCAGTCGGTGAACCCGCCTTCCTCAAGGGATCACTTCAACACCCGAACCTTTGCAGCCGAAAACGACCTTGGTGTTCCTGTTGCTGCCGTTTACTTCAATGCACAGAGAGAAACGGCAGCAAGAAGACGCTAG

>LineXXXX_TFL1-A_protein

MARMSEPLAVGRVIGDVLDSFTPTTKMIVSYNSKLVCNGHELFPSAVTAKPRVEIQGGDMRSFF

TLVMTDPDVPGPSDPYLKEHLHWIVTDIPGTTDATFGREVVSYEMPRPNIGIHRFVFVLFMQKRRQSVNPPSSRDHFNTRTFAAENDLGVPVAAVYFNAQRETAARRR*

>LineC20_TFL1-A_DNA_hap1

ATGGCAAGAATGTCGGAACCTCTAGCTGTTGGAAGAGTCATAGGAGATGTTCTTGATTCCTTCACCCCCACTACAAAAATGATTGTCTCTTACAACAGCAAGCTCGTCTGCAATGGACATGAGCTCTTCCCTTCTGCAGTCACCGCCAAACCTAGAGTTGAGATTCAAGGAGGCGA**CATGAGATCATTCTTC‑CTCTGg**tatacatcattttctttctggcctcctttttccgccttttgttttatatcagatctaccagatcgctaactttagttattttcacagGTAATGACAGACCCAGATGTTCCTGGCCCTAGTGATCCTT**ATTTGAAAGAGCACC‑‑‑ACTGg**tatatatcttacagttaatactgtatattatcaaacctagaaacgaagaaacaaagacaataacaattcagataattaataacttcaatgttcaaactagatatatatgttgtcatattctagaaatttgcagtattgaccaaacaaattgtccaggttaatttatagatcaattgcactcctgcttgtaatttccagattcaaagaaaagattagttatattaattattgttaatttattctttctcctttacatggtcattcacgtatatattcagaattgtaatcagaaaatctttggatctggaaataaaagcaagtaaaaattacatattatgtcacgaactcttaactagaaaaactagcaaggtataattgtggagtatatacatgccaataagcttggatctaacagttttgccatgaaattgaactatacagGATTGTGACAGACATTCCTGGCACCACAGATGCAACATTTGgtaggttaattcaaactagttcatgagctaccagtacgttatacagctagtgatgatcagcactgatattgtgtatatactacattaattaacagGAAGAGAAGTGGTGAGCTACGAGATGCCAAGGCCAAACATAGGCATCCACAGGTTTGTGTTTGTTCTCTTCATGCAAAAACGAAGGCAGTCGGTGAACCCGCCTTCCTCAAGGGATCACTTCAACACCCGAACCTTTGCAGCCGAAAACGACCTTGGTGTTCCTGTTGCTGCCGTTTACTTCAATGCACAGAGAGAAACGGCAGCAAGAAGACGCTAG

>LineC20_TFL1-A_protein_hap1

MARMSEPLAVGRVIGDVLDSFTPTTKMIVSYNSKLVCNGHELFPSAVTAKPRVEIQGGDMRSFF**LW***

>LineC20_TFL1-A_DNA_hap2

ATGGCAAGAATGTCGGAACCTCTAGCTGTTGGAAGAGTCATAGGAGATGTTCTTGATTCCTTCACCCCCACTACAAAAATGATTGTCTCTTACAACAGCAAGCTCGTCTGCAATGGACATGAGCTCTTCCCTTCTGCAGTCACCGCCAAACCTAGAGTTGAGATTCAAGGAGGCGA**CATGAGATCATTCTTCACTCTGg**tatacatcattttctttctggcctcctttttccgccttttgttttatatcagatctaccagatcgctaactttagttattttcacagGTAATGACAGACCCAGATGTTCCTGGCCCTAGTGATCCTT**ATTTGAAAGAGC‑‑‑‑‑CACTGg**tatatatcttacagttaatactgtatattatcaaacctagaaacgaagaaacaaagacaataacaattcagataattaataacttcaatgttcaaactagatatatatgttgtcatattctagaaatttgcagtattgaccaaacaaattgtccaggttaatttatagatcaattgcactcctgcttgtaatttccagattcaaagaaaagattagttatattaattattgttaatttattctttctcctttacatggtcattcacgtatatattcagaattgtaatcagaaaatctttggatctggaaataaaagcaagtaaaaattacatattatgtcacgaactcttaactagaaaaactagcaaggtataattgtggagtatatacatgccaataagcttggatctaacagttttgccatgaaattgaactatacagGATTGTGACAGACATTCCTGGCACCACAGATGCAACATTTGgtaggttaattcaaactagttcatgagctaccagtacgttatacagctagtgatgatcagcactgatattgtgtatatactacattaattaacagGAAGAGAAGTGGTGAGCTACGAGATGCCAAGGCCAAACATAGGCATCCACAGGTTTGTGTTTGTTCTCTTCATGCAAAAACGAAGGCAGTCGGTGAACCCGCCTTCCTCAAGGGATCACTTCAACACCCGAACCTTTGCAGCCGAAAACGACCTTGGTGTTCCTGTTGCTGCCGTTTACTTCAATGCACAGAGAGAAACGGCAGCAAGAAGACGCTAG

>LineC20_TFL1-A_protein_hap2

MARMSEPLAVGRVIGDVLDSFTPTTKMIVSYNSKLVCNGHELFPSAVTAKPRVEIQGGDMRSFFTLVMTDPDVPGPSDPYLKE**PLDCDRHSWHHRCNIW***

>LineC24_TFL1-A_DNA_hap1

ATGGCAAGAATGTCGGAACCTCTAGCTGTTGGAAGAGTCATAGGAGATGTTCTTGATTCCTTCACCCCCACTACAAAAATGATTGTCTCTTACAACAGCAAGCTCGTCTGCAATGGACATGAGCTCTTCCCTTCTGCAGTCACCGCCAAACCTAGAGTTGAGATTCAAGGAGGCGACATGAGATCATTCTTCACTCTGgtatacatcattttctttctggcctcctttttccgccttttgttttatatcagatctaccagatcgctaactttagttattttcacagGTAATGACAGACCCAGATGTTCCTGGCCCTAGTGATCCTT**ATTTGAAAGAGCACCTGCCACTGg**tatatatcttacagttaatactgtatattatcaaacctagaaacgaagaaacaaagacaataacaattcagataattaataacttcaatgttcaaactagatatatatgttgtcatattctagaaatttgcagtattgaccaaacaaattgtccaggttaatttatagatcaattgcactcctgcttgtaatttccagattcaaagaaaagattagttatattaattattgttaatttattctttctcctttacatggtcattcacgtatatattcagaattgtaatcagaaaatctttggatctggaaataaaagcaagtaaaaattacatattatgtcacgaactcttaactagaaaaactagcaaggtataattgtggagtatatacatgccaataagcttggatctaacagttttgccatgaaattgaactatacagGATTGTGACAGACATTCCTGGCACCACAGATGCAACATTTGgtagttaattcaaactagttcatgagctaccagtacgttatacagctagtgatgatcagcactgatattgtgtatatactacattaattaacagGAAGAGAAGTGGTGAGCTACGAGATGCCAAGGCCAAACATAGGCATCCACAGGTTTGTGTTTGTTCTCTTCATGCAAAAACGAAGGCAGTCGGTGAACCCGCCTTCCTCAAGGGATCACTTCAACACCCGAACCTTTGCAGCCGAAAACGACCTTGGTGTTCCTGTTGCTGCCGTTTACTTCAATGCACAGAGAGAAACGGCAGCAAGAAGACGCTAG

>LineC24_TFL1-A_protein_hap1

MARMSEPLAVGRVIGDVLDSFTPTTKMIVSYNSKLVCNGHELFPSAVTAKPRVEIQGGDMRSFFTLVMTDPDVPGPSDPYLKEHL**PLDCDRHSWHHRCNIW***

>LineC24_TFL1-A_DNA_hap2

ATGGCAAGAATGTCGGAACCTCTAGCTGTTGGAAGAGTCATAGGAGATGTTCTTGATTCCTTCACCCCCACTACAAAAATGATTGTCTCTTACAACAGCAAGCTCGTCTGCAATGGACATGAGCTCTTCCCTTCTGCAGTCACCGCCAAACCTAGAGTTGAGATTCAAGGAGGCGACATGAGATCATTCTTCACTCTGgtatacatcattttctttctggcctcctttttccgccttttgttttatatcagatctaccagatcgctaactttagttattttcacagGTAATGACAGACCCAGATGTTCCTGGCCCTAGTGATCCTT**ATTTGAAAGAGCACC‑‑‑ACTGg**tatatatcttacagttaatactgtatattatcaaacctagaaacgaagaaacaaagacaataacaattcagataattaataacttcaatgttcaaactagatatatatgttgtcatattctagaaatttgcagtattgaccaaacaaattgtccaggttaatttatagatcaattgcactcctgcttgtaatttccagattcaaagaaaagattagttatattaattattgttaatttattctttctcctttacatggtcattcacgtatatattcagaattgtaatcagaaaatctttggatctggaaataaaagcaagtaaaaattacatattatgtcacgaactcttaactagaaaaactagcaaggtataattgtggagtatatacatgccaataagcttggatctaacagttttgccatgaaattgaactatacagGATTGTGACAGACATTCCTGGCACCACAGATGCAACATTTGgtaggttaattcaaactagttcatgagctaccagtacgttatacagctagtgatgatcagcactgatattgtgtatatactacattaattaacagGAAGAGAAGTGGTGAGCTACGAGATGCCAAGGCCAAACATAGGCATCCACAGGTTTGTGTTTGTTCTCTTCATGCAAAAACGAAGGCAGTCGGTGAACCCGCCTTCCTCAAGGGATCACTTCAACACCCGAACCTTTGCAGCCGAAAACGACCTTGGTGTTCCTGTTGCTGCCGTTTACTTCAATGCACAGAGAGAAACGGCAGCAAGAAGACGCTAG

>LineC24_TFL1-A_protein_hap2

MARMSEPLAVGRVIGDVLDSFTPTTKMIVSYNSKLVCNGHELFPSAVTAKPRVEIQGGDMRSFFTLVMTDPDVPGPSDPYLKEH**‑**HWIVTDIPGTTDATFGREVVSYEMPRPNIGIHRFVFVLFMQKRRQSVNPPSSRDHFNTRTFAAENDLGVPVAAVYFNAQRETAARRR*

>LineC22_TFL1-A_DNA_hap1-2

ATGGCAAGAATGTCGGAACCTCTAGCTGTTGGAAGAGTCATAGGAGATGTTCTTGATTCCTTCACCCCCACTACAAAAATGATTGTCTCTTACAACAGCAAGCTCGTCTGCAATGGACATGAGCTCTTCCCTTCTGCAGTCACCGCCAAACCTAGAGTTGAGATTCAAGGAGGCGACATGAGATCATTCTTCACTCTGgtatacatcattttctttctggcctcctttttccgccttttgttttatatcagatctaccagatcgctaactttagttattttcacagGTAATGACAGACCCAGATGTTCCTGGCCCTAGTGATCCTT**ATTTGAAAGAGCACCTG‑‑‑‑‑g**tatatatcttacagttaatactgtatattatcaaacctagaaacgaagaaacaaagacaataacaattcagataattaataacttcaatgttcaaactagatatatatgttgtcatattctagaaatttgcagtattgaccaaacaaattgtccaggttaatttatagatcaattgcactcctgcttgtaatttccagattcaaagaaaagattagttatattaattattgttaatttattctttctcctttacatggtcattcacgtatatattcagaattgtaatcagaaaatctttggatctggaaataaaagcaagtaaaaattacatattatgtcacgaactcttaactagaaaaactagcaaggtataattgtggagtatatacatgccaataagcttggatctaacagttttgccatgaaattgaactatacagGATTGTGACAGACATTCCTGGCACCACAGATGCAACATTTGgtaggttaattcaaactagttcatgagctaccagtacgttatacagctagtgatgatcagcactgatattgtgtatatactacattaattaacagGAAGAGAAGTGGTGAGCTACGAGATGCCAAGGCCAAACATAGGCATCCACAGGTTTGTGTTTGTTCTCTTCATGCAAAAACGAAGGCAGTCGGTGAACCCGCCTTCCTCAAGGGATCACTTCAACACCCGAACCTTTGCAGCCGAAAACGACCTTGGTGTTCCTGTTGCTGCCGTTTACTTCAATGCACAGAGAGAAACGGCAGCAAGAAGACGCTAG

>LineC22_TFL1-A_protein_hap1-2

MARMSEPLAVGRVIGDVLDSFTPTTKMIVSYNSKLVCNGHELFPSAVTAKPRVEIQGGDMRSFFTLVMTDPDVPGPSDPYLKEHL**DCDRHSWHHRCNIW***

>LineC21_TFL1-A_DNA_hap1

ATGGCAAGAATGTCGGAACCTCTAGCTGTTGGAAGAGTCATAGGAGATGTTCTTGATTCCTTCACCCCCACTACAAAAATGATTGTCTCTTACAACAGCAAGCTCGTCTGCAATGGACATGAGCTCTTCCCTTCTGCAGTCACCGCCAAACCTAGAGTTGAGATTCAAGGAGGCGACATGAGATCATTCTTCACTCTGgtatacatcattttctttctggcctcctttttccgccttttgttttatatcagatctaccagatcgctaactttagttattttcacagGTAATGACAGACCCAGATGTTCCTGGCCCTAGTGATCCTT**ATTTGAAAGAGCAC‑‑‑‑ACTGg**tatatatcttacagttaatactgtatattatcaaacctagaaacgaagaaacaaagacaataacaattcagataattaataacttcaatgttcaaactagatatatatgttgtcatattctagaaatttgcagtattgaccaaacaaattgtccaggttaatttatagatcaattgcactcctgcttgtaatttccagattcaaagaaaagattagttatattaattattgttaatttattctttctcctttacatggtcattcacgtatatattcagaattgtaatcagaaaatctttggatctggaaataaaagcaagtaaaaattacatattatgtcacgaactcttaactagaaaaactagcaaggtataattgtggagtatatacatgccaataagcttggatctaacagttttgccatgaaattgaactatacagGATTGTGACAGACATTCCTGGCACCACAGATGCAACATTTGgtaggttaattcaaactagttcatgagctaccagtacgttatacagctagtgatgatcagcactgatattgtgtatatactacattaattaacagGAAGAGAAGTGGTGAGCTACGAGATGCCAAGGCCAAACATAGGCATCCACAGGTTTGTGTTTGTTCTCTTCATGCAAAAACGAAGGCAGTCGGTGAACCCGCCTTCCTCAAGGGATCACTTCAACACCCGAACCTTTGCAGCCGAAAACGACCTTGGTGTTCCTGTTGCTGCCGTTTACTTCAATGCACAGAGAGAAACGGCAGCAAGAAGACGCTAG

>LineC21_TFL1-A_protein_hap1

MARMSEPLAVGRVIGDVLDSFTPTTKMIVSYNSKLVCNGHELFPSAVTAKPRVEIQGGDMRSFFTLVMTDPDVPGPSDPYLKEH**TGL***

> LineC21_TFL1-A_DNA_hap2

ATGGCAAGAATGTCGGAACCTCTAGCTGTTGGAAGAGTCATAGGAGATGTTCTTGATTCCTTCACCCCCACTACAAAAATGATTGTCTCTTACAACAGCAAGCTCGTCTGCAATGGACATGAGCTCTTCCCTTCTGCAGTCACCGCCAAACCTAGAGTTGAGATTCAAGGAGGCGA**CATGAGATCATTCTTCACTCTGg**tatacatcattttctttctggcctcctttttccgccttttgttttatatcagatctaccagatcgctaactttagttattttcacagGTAATGACAGACCCAGATGTTCCTGGCCCTAGTGATCCTT**ATTTGAAAGAGCACCTG‑‑‑‑‑‑**tatatatcttacagttaatactgtatattatcaaacctagaaacgaagaaacaaagacaataacaattcagataattaataacttcaatgttcaaactagatatatatgttgtcatattctagaaatttgcagtattgaccaaacaaattgtccaggttaatttatagatcaattgcactcctgcttgtaatttccagattcaaagaaaagattagttatattaattattgttaatttattctttctcctttacatggtcattcacgtatatattcagaattgtaatcagaaaatctttggatctggaaataaaagcaagtaaaaattacatattatgtcacgaactcttaactagaaaaactagcaaggtataattgtggagtatatacatgccaataagcttggatctaacagttttgccatgaaattgaactatacagGATTGTGACAGACATTCCTGGCACCACAGATGCAACATTTGgtaggttaattcaaactagttcatgagctaccagtacgttatacagctagtgatgatcagcactgatattgtgtatatactacattaattaacagGAAGAGAAGTGGTGAGCTACGAGATGCCAAGGCCAAACATAGGCATCCACAGGTTTGTGTTTGTTCTCTTCATGCAAAAACGAAGGCAGTCGGTGAACCCGCCTTCCTCAAGGGATCACTTCAACACCCGAACCTTTGCAGCCGAAAACGACCTTGGTGTTCCTGTTGCTGCCGTTTACTTCAATGCACAGAGAGAAACGGCAGCAAGAAGACGCTAG

> LineC21_TFL1-A_protein_hap2

MARMSEPLAVGRVIGDVLDSFTPTTKMIVSYNSKLVCNGHELFPSAVTAKPRVEIQGGDMRSFFTLVMTDPDVPGPSDPYLKEHL**YISYS***

>LineC19_TFL1-A_DNA_hap1-2

ATGGCAAGAATGTCGGAACCTCTAGCTGTTGGAAGAGTCATAGGAGATGTTCTTGATTCCTTCACCCCCACTACAAAAATGATTGTCTCTTACAACAGCAAGCTCGTCTGCAATGGACATGAGCTCTTCCCTTCTGCAGTCACCGCCAAACCTAGAGTTGAGATTCAAGGAGGCGACATGAGATCATTCTTCACTCTGgtatacatcattttctttctggcctcctttttccgccttttgttttatatcagatctaccagatcgctaactttagttattttcacagGTAATGACAGACCCAGATGTTCCTGGCCCTAGTGATCCTT**ATTTGAAAGAGCACCT‑‑‑‑‑‑‑‑**atatatcttacagttaatactgtatattatcaaacctagaaacgaagaaacaaagacaataacaattcagataattaataacttcaatgttcaaactagatatatatgttgtcatattctagaaatttgcagtattgaccaaacaaattgtccaggttaatttatagatcaattgcactcctgcttgtaatttccagattcaaagaaaagattagttatattaattattgttaatttattctttctcctttacatggtcattcacgtatatattcagaattgtaatcagaaaatctttggatctggaaataaaagcaagtaaaaattacatattatgtcacgaactcttaactagaaaaactagcaaggtataattgtggagtatatacatgccaataagcttggatctaacagttttgccatgaaattgaactatacagGATTGTGACAGACATTCCTGGCACCACAGATGCAACATTTGgtaggttaattcaaactagttcatgagctaccagtacgttatacagctagtgatgatcagcactgatattgtgtatatactacattaattaacagGAAGAGAAGTGGTGAGCTACGAGATGCCAAGGCCAAACATAGGCATCCACAGGTTTGTGTTTGTTCTCTTCATGCAAAAACGAAGGCAGTCGGTGAACCCGCCTTCCTCAAGGGATCACTTCAACACCCGAACCTTTGCAGCCGAAAACGACCTTGGTGTTCCTGTTGCTGCCGTTTACTTCAATGCACAGAGAGAAACGGCAGCAAGAAGACGCTAG

>LineC19_TFL1-A_protein

MARMSEPLAVGRVIGDVLDSFTPTTKMIVSYNSKLVCNGHELFPSAVTAKPRVEIQGGDMRSFFTLVMTDPDVPGPSDPYLKEHL**YILQLILYIIKPRNEETKTITIQIINNFNVQTRYICCHILEICSIDQTNCPG***

>Line14_TFL1-A_DNA_hap1

ATGGCAAGAATGTCGGAACCTCTAGCTGTTGGAAGAGTCATAGGAGATGTTCTTGATTCCTTCACCCCCACTACAAAAATGATTGTCTCTTACAACAGCAAGCTCGTCTGCAATGGACATGAGCTCTTCCCTTCTGCAGTCACCGCCAAACCTAGAGTTGAGATTCAAGGAGGCGA**CATGAGATC‑‑‑‑‑‑‑‑‑‑‑‑‑g**tatacatcattttctttctggcctcctttttccgccttttgttttatatcagatctaccagatcgctaactttagttattttcacagGTAATGACAGACCCAGATGTTCCTGGCCCTAGTGATCCTT**ATTTGAAAGAGCACCTGACACTGg**tatatatcttacagttaatactgtatattatcaaacctagaaacgaagaaacaaagacaataacaattcagataattaataacttcaatgttcaaactagatatatatgttgtcatattctagaaatttgcagtattgaccaaacaaattgtccaggttaatttatagatcaattgcactcctgcttgtaatttccagattcaaagaaaagattagttatattaattattgttaatttattctttctcctttacatggtcattcacgtatatattcagaattgtaatcagaaaatctttggatctggaaataaaagcaagtaaaaattacatattatgtcacgaactcttaactagaaaaactagcaaggtataattgtggagtatatacatgccaataagcttggatctaacagttttgccatgaaattgaactatacagGATTGTGACAGACATTCCTGGCACCACAGATGCAACATTTGgtaggttaattcaaactagttcatgagctaccagtacgttatacagctagtgatgatcagcactgatattgtgtatatactacattaattaacagGAAGAGAAGTGGTGAGCTACGAGATGCCAAGGCCAAACATAGGCATCCACAGGTTTGTGTTTGTTCTCTTCATGCAAAAACGAAGGCAGTCGGTGAACCCGCCTTCCTCAAGGGATCACTTCAACACCCGAACCTTTGCAGCCGAAAACGACCTTGGTGTTCCTGTTGCTGCCGTTTACTTCAATGCACAGAGAGAAACGGCAGCAAGAAGACGCTAG

>Line14_TFL1-A_protein_hap1

MARMSEPLAVGRVIGDVLDSFTPTTKMIVSYNSKLVCNGHELFPSAVTAKPRVEIQGGDMRS*****

>Line14_TFL1-A_DNA_hap2

ATGGCAAGAATGTCGGAACCTCTAGCTGTTGGAAGAGTCATAGGAGATGTTCTTGATTCCTTCACCCCCACTACAAAAATGATTGTCTCTTACAACAGCAAGCTCGTCTGCAATGGACATGAGCTCTTCCCTTCTGCAGTCACCGCCAAACCTAGAGTTGAGATTCAAGGAGGCGA**CATGAGATCATTCTTCACTCTGg**tatacatcattttctttctggcctcctttttccgccttttgttttatatcagatctaccagatcgctaactttagttattttcacagGTAATGACAGACCCAGATGTTCCTGGCCCTAGTGATCCTT**ATTTGAAAGAGCACCTGC‑‑TGg**tatatatcttacagttaatactgtatattatcaaacctagaaacgaagaaacaaagacaataacaattcagataattaataacttcaatgttcaaactagatatatatgttgtcatattctagaaatttgcagtattgaccaaacaaattgtccaggttaatttatagatcaattgcactcctgcttgtaatttccagattcaaagaaaagattagttatattaattattgttaatttattctttctcctttacatggtcattcacgtatatattcagaattgtaatcagaaaatctttggatctggaaataaaagcaagtaaaaattacatattatgtcacgaactcttaactagaaaaactagcaaggtataattgtggagtatatacatgccaataagcttggatctaacagttttgccatgaaattgaactatacagGATTGTGACAGACATTCCTGGCACCACAGATGCAACATTTGgtaggttaattcaaactagttcatgagctaccagtacgttatacagctagtgatgatcagcactgatattgtgtatatactacattaattaacagGAAGAGAAGTGGTGAGCTACGAGATGCCAAGGCCAAACATAGGCATCCACAGGTTTGTGTTTGTTCTCTTCATGCAAAAACGAAGGCAGTCGGTGAACCCGCCTTCCTCAAGGGATCACTTCAACACCCGAACCTTTGCAGCCGAAAACGACCTTGGTGTTCCTGTTGCTGCCGTTTACTTCAATGCACAGAGAGAAACGGCAGCAAGAAGACGCTAG

>Line14_TFL1-A_protein_hap2

MARMSEPLAVGRVIGDVLDSFTPTTKMIVSYNSKLVCNGHELFPSAVTAKPRVEIQGGDMRSFFTLVMTDPDVPGPSDPYLKEHL**LDCDRHSWHHRCNIW***

>Line15_TFL1-A_DNA_hap1

ATGGCAAGAATGTCGGAACCTCTAGCTGTTGGAAGAGTCATAGGAGATGTTCTTGATTCCTTCACCCCCACTACAAAAATGATTGTCTCTTACAACAGCAAGCTCGTCTGCAATGGACATGAGCTCTTCCCTTCTGCAGTCACCGCCAAACCTAGAGTTGAGATTCAAGGAGGCGACATGAGATCATTCTTCACTCTGgtatacatcattttctttctggcctcctttttccgccttttgttttatatcagatctaccagatcgctaactttagttattttcacagGTAATGACAGACCCAGATGTTCCTGGCCCTAGTGATCCTT**ATTTGAAAGAGCACCTG‑‑‑‑‑g**tatatatcttacagttaatactgtatattatcaaacctagaaacgaagaaacaaagacaataacaattcagataattaataacttcaatgttcaaactagatatatatgttgtcatattctagaaatttgcagtattgaccaaacaaattgtccaggttaatttatagatcaattgcactcctgcttgtaatttccagattcaaagaaaagattagttatattaattattgttaatttattctttctcctttacatggtcattcacgtatatattcagaattgtaatcagaaaatctttggatctggaaataaaagcaagtaaaaattacatattatgtcacgaactcttaactagaaaaactagcaaggtataattgtggagtatatacatgccaataagcttggatctaacagttttgccatgaaattgaactatacagGATTGTGACAGACATTCCTGGCACCACAGATGCAACATTTGgtaggttaattcaaactagttcatgagctaccagtacgttatacagctagtgatgatcagcactgatattgtgtatatactacattaattaacagGAAGAGAAGTGGTGAGCTACGAGATGCCAAGGCCAAACATAGGCATCCACAGGTTTGTGTTTGTTCTCTTCATGCAAAAACGAAGGCAGTCGGTGAACCCGCCTTCCTCAAGGGATCACTTCAACACCCGAACCTTTGCAGCCGAAAACGACCTTGGTGTTCCTGTTGCTGCCGTTTACTTCAATGCACAGAGAGAAACGGCAGCAAGAAGACGCTAG

>Line15_TFL1-A_protein_hap1

MARMSEPLAVGRVIGDVLDSFTPTTKMIVSYNSKLVCNGHELFPSAVTAKPRVEIQGGDMRSFFTLVMTDPDVPGPSDPYLKEHL**DCDRHSWHHRCNIW***

>Line15_TFL1-A_DNA_hap2

ATGGCAAGAATGTCGGAACCTCTAGCTGTTGGAAGAGTCATAGGAGATGTTCTTGATTCCTTCACCCCCACTACAAAAATGATTGTCTCTTACAACAGCAAGCTCGTCTGCAATGGACATGAGCTCTTCCCTTCTGCAGTCACCGCCAAACCTAGAGTTGAGATTCAAGGAGGCGA**CATGAGATCATTCTTCACTCTGg**tatacatcattttctttctggcctcctttttccgccttttgttttatatcagatctaccagatcgctaactttagttattttcacagGTAATGACAGACCCAGATGTTCCTGGCCCTAGTGATCCTT**ATTTGAAAGAGCACCTGACACTGg**tatatatcttacagttaatactgtatattatcaaacctagaaacgaagaaacaaagacaataacaattcagataattaataacttcaatgttcaaactagatatatatgttgtcatattctagaaatttgcagtattgaccaaacaaattgtccaggttaatttatagatcaattgcactcctgcttgtaatttccagattcaaagaaaagattagttatattaattattgttaatttattctttctcctttacatggtcattcacgtatatattcagaattgtaatcagaaaatctttggatctggaaataaaagcaagtaaaaattacatattatgtcacgaactcttaactagaaaaactagcaaggtataattgtggagtatatacatgccaataagcttggatctaacagttttgccatgaaattgaactatacagGATTGTGACAGACATTCCTGGCACCACAGATGCAACATTTGgtaggttaattcaaactagttcatgagctaccagtacgttatacagctagtgatgatcagcactgatattgtgtatatactacattaattaacagGAAGAGAAGTGGTGAGCTACGAGATGCCAAGGCCAAACATAGGCATCCACAGGTTTGTGTTTGTTCTCTTCATGCAAAAACGAAGGCAGTCGGTGAACCCGCCTTCCTCAAGGGATCACTTCAACACCCGAACCTTTGCAGCCGAAAACGACCTTGGTGTTCCTGTTGCTGCCGTTTACTTCAATGCACAGAGAGAAACGGCAGCAAGAAGACGCTAG

>Line15_TFL1-A_protein_hap2

MARMSEPLAVGRVIGDVLDSFTPTTKMIVSYNSKLVCNGHELFPSAVTAKPRVEIQGGDMRSFFTLVMTDPDVPGPSDPYLKEHL**TLDCDRHSWHHRCNIW***

>Line38_TFL1-A_DNA_hap1

ATGGCAAGAATGTCGGAACCTCTAGCTGTTGGAAGAGTCATAGGAGATGTTCTTGATTCCTTCACCCCCACTACAAAAATGATTGTCTCTTACAACAGCAAGCTCGTCTGCAATGGACATGAGCTCTTCCCTTCTGCAGTCACCGCCAAACCTAGAGTTGAGATTCAAGGAGGCGA**CATGAGATCATTCTTCACTCTGg**tatacatcattttctttctggcctcctttttccgccttttgttttatatcagatctaccagatcgctaactttagttattttcacagGTAATGACAGACCCAGATGTTCCTGGCCCTAGTGATCCTT**ATTTGAAAGAGCACCTGACACTGg**tatatatcttacagttaatactgtatattatcaaacctagaaacgaagaaacaaagacaataacaattcagataattaataacttcaatgttcaaactagatatatatgttgtcatattctagaaatttgcagtattgaccaaacaaattgtccaggttaatttatagatcaattgcactcctgcttgtaatttccagattcaaagaaaagattagttatattaattattgttaatttattctttctcctttacatggtcattcacgtatatattcagaattgtaatcagaaaatctttggatctggaaataaaagcaagtaaaaattacatattatgtcacgaactcttaactagaaaaactagcaaggtataattgtggagtatatacatgccaataagcttggatctaacagttttgccatgaaattgaactatacagGATTGTGACAGACATTCCTGGCACCACAGATGCAACATTTGgtaggttaattcaaactagttcatgagctaccagtacgttatacagctagtgatgatcagcactgatattgtgtatatactacattaattaacagGAAGAGAAGTGGTGAGCTACGAGATGCCAAGGCCAAACATAGGCATCCACAGGTTTGTGTTTGTTCTCTTCATGCAAAAACGAAGGCAGTCGGTGAACCCGCCTTCCTCAAGGGATCACTTCAACACCCGAACCTTTGCAGCCGAAAACGACCTTGGTGTTCCTGTTGCTGCCGTTTACTTCAATGCACAGAGAGAAACGGCAGCAAGAAGACGCTAG

>Line38_TFL1-A_protein_hap1

MARMSEPLAVGRVIGDVLDSFTPTTKMIVSYNSKLVCNGHELFPSAVTAKPRVEIQGGDMRSFFTLVMTDPDVPGPSDPYLKEHL**TLDCDRHSWHHRCNIW***

>Line38_TFL1-A_DNA_hap2

ATGGCAAGAATGTCGGAACCTCTAGCTGTTGGAAGAGTCATAGGAGATGTTCTTGATTCCTTCACCCCCACTACAAAAATGATTGTCTCTTACAACAGCAAGCTCGTCTGCAATGGACATGAGCTCTTCCCTTCTGCAGTCACCGCCAAACCTAGAGTTGAGATTCAAGGAGGCGA**CATGAGATCATTCTTCACTCTGg**tatacatcattttctttctggcctcctttttccgccttttgttttatatcagatctaccagatcgctaactttagttattttcacagGTAATGACAGACCCAGATGTTCCTGGCCCTAGTGATCCTT**ATTTGAAAGAGCACC‑‑CACTGg**tatatatcttacagttaatactgtatattatcaaacctagaaacgaagaaacaaagacaataacaattcagataattaataacttcaatgttcaaactagatatatatgttgtcatattctagaaatttgcagtattgaccaaacaaattgtccaggttaatttatagatcaattgcactcctgcttgtaatttccagattcaaagaaaagattagttatattaattattgttaatttattctttctcctttacatggtcattcacgtatatattcagaattgtaatcagaaaatctttggatctggaaataaaagcaagtaaaaattacatattatgtcacgaactcttaactagaaaaactagcaaggtataattgtggagtatatacatgccaataagcttggatctaacagttttgccatgaaattgaactatacagGATTGTGACAGACATTCCTGGCACCACAGATGCAACATTTGgtaggttaattcaaactagttcatgagctaccagtacgttatacagctagtgatgatcagcactgatattgtgtatatactacattaattaacagGAAGAGAAGTGGTGAGCTACGAGATGCCAAGGCCAAACATAGGCATCCACAGGTTTGTGTTTGTTCTCTTCATGCAAAAACGAAGGCAGTCGGTGAACCCGCCTTCCTCAAGGGATCACTTCAACACCCGAACCTTTGCAGCCGAAAACGACCTTGGTGTTCCTGTTGCTGCCGTTTACTTCAATGCACAGAGAGAAACGGCAGCAAGAAGACGCTAG

>Line38_TFL1-A_protein_hap2

MARMSEPLAVGRVIGDVLDSFTPTTKMIVSYNSKLVCNGHELFPSAVTAKPRVEIQGGDMRSFFTLVMTDPDVPGPSDPYLKEH**PLDCDRHSWHHRCNIW***

>LineXXXX_TFL1-B_DNA

ATGGCAAGAATGTCGGAACCTCTAGCTGTTGGAAGAGTCATAGGAGATGTTCTTGATTCCTTCACCCCAACTACAAAAATGATTGTCACTTACAACACCAAGCTCGTCTGCAATGGACATGAGCTCTTCCCTTCTGCAGTCACTGCCAAACCTAAAGTTGAGATTCAAGGAGGCGACATGAGATCATTCTTCACTCTGgtattttctttctggcctccttttccgccgtttgtttttttatcagatctaccagatcgctaacttcagttattttcacagGTAATGACAGACCCAGATGTTCCTGGCCCTAGTGATCCTTATTTGAAAGAGCACCTGCACTGgtatatcttacagttaatactgtctatatagtatcaaacctagaaacgaagaaacaaagacaataactattcagataattattaataacttcaatgttcaaactacatatatatgttgtcatattctagaaatttgcagtattgacctaacaaattgtccaggttaatttatagatcaattgcgctcctgcttgtaatttccagattcaaagaaaagattagttatattaattattgttattttattctttctcctttacatggtcattcacgtattcagaattgcaatcagaaaatctttggatctggaaataaaagcaagtaaaaattacatattatgtcacgaactcttaactagaaaaactagcaagttataattgtggagtatgtacatgccaataaacttggatctaacagtttttccttgaaattgaactatacagGATTGTGACAGACATTCCTGGCACCACAGATGCAACATTTGgtaggttaattcacactagttcatgagctaccagtacgttatacagctagtgatgatcagcactgatattttgtatatactacatcaattaacagGAAGAGAAGTGGTGAGCTACGAGATGCCAAGGCCAAACATAGGCATCCACAGGTTTGTGTTTGTTCTCTTCATGCAAAAACGAAGGCAGTCGGTGAACCCGCCTTCCTCAAGGGATCACTTCAACACCCGAACCTTCGCAGCCGAAAACGACCTTGGTATTCCTGTTGCTGCCGTTTACTTCAATGCACAGAGAGAAACGGCAGCAAGAAGACGCTAG >LineXXXX_TFL1-B_protein

MARMSEPLAVGRVIGDVLDSFTPTTKMIVTYNTKLVCNGHELFPSAVTAKPKVEIQGGDMRSFF

TLVMTDPDVPGPSDPYLKEHLHWIVTDIPGTTDATFGREVVSYEMPRPNIGIHRFVFVLFMQKRRQSVNPPSSRDHFNTRTFAAENDLGIPVAAVYFNAQRETAARRR*

>LineC20_TFL1-B_DNA_hap1-2

ATGGCAAGAATGTCGGAACCTCTAGCTGTTGGAAGAGTCATAGGAGATGTTCTTGATTCCTTCACCCCAACTACAAAAATGATTGTCACTTACAACACCAAGCTCGTCTGCAATGGACATGAGCTCTTCCCTTCTGCAGTCACTGCCAAACCTAAAGTTGAGATTCAAGGAGGCGACATGAGATCATTCTTCACTCTGgtattttctttctggcctccttttccgccgtttgtttttttatcagatctaccagatcgctaacttcagttattttcacagGTAATGACAGACCCAGATGTTCCTGGCCCTAG**‑‑‑‑‑‑‑‑‑‑‑‑‑‑‑‑‑‑‑‑‑‑‑‑‑‑‑‑‑‑g**tatatcttacagttaatactgtctatatagtatcaaacctagaaacgaagaaacaaagacaataactattcagataattattaataacttcaatgttcaaactacatatatatgttgtcatattctagaaatttgcagtattgacctaacaaattgtccaggttaatttatagatcaattgcgctcctgcttgtaatttccagattcaaagaaaagattagttatattaattattgttattttattctttctcctttacatggtcattcacgtattcagaattgcaatcagaaaatctttggatctggaaataaaagcaagtaaaaattacatattatgtcacgaactcttaactagaaaaactagcaagttataattgtggagtatgtacatgccaataaacttggatctaacagtttttccttgaaattgaactatacagGATTGTGACAGACATTCCTGGCACCACAGATGCAACATTTGgtaggttaattcacactagttcatgagctaccagtacgttatacagctagtgatgatcagcactgatattttgtatatactacatcaattaacagGAAGAGAAGTGGTGAGCTACGAGATGCCAAGGCCAAACATAGGCATCCACAGGTTTGTGTTTGTTCTCTTCATGCAAAAACGAAGGCAGTCGGTGAACCCGCCTTCCTCAAGGGATCACTTCAACACCCGAACCTTCGCAGCCGAAAACGACCTTGGTATTCCTGTTGCTGCCGTTTACTTCAATGCACAGAGAGAAACGGCAGCAAGAAGACGCTAG

>LineC20_TFL1-B_protein

MARMSEPLAVGRVIGDVLDSFTPTTKMIVTYNTKLVCNGHELFPSAVTAKPKVEIQGGDMRSFF

TLVMTDPDVPGP**‑‑‑‑‑‑‑‑‑‑**RIVTDIPGTTDATFGREVVSYEMPRPNIGIHRFVFVLFMQKRRQSVNPPSSRDHFNTRTFAAENDLGIPVAAVYFNAQRETAARRR*

>LineC24_TFL1-B_DNA_hap1-2

ATGGCAAGAATGTCGGAACCTCTAGCTGTTGGAAGAGTCATAGGAGATGTTCTTGATTCCTTCACCCCAACTACAAAAATGATTGTCACTTACAACACCAAGCTCGTCTGCAATGGACATGAGCTCTTCCCTTCTGCAGTCACTGCCAAACCTAAAGTTGAGATTCAAGGAGGCGACATGAGATCATTCTTCACTCTGgtattttctttctggcctccttttccgccgtttgtttttttatcagatctaccagatcgctaacttcagttattttcacagGTAATGACAGACCCAGATGTTCCTGGCCCTAGTGATCCTT**ATTTGAAAGAGCACC‑‑‑ACTGg**tatatcttacagttaatactgtctatatagtatcaaacctagaaacgaagaaacaaagacaataactattcagataattattaataacttcaatgttcaaactacatatatatgttgtcatattctagaaatttgcagtattgacctaacaaattgtccaggttaatttatagatcaattgcgctcctgcttgtaatttccagattcaaagaaaagattagttatattaattattgttattttattctttctcctttacatggtcattcacgtattcagaattgcaatcagaaaatctttggatctggaaataaaagcaagtaaaaattacatattatgtcacgaactcttaactagaaaaactagcaagttataattgtggagtatgtacatgccaataaacttggatctaacagtttttccttgaaattgaactatacagGATTGTGACAGACATTCCTGGCACCACAGATGCAACATTTGgtaggttaattcacactagttcatgagctaccagtacgttatacagctagtgatgatcagcactgatattttgtatatactacatcaattaacagGAAGAGAAGTGGTGAGCTACGAGATGCCAAGGCCAAACATAGGCATCCACAGGTTTGTGTTTGTTCTCTTCATGCAAAAACGAAGGCAGTCGGTGAACCCGCCTTCCTCAAGGGATCACTTCAACACCCGAACCTTCGCAGCCGAAAACGACCTTGGTATTCCTGTTGCTGCCGTTTACTTCAATGCACAGAGAGAAACGGCAGCAAGAAGACGCTAG >LineC24_TFL1-B_protein

MARMSEPLAVGRVIGDVLDSFTPTTKMIVTYNTKLVCNGHELFPSAVTAKPKVEIQGGDMRSFF

TLVMTDPDVPGPSDPYLKEH‑HWIVTDIPGTTDATFGREVVSYEMPRPNIGIHRFVFVLFMQKRRQSVNPPSSRDHFNTRTFAAENDLGIPVAAVYFNAQRETAARRR*

>LineC21_TFL1-B_DNA_hap1-2

ATGGCAAGAATGTCGGAACCTCTAGCTGTTGGAAGAGTCATAGGAGATGTTCTTGATTCCTTCACCCCAACTACAAAAATGATTGTCACTTACAACACCAAGCTCGTCTGCAATGGACATGAGCTCTTCCCTTCTGCAGTCACTGCCAAACCTAAAGTTGAGATTCAAGGAGGCGACATGAGATCATTCTTCACTCTGgtattttctttctggcctccttttccgccgtttgtttttttatcagatctaccagatcgctaacttcagttattttcacagGTAATGACAGACCCAGATGTTCCTGGCCCTAGTGATCCTT**ATTTGAAAGAGCACCTG‑‑‑‑Gg**tatatcttacagttaatactgtctatatagtatcaaacctagaaacgaagaaacaaagacaataactattcagataattattaataacttcaatgttcaaactacatatatatgttgtcatattctagaaatttgcagtattgacctaacaaattgtccaggttaatttatagatcaattgcgctcctgcttgtaatttccagattcaaagaaaagattagttatattaattattgttattttattctttctcctttacatggtcattcacgtattcagaattgcaatcagaaaatctttggatctggaaataaaagcaagtaaaaattacatattatgtcacgaactcttaactagaaaaactagcaagttataattgtggagtatgtacatgccaataaacttggatctaacagtttttccttgaaattgaactatacagGATTGTGACAGACATTCCTGGCACCACAGATGCAACATTTGgtaggttaattcacactagttcatgagctaccagtacgttatacagctagtgatgatcagcactgatattttgtatatactacatcaattaacagGAAGAGAAGTGGTGAGCTACGAGATGCCAAGGCCAAACATAGGCATCCACAGGTTTGTGTTTGTTCTCTTCATGCAAAAACGAAGGCAGTCGGTGAACCCGCCTTCCTCAAGGGATCACTTCAACACCCGAACCTTCGCAGCCGAAAACGACCTTGGTATTCCTGTTGCTGCCGTTTACTTCAATGCACAGAGAGAAACGGCAGCAAGAAGACGCTAG >LineC21_TFL1-B_protein

MARMSEPLAVGRVIGDVLDSFTPTTKMIVTYNTKLVCNGHELFPSAVTAKPKVEIQGGDMRSFFTLVMTDPDVPGPSDPYLKEHL**DCDRHSWHHRCNIW***

>LineC19_TFL1-B_DNA_hap1-2

ATGGCAAGAATGTCGGAACCTCTAGCTGTTGGAAGAGTCATAGGAGATGTTCTTGATTCCTTCACCCCAACTACAAAAATGATTGTCACTTACAACACCAAGCTCGTCTGCAATGGACATGAGCTCTTCCCTTCTGCAGTCACTGCCAAACCTAAAGTTGAGATTCAAGGAGGCGACATGAGATCATTCTTCACTCTGgtattttctttctggcctccttttccgccgtttgtttttttatcagatctaccagatcgctaacttcagttattttcacagGTAATGACAGACCCAGATGTTCCTGGCCCTAGTGATCCTT**ATTTGAAAGAGCACCT‑CACTGg**tatatcttacagttaatactgtctatatagtatcaaacctagaaacgaagaaacaaagacaataactattcagataattattaataacttcaatgttcaaactacatatatatgttgtcatattctagaaatttgcagtattgacctaacaaattgtccaggttaatttatagatcaattgcgctcctgcttgtaatttccagattcaaagaaaagattagttatattaattattgttattttattctttctcctttacatggtcattcacgtattcagaattgcaatcagaaaatctttggatctggaaataaaagcaagtaaaaattacatattatgtcacgaactcttaactagaaaaactagcaagttataattgtggagtatgtacatgccaataaacttggatctaacagtttttccttgaaattgaactatacagGATTGTGACAGACATTCCTGGCACCACAGATGCAACATTTGgtaggttaattcacactagttcatgagctaccagtacgttatacagctagtgatgatcagcactgatattttgtatatactacatcaattaacagGAAGAGAAGTGGTGAGCTACGAGATGCCAAGGCCAAACATAGGCATCCACAGGTTTGTGTTTGTTCTCTTCATGCAAAAACGAAGGCAGTCGGTGAACCCGCCTTCCTCAAGGGATCACTTCAACACCCGAACCTTCGCAGCCGAAAACGACCTTGGTATTCCTGTTGCTGCCGTTTACTTCAATGCACAGAGAGAAACGGCAGCAAGAAGACGCTAG >LineC19_TFL1-B_protein

MARMSEPLAVGRVIGDVLDSFTPTTKMIVTYNTKLVCNGHELFPSAVTAKPKVEIQGGDMRSFFTLVMTDPDVPGPSDPYLKEHL**TGL***

>Line14_TFL1-B_DNA_hap1-2

ATGGCAAGAATGTCGGAACCTCTAGCTGTTGGAAGAGTCATAGGAGATGTTCTTGATTCCTTCACCCCAACTACAAAAATGATTGTCACTTACAACACCAAGCTCGTCTGCAATGGACATGAGCTCTTCCCTTCTGCAGTCACTGCCAAACCTAAAGTTGAGATTCAAGGAGGCGACATGAGATCATTCTTCACTCTGgtattttctttctggcctccttttccgccgtttgtttttttatcagatctaccagatcgctaacttcagttattttcacagGTAATGACAGACCCAGATGTTCCTGGCCCTAGTGATCCTT**ATTTGAAAGAGCACCTG‑ACTGg**tatatcttacagttaatactgtctatatagtatcaaacctagaaacgaagaaacaaagacaataactattcagataattattaataacttcaatgttcaaactacatatatatgttgtcatattctagaaatttgcagtattgacctaacaaattgtccaggttaatttatagatcaattgcgctcctgcttgtaatttccagattcaaagaaaagattagttatattaattattgttattttattctttctcctttacatggtcattcacgtattcagaattgcaatcagaaaatctttggatctggaaataaaagcaagtaaaaattacatattatgtcacgaactcttaactagaaaaactagcaagttataattgtggagtatgtacatgccaataaacttggatctaacagtttttccttgaaattgaactatacagGATTGTGACAGACATTCCTGGCACCACAGATGCAACATTTGgtaggttaattcacactagttcatgagctaccagtacgttatacagctagtgatgatcagcactgatattttgtatatactacatcaattaacagGAAGAGAAGTGGTGAGCTACGAGATGCCAAGGCCAAACATAGGCATCCACAGGTTTGTGTTTGTTCTCTTCATGCAAAAACGAAGGCAGTCGGTGAACCCGCCTTCCTCAAGGGATCACTTCAACACCCGAACCTTCGCAGCCGAAAACGACCTTGGTATTCCTGTTGCTGCCGTTTACTTCAATGCACAGAGAGAAACGGCAGCAAGAAGACGCTAG

>Line14_TFL1-B_protein

MARMSEPLAVGRVIGDVLDSFTPTTKMIVTYNTKLVCNGHELFPSAVTAKPKVEIQGGDMRSFFTLVMTDPDVPGPSDPYLKEHL**TGL***

>Line15_TFL1-B_DNA

ATGGCAAGAATGTCGGAACCTCTAGCTGTTGGAAGAGTCATAGGAGATGTTCTTGATTCCTTCACCCCAACTACAAAAATGATTGTCACTTACAACACCAAGCTCGTCTGCAATGGACATGAGCTCTTCCCTTCTGCAGTCACTGCCAAACCTAAAGTTGAGATTCAAGGAGGCGACATGAGATCATTCTTCACTCTGgtattttctttctggcctccttttccgccgtttgtttttttatcagatctaccagatcgctaacttcagttattttcac**‑‑‑‑‑‑‑‑‑‑‑‑‑‑‑‑‑‑‑‑‑‑‑‑‑‑‑‑‑‑‑‑‑‑‑‑‑‑‑‑‑‑‑‑‑‑‑‑‑‑‑‑‑‑‑‑‑‑‑‑‑‑Tgg**tatatcttacagttaatactgtctatatagtatcaaacctagaaacgaagaaacaaagacaataactattcagataattattaataacttcaatgttcaaactacatatatatgttgtcatattctagaaatttgcagtattgacctaacaaattgtccaggttaatttatagatcaattgcgctcctgcttgtaatttccagattcaaagaaaagattagttatattaattattgttattttattctttctcctttacatggtcattcacgtattcagaattgcaatcagaaaatctttggatctggaaataaaagcaagtaaaaattacatattatgtcacgaactcttaactagaaaaactagcaagttataattgtggagtatgtacatgccaataaacttggatctaacagtttttccttgaaattgaactatacagGATTGTGACAGACATTCCTGGCACCACAGATGCAACATTTGgtaggttaattcacactagttcatgagctaccagtacgttatacagctagtgatgatcagcactgatattttgtatatactacatcaattaacagGAAGAGAAGTGGTGAGCTACGAGATGCCAAGGCCAAACATAGGCATCCACAGGTTTGTGTTTGTTCTCTTCATGCAAAAACGAAGGCAGTCGGTGAACCCGCCTTCCTCAAGGGATCACTTCAACACCCGAACCTTCGCAGCCGAAAACGACCTTGGTATTCCTGTTGCTGCCGTTTACTTCAATGCACAGAGAGAAACGGCAGCAAGAAGACGCTAG

>Line15_TFL1-B_protein

MARMSEPLAVGRVIGDVLDSFTPTTKMIVTYNTKLVCNGHELFPSAVTAKPKVEIQGGDMRSFFTL**DCDRHSWHHRCNIW***

>Line38_TFL1-B_DNA_hap1

ATGGCAAGAATGTCGGAACCTCTAGCTGTTGGAAGAGTCATAGGAGATGTTCTTGATTCCTTCACCCCAACTACAAAAATGATTGTCACTTACAACACCAAGCTCGTCTGCAATGGACATGAGCTCTTCCCTTCTGCAGTCACTGCCAAACCTAAAGTTGAGATTCAAGGAGGCGA**CATGAGATCATTCTTCAACTCTGg**tattttctttctggcctccttttccgccgtttgtttttttatcagatctaccagatcgctaacttcagttattttcacagGTAATGACAGACCCAGATGTTCCTGGCCCTAGTGATCCTTATTTGAAAGAGCACCTG**‑‑‑‑‑**gtatatcttacagttaatactgtctatatagtatcaaacctagaaacgaagaaacaaagacaataactattcagataattattaataacttcaatgttcaaactacatatatatgttgtcatattctagaaatttgcagtattgacctaacaaattgtccaggttaatttatagatcaattgcgctcctgcttgtaatttccagattcaaagaaaagattagttatattaattattgttattttattctttctcctttacatggtcattcacgtattcagaattgcaatcagaaaatctttggatctggaaataaaagcaagtaaaaattacatattatgtcacgaactcttaactagaaaaactagcaagttataattgtggagtatgtacatgccaataaacttggatctaacagtttttccttgaaattgaactatacagGATTGTGACAGACATTCCTGGCACCACAGATGCAACATTTGgtaggttaattcacactagttcatgagctaccagtacgttatacagctagtgatgatcagcactgatattttgtatatactacatcaattaacagGAAGAGAAGTGGTGAGCTACGAGATGCCAAGGCCAAACATAGGCATCCACAGGTTTGTGTTTGTTCTCTTCATGCAAAAACGAAGGCAGTCGGTGAACCCGCCTTCCTCAAGGGATCACTTCAACACCCGAACCTTCGCAGCCGAAAACGACCTTGGTATTCCTGTTGCTGCCGTTTACTTCAATGCACAGAGAGAAACGGCAGCAAGAAGACGCTAG >Line38_TFL1-B_protein

MARMSEPLAVGRVIGDVLDSFTPTTKMIVTYNTKLVCNGHELFPSAVTAKPKVEIQGGDMRSFF**NSGNDRPRCSWP***

>Line38_TFL1-B_DNA_hap2

ATGGCAAGAATGTCGGAACCTCTAGCTGTTGGAAGAGTCATAGGAGATGTTCTTGATTCCTTCACCCCAACTACAAAAATGATTGTCACTTACAACACCAAGCTCGTCTGCAATGGACATGAGCTCTTCCCTTCTGCAGTCACTGCCAAACCTAAAGTTGAGATTCAAGGAGGCGACATGAGATCATTCTTCACTCTGgtattttctttctggcctccttttccgccgtttgtttttttatcagatctaccagatcgctaacttcagttattttcacagGTAATGACAGACCCAGATGTTCCTGGCCCTAGTGATCCTTATTTGAAAGAGCACCTG**G**CACTGgtatatcttacagttaatactgtctatatagtatcaaacctagaaacgaagaaacaaagacaataactattcagataattattaataacttcaatgttcaaactacatatatatgttgtcatattctagaaatttgcagtattgacctaacaaattgtccaggttaatttatagatcaattgcgctcctgcttgtaatttccagattcaaagaaaagattagttatattaattattgttattttattctttctcctttacatggtcattcacgtattcagaattgcaatcagaaaatctttggatctggaaataaaagcaagtaaaaattacatattatgtcacgaactcttaactagaaaaactagcaagttataattgtggagtatgtacatgccaataaacttggatctaacagtttttccttgaaattgaactatacagGATTGTGACAGACATTCCTGGCACCACAGATGCAACATTTGgtaggttaattcacactagttcatgagctaccagtacgttatacagctagtgatgatcagcactgatattttgtatatactacatcaattaacagGAAGAGAAGTGGTGAGCTACGAGATGCCAAGGCCAAACATAGGCATCCACAGGTTTGTGTTTGTTCTCTTCATGCAAAAACGAAGGCAGTCGGTGAACCCGCCTTCCTCAAGGGATCACTTCAACACCCGAACCTTCGCAGCCGAAAACGACCTTGGTATTCCTGTTGCTGCCGTTTACTTCAATGCACAGAGAGAAACGGCAGCAAGAAGACGCTAG >Line38_TFL1-B_protein_hap2

MARMSEPLAVGRVIGDVLDSFTPTTKMIVTYNTKLVCNGHELFPSAVTAKPKVEIQGGDMRSFF

TLVMTDPDVPGPSDPYLKEHL**ALFTWSFTYSELQSENLWIWK***

>LineXXXX_TFL1-C_DNA

ATGGCAAGAATGTCGGAACCTCTAGCTGTTGGAAGAGTCATAGGAGATGTTCTTGATTCCTTCACCCCAACTACAAAAATGATTGTCACTTACAACACCAAGCTCGTCTGCAATGGACATGAGCTCTTCCCTTCTGCAGTCACTGCCAAACCGAGAGTTGAGATTCAAGGAGGCGACATGAGATCATTCTTCACTCTGgtatttctttctggcctccttttcggccgtttgttttttttatcagatctaccagatcgctaactttatttattttcacagGTAATGACAGACCCAGATGTTCCTGGCCCTAGTGATCCTTATTTGAAAGAGCACCTGCACTGgtatatcttacagttaatactgtctatatattatcaaacctagaaacgaagaaacaaagacaataactattcagataattattaataacttcaatgttcaaactagatatatatgttgtcatattctagaaatttgcagtattgaccgaacaaattgtccaggttaatttatagatcaattgcgctcctgcttgtaatttccagattcaaagaaaagattagttattttaattattgttaatttattctttctcctttacatggtcattcatgtattcagaattgtaatccgaaaatctttggatctggaaataaaagcaagtaaaaattacatattatgtcacgaactcttaactagaaaaactagcaagttataattgtggagtatagacatgccaataagcttggatctaacagttttgccttgaaattgaactatacagGATTGTGACAGACATTCCTGGCACCACAGATGCTACATTTGgtaggttaattcacactagttcatgagctaccagtacgttatacagctagtgatgatcagcactgatattgtgtatatactacattaattaacagGAAGAGAAGTGGTGAGCTACGAGATGCCAAGGCCAAACATAGGCATCCACAGGTTTGTGTTTGTTCTCTTCATGCAAAAACGAAGGCAGTCGGTGAACCCGCCTTCCTCAAGGGATCACTTCAACACCCGAACCTTCGCAGCCGAAAACGACCTTGGTGTTCCTGTTGCAGCCGTTTACTTCAATGCACAGAGAGAAACGGCAGCAAGAAGACGCTAG

>LineXXXX_TFL1-C_protein

MARMSEPLAVGRVIGDVLDSFTPTTKMIVTYNTKLVCNGHELFPSAVTAKPRVEIQGGDMRSFFTLVMTDPDVPGPSDPYLKEHLHWIVTDIPGTTDATFGREVVSYEMPRPNIGIHRFVFVLFMQKRRQSVNPPSSRDHFNTRTFAAENDLGVPVAAVYFNAQRETAARRR*

>LineC20_TFL1-C_DNA_hap1

ATGGCAAGAATGTCGGAACCTCTAGCTGTTGGAAGAGTCATAGGAGATGTTCTTGATTCCTTCACCCCAACTACAAAAATGATTGTCACTTACAACACCAAGCTCGTCTGCAATGGACATGAGCTCTTCCCTTCTGCAGTCACTGCCAAACCGAGAGTTGAGATTCAAGGAGGCGA**CATGAGATCATTCTTCA‑‑‑‑‑‑‑‑‑‑‑‑‑**ttctggcctccttttcggccgtttgttttttttatcagatctaccagatcgctaactttatttattttcacagGTAATGACAGACCCAGATGTTCCTGGCCCTAGTGATCCTTATTTGAAAGAGCACC**‑‑‑**ACTGgtatatcttacagttaatactgtctatatattatcaaacctagaaacgaagaaacaaagacaataactattcagataattattaataacttcaatgttcaaactagatatatatgttgtcatattctagaaatttgcagtattgaccgaacaaattgtccaggttaatttatagatcaattgcgctcctgcttgtaatttccagattcaaagaaaagattagttattttaattattgttaatttattctttctcctttacatggtcattcatgtattcagaattgtaatccgaaaatctttggatctggaaataaaagcaagtaaaaattacatattatgtcacgaactcttaactagaaaaactagcaagttataattgtggagtatagacatgccaataagcttggatctaacagttttgccttgaaattgaactatacagGATTGTGACAGACATTCCTGGCACCACAGATGCTACATTTGgtaggttaattcacactagttcatgagctaccagtacgttatacagctagtgatgatcagcactgatattgtgtatatactacattaattaacagGAAGAGAAGTGGTGAGCTACGAGATGCCAAGGCCAAACATAGGCATCCACAGGTTTGTGTTTGTTCTCTTCATGCAAAAACGAAGGCAGTCGGTGAACCCGCCTTCCTCAAGGGATCACTTCAACACCCGAACCTTCGCAGCCGAAAACGACCTTGGTGTTCCTGTTGCAGCCGTTTACTTCAATGCACAGAGAGAAACGGCAGCAAGAAGACGCTAG

>LineC20_TFL1-C_protein_hap1

MARMSEPLAVGRVIGDVLDSFTPTTKMIVTYNTKLVCNGHELFPSAVTAKPRVEIQGGDMRSFF**ILASFSAVCFFYQIYQIANFIYFHR***

>LineC20_TFL1-C_DNA_hap2

ATGGCAAGAATGTCGGAACCTCTAGCTGTTGGAAGAGTCATAGGAGATGTTCTTGATTCCTTCACCCCAACTACAAAAATGATTGTCACTTACAACACCAAGCTCGTCTGCAATGGACATGAGCTCTTCCCTTCTGCAGTCACTGCCAAACCGAGAGTTGAGATTCAAGGAGGCGACATGAGATCATTCTTCACTCTGgtatttctttctggcctccttttcggccgtttgttttttttatcagatctaccagatcgctaactttatttattttcacagGTAATGACAGACCCAGATGTTCCTGGCCCTAGTGATCCTTATTTGAAAGAGCACCTG‑‑‑‑‑gtatatcttacagttaatactgtctatatattatcaaacctagaaacgaagaaacaaagacaataactattcagataattattaataacttcaatgttcaaactagatatatatgttgtcatattctagaaatttgcagtattgaccgaacaaattgtccaggttaatttatagatcaattgcgctcctgcttgtaatttccagattcaaagaaaagattagttattttaattattgttaatttattctttctcctttacatggtcattcatgtattcagaattgtaatccgaaaatctttggatctggaaataaaagcaagtaaaaattacatattatgtcacgaactcttaactagaaaaactagcaagttataattgtggagtatagacatgccaataagcttggatctaacagttttgccttgaaattgaactatacagGATTGTGACAGACATTCCTGGCACCACAGATGCTACATTTGgtaggttaattcacactagttcatgagctaccagtacgttatacagctagtgatgatcagcactgatattgtgtatatactacattaattaacagGAAGAGAAGTGGTGAGCTACGAGATGCCAAGGCCAAACATAGGCATCCACAGGTTTGTGTTTGTTCTCTTCATGCAAAAACGAAGGCAGTCGGTGAACCCGCCTTCCTCAAGGGATCACTTCAACACCCGAACCTTCGCAGCCGAAAACGACCTTGGTGTTCCTGTTGCAGCCGTTTACTTCAATGCACAGAGAGAAACGGCAGCAAGAAGACGCTAG

>LineXXXX_TFL1-C_protein_hap2

MARMSEPLAVGRVIGDVLDSFTPTTKMIVTYNTKLVCNGHELFPSAVTAKPRVEIQGGDMRSFF

TLVMTDPDVPGPSDPYLKEHL**VYLTVNTVYILSNLETKKQRQ***

>LineC24_TFL1-C_DNA_hap1

ATGGCAAGAATGTCGGAACCTCTAGCTGTTGGAAGAGTCATAGGAGATGTTCTTGATTCCTTCACCCCAACTACAAAAATGATTGTCACTTACAACACCAAGCTCGTCTGCAATGGACATGAGCTCTTCCCTTCTGCAGTCACTGCCAAACCGAGAGTTGAGATTCAAGGAGGCGACATGAGATCATTCTTCACTCTGgtatttctttctggcctccttttcggccgtttgttttttttatcagatctaccagatcgctaactttatttattttcacagGTAATGACAGACCCAGATGTTCCTGGCCCTAGTGATCCTT**ATTTGAAAGAGCACCTG‑‑‑‑‑g**tatatcttacagttaatactgtctatatattatcaaacctagaaacgaagaaacaaagacaataactattcagataattattaataacttcaatgttcaaactagatatatatgttgtcatattctagaaatttgcagtattgaccgaacaaattgtccaggttaatttatagatcaattgcgctcctgcttgtaatttccagattcaaagaaaagattagttattttaattattgttaatttattctttctcctttacatggtcattcatgtattcagaattgtaatccgaaaatctttggatctggaaataaaagcaagtaaaaattacatattatgtcacgaactcttaactagaaaaactagcaagttataattgtggagtatagacatgccaataagcttggatctaacagttttgccttgaaattgaactatacagGATTGTGACAGACATTCCTGGCACCACAGATGCTACATTTGgtaggttaattcacactagttcatgagctaccagtacgttatacagctagtgatgatcagcactgatattgtgtatatactacattaattaacagGAAGAGAAGTGGTGAGCTACGAGATGCCAAGGCCAAACATAGGCATCCACAGGTTTGTGTTTGTTCTCTTCATGCAAAAACGAAGGCAGTCGGTGAACCCGCCTTCCTCAAGGGATCACTTCAACACCCGAACCTTCGCAGCCGAAAACGACCTTGGTGTTCCTGTTGCAGCCGTTTACTTCAATGCACAGAGAGAAACGGCAGCAAGAAGACGCTAG

>LineC24_TFL1-C_protein_hap1

MARMSEPLAVGRVIGDVLDSFTPTTKMIVTYNTKLVCNGHELFPSAVTAKPRVEIQGGDMRSFFTLVMTDPDVPGPSDPYLKEHL**DCDRHSWHHRCYIW***

>LineC24_TFL1-C_DNA_hap2

ATGGCAAGAATGTCGGAACCTCTAGCTGTTGGAAGAGTCATAGGAGATGTTCTTGATTCCTTCACCCCAACTACAAAAATGATTGTCACTTACAACACCAAGCTCGTCTGCAATGGACATGAGCTCTTCCCTTCTGCAGTCACTGCCAAACCGAGAGTTGAGATTCAAGGAGGCGACATGAGATCATTCTTCACTCTGgtatttctttctggcctccttttcggccgtttgttttttttatcagatctaccagatcgctaactttatttattttcacagGTAATGACAGACCCAGATGTTCCTGGCCCTAGTGATCCTT**ATTTGAAAGAGCAC‑‑‑‑‑‑‑‑g**tatatcttacagttaatactgtctatatattatcaaacctagaaacgaagaaacaaagacaataactattcagataattattaataacttcaatgttcaaactagatatatatgttgtcatattctagaaatttgcagtattgaccgaacaaattgtccaggttaatttatagatcaattgcgctcctgcttgtaatttccagattcaaagaaaagattagttattttaattattgttaatttattctttctcctttacatggtcattcatgtattcagaattgtaatccgaaaatctttggatctggaaataaaagcaagtaaaaattacatattatgtcacgaactcttaactagaaaaactagcaagttataattgtggagtatagacatgccaataagcttggatctaacagttttgccttgaaattgaactatacagGATTGTGACAGACATTCCTGGCACCACAGATGCTACATTTGgtaggttaattcacactagttcatgagctaccagtacgttatacagctagtgatgatcagcactgatattgtgtatatactacattaattaacagGAAGAGAAGTGGTGAGCTACGAGATGCCAAGGCCAAACATAGGCATCCACAGGTTTGTGTTTGTTCTCTTCATGCAAAAACGAAGGCAGTCGGTGAACCCGCCTTCCTCAAGGGATCACTTCAACACCCGAACCTTCGCAGCCGAAAACGACCTTGGTGTTCCTGTTGCAGCCGTTTACTTCAATGCACAGAGAGAAACGGCAGCAAGAAGACGCTAG

>LineC24_TFL1-C_protein_hap2

MARMSEPLAVGRVIGDVLDSFTPTTKMIVTYNTKLVCNGHELFPSAVTAKPRVEIQGGDMRSFFTLVMTDPDVPGPSDPYLKEH**VYLTVNTVYILSNLETKKQRQ***

>LineC22_TFL1-C_DNA

ATGGCAAGAATGTCGGAACCTCTAGCTGTTGGAAGAGTCATAGGAGATGTTCTTGATTCCTTCACCCCAACTACAAAAATGATTGTCACTTACAACACCAAGCTCGTCTGCAATGGACATGAGCTCTTCCCTTCTGCAGTCACTGCCAAACCGAGAGTTGAGATTCAAGGAGGCGACATGAGATCATTCTTCACTCTGgtatttctttctggcctccttttcggccgtttgttttttttatcagatctaccagatcgctaactttatttattttcacagGTAATGACAGACCCAGATGTTCCTGGCCCTAGTGATCCTT**ATTTGAAAGAGCACCTG‑‑‑‑‑g**tatatcttacagttaatactgtctatatattatcaaacctagaaacgaagaaacaaagacaataactattcagataattattaataacttcaatgttcaaactagatatatatgttgtcatattctagaaatttgcagtattgaccgaacaaattgtccaggttaatttatagatcaattgcgctcctgcttgtaatttccagattcaaagaaaagattagttattttaattattgttaatttattctttctcctttacatggtcattcatgtattcagaattgtaatccgaaaatctttggatctggaaataaaagcaagtaaaaattacatattatgtcacgaactcttaactagaaaaactagcaagttataattgtggagtatagacatgccaataagcttggatctaacagttttgccttgaaattgaactatacagGATTGTGACAGACATTCCTGGCACCACAGATGCTACATTTGgtaggttaattcacactagttcatgagctaccagtacgttatacagctagtgatgatcagcactgatattgtgtatatactacattaattaacagGAAGAGAAGTGGTGAGCTACGAGATGCCAAGGCCAAACATAGGCATCCACAGGTTTGTGTTTGTTCTCTTCATGCAAAAACGAAGGCAGTCGGTGAACCCGCCTTCCTCAAGGGATCACTTCAACACCCGAACCTTCGCAGCCGAAAACGACCTTGGTGTTCCTGTTGCAGCCGTTTACTTCAATGCACAGAGAGAAACGGCAGCAAGAAGACGCTAG

>LineC22_TFL1-C_protein

MARMSEPLAVGRVIGDVLDSFTPTTKMIVTYNTKLVCNGHELFPSAVTAKPRVEIQGGDMRSFFTLVMTDPDVPGPSDPYLKEHL**DCDRHSWHHRCYIW***

>LineC21_TFL1-C_DNA

ATGGCAAGAATGTCGGAACCTCTAGCTGTTGGAAGAGTCATAGGAGATGTTCTTGATTCCTTCACCCCAACTACAAAAATGATTGTCACTTACAACACCAAGCTCGTCTGCAATGGACATGAGCTCTTCCCTTCTGCAGTCACTGCCAAACCGAGAGTTGAGATTCAAGGAGGCGACATGAGATCATTCTTCACTCTGgtatttctttctggcctccttttcggccgtttgttttttttatcagatctaccagatcgctaactttatttattttcacagGTAATGACAGACCCAGATGTTCCTGGCCCTAGTGATCCTTATTTGAAAGAGCACCTGACAGTTATACTGgtatatcttacagttaatactgtctatatattatcaaacctagaaacgaagaaacaaagacaataactattcagataattattaataacttcaatgttcaaactagatatatatgttgtcatattctagaaatttgcagtattgaccgaacaaattgtccaggttaatttatagatcaattgcgctcctgcttgtaatttccagattcaaagaaaagattagttattttaattattgttaatttattctttctcctttacatggtcattcatgtattcagaattgtaatccgaaaatctttggatctggaaataaaagcaagtaaaaattacatattatgtcacgaactcttaactagaaaaactagcaagttataattgtggagtatagacatgccaataagcttggatctaacagttttgccttgaaattgaactatacagGATTGTGACAGACATTCCTGGCACCACAGATGCTACATTTGgtaggttaattcacactagttcatgagctaccagtacgttatacagctagtgatgatcagcactgatattgtgtatatactacattaattaacagGAAGAGAAGTGGTGAGCTACGAGATGCCAAGGCCAAACATAGGCATCCACAGGTTTGTGTTTGTTCTCTTCATGCAAAAACGAAGGCAGTCGGTGAACCCGCCTTCCTCAAGGGATCACTTCAACACCCGAACCTTCGCAGCCGAAAACGACCTTGGTGTTCCTGTTGCAGCCGTTTACTTCAATGCACAGAGAGAAACGGCAGCAAGAAGACGCTAG

>LineC21_TFL1-C_protein

MARMSEPLAVGRVIGDVLDSFTPTTKMIVTYNTKLVCNGHELFPSAVTAKPRVEIQGGDMRSFF

TLVMTDPDVPGPSDPYLKEHL**TVILDCDRHSWHHRCYIW***

>LineC19_TFL1-C_DNA_hap1

ATGGCAAGAATGTCGGAACCTCTAGCTGTTGGAAGAGTCATAGGAGATGTTCTTGATTCCTTCACCCCAACTACAAAAATGATTGTCACTTACAACACCAAGCTCGTCTGCAATGGACATGAGCTCTTCCCTTCTGCAGTCACTGCCAAACCGAGAGTTGAGATTCAAGGAGGCGACATGAGATCATTCTTCACTCTGgtatttctttctggcctccttttcggccgtttgttttttttatcagatctaccagatcgctaactttatttattttcacagGTAATGACAGACCCAGATGTTCCTGGCCCTAGTGATCCTTATTTGAAAGAGCACCTGCAC‑‑gtatatcttacagttaatactgtctatatattatcaaacctagaaacgaagaaacaaagacaataactattcagataattattaataacttcaatgttcaaactagatatatatgttgtcatattctagaaatttgcagtattgaccgaacaaattgtccaggttaatttatagatcaattgcgctcctgcttgtaatttccagattcaaagaaaagattagttattttaattattgttaatttattctttctcctttacatggtcattcatgtattcagaattgtaatccgaaaatctttggatctggaaataaaagcaagtaaaaattacatattatgtcacgaactcttaactagaaaaactagcaagttataattgtggagtatagacatgccaataagcttggatctaacagttttgccttgaaattgaactatacagGATTGTGACAGACATTCCTGGCACCACAGATGCTACATTTGgtaggttaattcacactagttcatgagctaccagtacgttatacagctagtgatgatcagcactgatattgtgtatatactacattaattaacagGAAGAGAAGTGGTGAGCTACGAGATGCCAAGGCCAAACATAGGCATCCACAGGTTTGTGTTTGTTCTCTTCATGCAAAAACGAAGGCAGTCGGTGAACCCGCCTTCCTCAAGGGATCACTTCAACACCCGAACCTTCGCAGCCGAAAACGACCTTGGTGTTCCTGTTGCAGCCGTTTACTTCAATGCACAGAGAGAAACGGCAGCAAGAAGACGCTAG

>LineC19_TFL1-C_protein_hap1

MARMSEPLAVGRVIGDVLDSFTPTTKMIVTYNTKLVCNGHELFPSAVTAKPRVEIQGGDMRSFFTLVMTDPDVPGPSDPYLKEHLH**DCDRHSWHHRCYIW***

>LineC19_TFL1-C_DNA_hap2

ATGGCAAGAATGTCGGAACCTCTAGCTGTTGGAAGAGTCATAGGAGATGTTCTTGATTCCTTCACCCCAACTACAAAAATGATTGTCACTTACAACACCAAGCTCGTCTGCAATGGACATGAGCTCTTCCCTTCTGCAGTCACTGCCAAACCGAGAGTTGAGATTCAAGGAGGCGACATGAGATCATTCTTCACTCTGgtatttctttctggcctccttttcggccgtttgttttttttatcagatctaccagatcgctaactttatttattttcacagGTAATGACAGACCCAGATGTTCCTGGCCCTAGTGATCCTTATTTGAAAGAGCACCTG‑‑‑‑‑gtatatcttacagttaatactgtctatatattatcaaacctagaaacgaagaaacaaagacaataactattcagataattattaataacttcaatgttcaaactagatatatatgttgtcatattctagaaatttgcagtattgaccgaacaaattgtccaggttaatttatagatcaattgcgctcctgcttgtaatttccagattcaaagaaaagattagttattttaattattgttaatttattctttctcctttacatggtcattcatgtattcagaattgtaatccgaaaatctttggatctggaaataaaagcaagtaaaaattacatattatgtcacgaactcttaactagaaaaactagcaagttataattgtggagtatagacatgccaataagcttggatctaacagttttgccttgaaattgaactatacagGATTGTGACAGACATTCCTGGCACCACAGATGCTACATTTGgtaggttaattcacactagttcatgagctaccagtacgttatacagctagtgatgatcagcactgatattgtgtatatactacattaattaacagGAAGAGAAGTGGTGAGCTACGAGATGCCAAGGCCAAACATAGGCATCCACAGGTTTGTGTTTGTTCTCTTCATGCAAAAACGAAGGCAGTCGGTGAACCCGCCTTCCTCAAGGGATCACTTCAACACCCGAACCTTCGCAGCCGAAAACGACCTTGGTGTTCCTGTTGCAGCCGTTTACTTCAATGCACAGAGAGAAACGGCAGCAAGAAGACGCTAG

>LineC19_TFL1-C_protein_hap2

MARMSEPLAVGRVIGDVLDSFTPTTKMIVTYNTKLVCNGHELFPSAVTAKPRVEIQGGDMRSFFTLVMTDPDVPGPSDPYLKEHL**DCDRHSWHHRCYIW***

>Line14_TFL1-C_DNA_hap1

ATGGCAAGAATGTCGGAACCTCTAGCTGTTGGAAGAGTCATAGGAGATGTTCTTGATTCCTTCACCCCAACTACAAAAATGATTGTCACTTACAACACCAAGCTCGTCTGCAATGGACATGAGCTCTTCCCTTCTGCAGTCACTGCCAAACCGAGAGTTGAGATTCAAGGAGGCGACATGAGATCATTCTTCACTCTGgtatttctttctggcctccttttcggccgtttgttttttttatcagatctaccagatcgctaactttatttattttcacagGTAATGACAGACCCAGATGTTCCTGGCCCTAGTGATCCTT**ATTTGAAAGAGCACCTGACACTGg**tatatcttacagttaatactgtctatatattatcaaacctagaaacgaagaaacaaagacaataactattcagataattattaataacttcaatgttcaaactagatatatatgttgtcatattctagaaatttgcagtattgaccgaacaaattgtccaggttaatttatagatcaattgcgctcctgcttgtaatttccagattcaaagaaaagattagttattttaattattgttaatttattctttctcctttacatggtcattcatgtattcagaattgtaatccgaaaatctttggatctggaaataaaagcaagtaaaaattacatattatgtcacgaactcttaactagaaaaactagcaagttataattgtggagtatagacatgccaataagcttggatctaacagttttgccttgaaattgaactatacagGATTGTGACAGACATTCCTGGCACCACAGATGCTACATTTGgtaggttaattcacactagttcatgagctaccagtacgttatacagctagtgatgatcagcactgatattgtgtatatactacattaattaacagGAAGAGAAGTGGTGAGCTACGAGATGCCAAGGCCAAACATAGGCATCCACAGGTTTGTGTTTGTTCTCTTCATGCAAAAACGAAGGCAGTCGGTGAACCCGCCTTCCTCAAGGGATCACTTCAACACCCGAACCTTCGCAGCCGAAAACGACCTTGGTGTTCCTGTTGCAGCCGTTTACTTCAATGCACAGAGAGAAACGGCAGCAAGAAGACGCTAG

>Line14_TFL1-C_protein_hap1

MARMSEPLAVGRVIGDVLDSFTPTTKMIVTYNTKLVCNGHELFPSAVTAKPRVEIQGGDMRSFFTLVMTDPDVPGPSDPYLKEHL**TLDCDRHSWHHRCYIW***

>Line14_TFL1-C_DNA_hap2

ATGGCAAGAATGTCGGAACCTCTAGCTGTTGGAAGAGTCATAGGAGATGTTCTTGATTCCTTCACCCCAACTACAAAAATGATTGTCACTTACAACACCAAGCTCGTCTGCAATGGACATGAGCTCTTCCCTTCTGCAGTCACTGCCAAACCGAGAGTTGAGATTCAAGGAGGCGACATGAGATCATTCTTCACTCTGgtatttctttctggcctccttttcggccgtttgttttttttatcagatctaccagatcgctaactttatttattttcacagGTAATGACAGACCCAGATGTTCCTGGCCCTAGTGATCCTT**ATTTGAAAGAGCACC‑‑‑ACTGg**tatatcttacagttaatactgtctatatattatcaaacctagaaacgaagaaacaaagacaataactattcagataattattaataacttcaatgttcaaactagatatatatgttgtcatattctagaaatttgcagtattgaccgaacaaattgtccaggttaatttatagatcaattgcgctcctgcttgtaatttccagattcaaagaaaagattagttattttaattattgttaatttattctttctcctttacatggtcattcatgtattcagaattgtaatccgaaaatctttggatctggaaataaaagcaagtaaaaattacatattatgtcacgaactcttaactagaaaaactagcaagttataattgtggagtatagacatgccaataagcttggatctaacagttttgccttgaaattgaactatacagGATTGTGACAGACATTCCTGGCACCACAGATGCTACATTTGgtaggttaattcacactagttcatgagctaccagtacgttatacagctagtgatgatcagcactgatattgtgtatatactacattaattaacagGAAGAGAAGTGGTGAGCTACGAGATGCCAAGGCCAAACATAGGCATCCACAGGTTTGTGTTTGTTCTCTTCATGCAAAAACGAAGGCAGTCGGTGAACCCGCCTTCCTCAAGGGATCACTTCAACACCCGAACCTTCGCAGCCGAAAACGACCTTGGTGTTCCTGTTGCAGCCGTTTACTTCAATGCACAGAGAGAAACGGCAGCAAGAAGACGCTAG

>Line14_TFL1-C_protein_hap2

MARMSEPLAVGRVIGDVLDSFTPTTKMIVTYNTKLVCNGHELFPSAVTAKPRVEIQGGDMRSFF

TLVMTDPDVPGPSDPYLKEH**‑**HWIVTDIPGTTDATFGREVVSYEMPRPNIGIHRFVFVLFMQKRRQSVNPPSSRDHFNTRTFAAENDLGVPVAAVYFNAQRETAARRR*

>Line15_TFL1-C_DNA_hap1

ATGGCAAGAATGTCGGAACCTCTAGCTGTTGGAAGAGTCATAGGAGATGTTCTTGATTCCTTCACCCCAACTACAAAAATGATTGTCACTTACAACACCAAGCTCGTCTGCAATGGACATGAGCTCTTCCCTTCTGCAGTCACTGCCAAACCGAGAGTTGAGATTCAAGGAGGCGACATGAGATCATTCTTCACTCTGgtatttctttctggcctccttttcggccgtttgttttttttatcagatctaccagatcgctaactttatttattttcacagGTAATGACAGACCCAGATGTTCCTGGCCCTAGTGATCCTT**ATTTGAAAGAGCACCTGACACTGg**tatatcttacagttaatactgtctatatattatcaaacctagaaacgaagaaacaaagacaataactattcagataattattaataacttcaatgttcaaactagatatatatgttgtcatattctagaaatttgcagtattgaccgaacaaattgtccaggttaatttatagatcaattgcgctcctgcttgtaatttccagattcaaagaaaagattagttattttaattattgttaatttattctttctcctttacatggtcattcatgtattcagaattgtaatccgaaaatctttggatctggaaataaaagcaagtaaaaattacatattatgtcacgaactcttaactagaaaaactagcaagttataattgtggagtatagacatgccaataagcttggatctaacagttttgccttgaaattgaactatacagGATTGTGACAGACATTCCTGGCACCACAGATGCTACATTTGgtaggttaattcacactagttcatgagctaccagtacgttatacagctagtgatgatcagcactgatattgtgtatatactacattaattaacagGAAGAGAAGTGGTGAGCTACGAGATGCCAAGGCCAAACATAGGCATCCACAGGTTTGTGTTTGTTCTCTTCATGCAAAAACGAAGGCAGTCGGTGAACCCGCCTTCCTCAAGGGATCACTTCAACACCCGAACCTTCGCAGCCGAAAACGACCTTGGTGTTCCTGTTGCAGCCGTTTACTTCAATGCACAGAGAGAAACGGCAGCAAGAAGACGCTAGC

>Line15_TFL1-C_protein_hap1

MARMSEPLAVGRVIGDVLDSFTPTTKMIVTYNTKLVCNGHELFPSAVTAKPRVEIQGGDMRSFFTLVMTDPDVPGPSDPYLKEHL**TLDCDRHSWHHRCYIW***

>Line15_TFL1-C_DNA_hap2

ATGGCAAGAATGTCGGAACCTCTAGCTGTTGGAAGAGTCATAGGAGATGTTCTTGATTCCTTCACCCCAACTACAAAAATGATTGTCACTTACAACACCAAGCTCGTCTGCAATGGACATGAGCTCTTCCCTTCTGCAGTCACTGCCAAACCGAGAGTTGAGATTCAAGGAGGCGACATGAGATCATTCTTCACTCTGgtatttctttctggcctccttttcggccgtttgttttttttatcagatctaccagatcgctaactttatttattttcacagGTAATGACAGACCCAGATGTTCCTGGCCCTAGTGATCCTTATTTGAAAGAGCACCTG**‑‑‑‑‑**gtatatcttacagttaatactgtctatatattatcaaacctagaaacgaagaaacaaagacaataactattcagataattattaataacttcaatgttcaaactagatatatatgttgtcatattctagaaatttgcagtattgaccgaacaaattgtccaggttaatttatagatcaattgcgctcctgcttgtaatttccagattcaaagaaaagattagttattttaattattgttaatttattctttctcctttacatggtcattcatgtattcagaattgtaatccgaaaatctttggatctggaaataaaagcaagtaaaaattacatattatgtcacgaactcttaactagaaaaactagcaagttataattgtggagtatagacatgccaataagcttggatctaacagttttgccttgaaattgaactatacagGATTGTGACAGACATTCCTGGCACCACAGATGCTACATTTGgtaggttaattcacactagttcatgagctaccagtacgttatacagctagtgatgatcagcactgatattgtgtatatactacattaattaacagGAAGAGAAGTGGTGAGCTACGAGATGCCAAGGCCAAACATAGGCATCCACAGGTTTGTGTTTGTTCTCTTCATGCAAAAACGAAGGCAGTCGGTGAACCCGCCTTCCTCAAGGGATCACTTCAACACCCGAACCTTCGCAGCCGAAAACGACCTTGGTGTTCCTGTTGCAGCCGTTTACTTCAATGCACAGAGAGAAACGGCAGCAAGAAGACGCTAG

>Line15_TFL1-C_protein_hap2

MARMSEPLAVGRVIGDVLDSFTPTTKMIVTYNTKLVCNGHELFPSAVTAKPRVEIQGGDMRSFFTLVMTDPDVPGPSDPYLKEHL**DCDRHSWHHRCYIW***

>Line38_TFL1-C_DNA_hap1

ATGGCAAGAATGTCGGAACCTCTAGCTGTTGGAAGAGTCATAGGAGATGTTCTTGATTCCTTCACCCCAACTACAAAAATGATTGTCACTTACAACACCAAGCTCGTCTGCAATGGACATGAGCTCTTCCCTTCTGCAGTCACTGCCAAACCGAGAGTTGAGATTCAAGGAGGCGACATGAGATCATTCTTCACTCTGgtatttctttctggcctccttttcggccgtttgttttttttatcagatctaccagatcgctaactttatttattttcacagGTAATGACAGACCCAGATGTTCCTGGCCCTAGTGATCCTT**ATTTGAAAGAGCACCTG‑‑‑‑‑g**tatatcttacagttaatactgtctatatattatcaaacctagaaacgaagaaacaaagacaataactattcagataattattaataacttcaatgttcaaactagatatatatgttgtcatattctagaaatttgcagtattgaccgaacaaattgtccaggttaatttatagatcaattgcgctcctgcttgtaatttccagattcaaagaaaagattagttattttaattattgttaatttattctttctcctttacatggtcattcatgtattcagaattgtaatccgaaaatctttggatctggaaataaaagcaagtaaaaattacatattatgtcacgaactcttaactagaaaaactagcaagttataattgtggagtatagacatgccaataagcttggatctaacagttttgccttgaaattgaactatacagGATTGTGACAGACATTCCTGGCACCACAGATGCTACATTTGgtaggttaattcacactagttcatgagctaccagtacgttatacagctagtgatgatcagcactgatattgtgtatatactacattaattaacagGAAGAGAAGTGGTGAGCTACGAGATGCCAAGGCCAAACATAGGCATCCACAGGTTTGTGTTTGTTCTCTTCATGCAAAAACGAAGGCAGTCGGTGAACCCGCCTTCCTCAAGGGATCACTTCAACACCCGAACCTTCGCAGCCGAAAACGACCTTGGTGTTCCTGTTGCAGCCGTTTACTTCAATGCACAGAGAGAAACGGCAGCAAGAAGACGCTAG

>Line38_TFL1-C_protein_hap1

MARMSEPLAVGRVIGDVLDSFTPTTKMIVTYNTKLVCNGHELFPSAVTAKPRVEIQGGDMRSFFTLVMTDPDVPGPSDPYLKEHL**DCDRHSWHHRCYIW***

>Line38_TFL1-C_DNA_hap2

ATGGCAAGAATGTCGGAACCTCTAGCTGTTGGAAGAGTCATAGGAGATGTTCTTGATTCCTTCACCCCAACTACAAAAATGATTGTCACTTACAACACCAAGCTCGTCTGCAATGGACATGAGCTCTTCCCTTCTGCAGTCACTGCCAAACCGAGAGTTGAGATTCAAGGAGGCGACATGAGATCATTCTTCACTCTGgtatttctttctggcctccttttcggccgtttgttttttttatcagatctaccagatcgctaactttatttattttcacagGTAATGACAGACCCAGATGTTCCTGGCCCTAGTGATCCTTATTTGAAAGAGCACCTG**‑**ACTGgtatatcttacagttaatactgtctatatattatcaaacctagaaacgaagaaacaaagacaataactattcagataattattaataacttcaatgttcaaactagatatatatgttgtcatattctagaaatttgcagtattgaccgaacaaattgtccaggttaatttatagatcaattgcgctcctgcttgtaatttccagattcaaagaaaagattagttattttaattattgttaatttattctttctcctttacatggtcattcatgtattcagaattgtaatccgaaaatctttggatctggaaataaaagcaagtaaaaattacatattatgtcacgaactcttaactagaaaaactagcaagttataattgtggagtatagacatgccaataagcttggatctaacagttttgccttgaaattgaactatacagGATTGTGACAGACATTCCTGGCACCACAGATGCTACATTTGgtaggttaattcacactagttcatgagctaccagtacgttatacagctagtgatgatcagcactgatattgtgtatatactacattaattaacagGAAGAGAAGTGGTGAGCTACGAGATGCCAAGGCCAAACATAGGCATCCACAGGTTTGTGTTTGTTCTCTTCATGCAAAAACGAAGGCAGTCGGTGAACCCGCCTTCCTCAAGGGATCACTTCAACACCCGAACCTTCGCAGCCGAAAACGACCTTGGTGTTCCTGTTGCAGCCGTTTACTTCAATGCACAGAGAGAAACGGCAGCAAGAAGACGCTAG

>Line38_TFL1-C_protein_hap2

MARMSEPLAVGRVIGDVLDSFTPTTKMIVTYNTKLVCNGHELFPSAVTAKPRVEIQGGDMRSFF

TLVMTDPDVPGPSDPYLKEHL**TGL***

>LineXXXX_TFL1-D_DNA

ATGGCAAGAATGTCGGAACCTCTAGCTGTTGGAAGAGTCATAGGAGATGTTCTTGATTCCTTCACCCCAACTACAAAAATGATTGTCACTTACAACACCAAGCTCGTCTGCAATGGACATGAGCTCTTCCCTTCTGCAGTCACCGCCAAACCTAGAGTTGAGATTCAAGGAGGCGACATGAGATCATTCTTCACTCTGgtatacatcattttctttctggcctccttttccgccgtttgttttttttatcagatctaccagatcgctaactttagttattttcacagGTAATGACAGACCCAGATGTTCCTGGCCCTAGTGATCCTTATTTGAAAGAGCACCTGCACTGgtatatcttacagttaatactgtctatatattatcaaacctagaaacgaagaaacaaagacgataactattcagataattattaataacttcaatgttcaaactagatatatatgttgtcatatactagaaatttgcagtattgaccgaacacattgtccaggttaatttatagaaaagattagttatattaattattgttaatttattctttctcctttacatggtcattcacgtatatattcagaattgtaatcagaaaatctttggatctggaaataaaagctagtaaaaattacatattatgtcacgaactcttagttataatggtggagaatatacatgccactaagcttggatctaacagttttgccttgaaattgaactatacagGATTGTGACAGACATTCCTGGCACCACAGATGCTACATTTGgtaggttaattcacactagttcatgagctaccagtacgttatacagctagtgatgatcagcactgatattgtacgtgtatatactacattaattaacagGAAGAGAAGTGGTGAGCTACGAGATGCCAAGGCCAAACATAGGCATCCACAGGTTTGTGTTTGTTCTCTTCATGCAAAAACGAAGGCAGTCGGTGAACCCGCCTTCCTCACGGGATCACTTCAACACCCGAACCTTCGCAGCCGAAAACGACCTTGGTGTTCCTGTTGCTGCCGTTTACTTCAATGCACAGAGAGAAACGGCAGCAAGAAGACGCTAG

>LineXXXX_TFL1-D_protein

MARMSEPLAVGRVIGDVLDSFTPTTKMIVTYNTKLVCNGHELFPSAVTAKPRVEIQGGDMRSFFTLVMTDPDVPGPSDPYLKEHLHWIVTDIPGTTDATFGREVVSYEMPRPNIGIHRFVFVLFMQKRRQSVNPPSSRDHFNTRTFAAENDLGVPVAAVYFNAQRETAARRR*

>LineC20_TFL1-D_DNA_hap1

ATGGCAAGAATGTCGGAACCTCTAGCTGTTGGAAGAGTCATAGGAGATGTTCTTGATTCCTTCACCCCAACTACAAAAATGATTGTCACTTACAACACCAAGCTCGTCTGCAATGGACATGAGCTCTTCCCTTCTGCAGTCACCGCCAAACCTAGAGTTGAGATTCAAGGAGGCGACATGAGATCATTCTTCACTCTGgtatacatcattttctttctggcctccttttccgccgtttgttttttttatcagatctaccagatcgctaactttagttattttcacagGTAATGACAGACCCAGATGTTCCTGGCCCTAGTGATCCTTATTTGAAAGAGC‑‑‑‑‑CACTGgtatatcttacagttaatactgtctatatattatcaaacctagaaacgaagaaacaaagacgataactattcagataattattaataacttcaatgttcaaactagatatatatgttgtcatatactagaaatttgcagtattgaccgaacacattgtccaggttaatttatagaaaagattagttatattaattattgttaatttattctttctcctttacatggtcattcacgtatatattcagaattgtaatcagaaaatctttggatctggaaataaaagctagtaaaaattacatattatgtcacgaactcttagttataatggtggagaatatacatgccactaagcttggatctaacagttttgccttgaaattgaactatacagGATTGTGACAGACATTCCTGGCACCACAGATGCTACATTTGgtaggttaattcacactagttcatgagctaccagtacgttatacagctagtgatgatcagcactgatattgtacgtgtatatactacattaattaacagGAAGAGAAGTGGTGAGCTACGAGATGCCAAGGCCAAACATAGGCATCCACAGGTTTGTGTTTGTTCTCTTCATGCAAAAACGAAGGCAGTCGGTGAACCCGCCTTCCTCACGGGATCACTTCAACACCCGAACCTTCGCAGCCGAAAACGACCTTGGTGTTCCTGTTGCTGCCGTTTACTTCAATGCACAGAGAGAAACGGCAGCAAGAAGACGCTAG

>LineC20_TFL1-D_protein_hap1

MARMSEPLAVGRVIGDVLDSFTPTTKMIVTYNTKLVCNGHELFPSAVTAKPRVEIQGGDMRSFF

TLVMTDPDVPGPSDPYLKE**PLDCDRHSWHHRCYIW***

>LineC20_TFL1-D_DNA_hap2

ATGGCAAGAATGTCGGAACCTCTAGCTGTTGGAAGAGTCATAGGAGATGTTCTTGATTCCTTCACCCCAACTACAAAAATGATTGTCACTTACAACACCAAGCTCGTCTGCAATGGACATGAGCTCTTCCCTTCTGCAGTCACCGCCAAACCTAGAGTTGAGATTCAAGGAGGCGACATGAGATCATTCTTCACTCTGgtatacatcattttctttctggcctccttttccgccgtttgttttttttatcagatctaccagatcgctaactttagttattttcacagGTAATGACAGACCCAGATGTTCCTGGCCCTAGTGATCCTTATTTGAAAGAGCAC**‑‑‑‑**ACTGgtatatcttacagttaatactgtctatatattatcaaacctagaaacgaagaaacaaagacgataactattcagataattattaataacttcaatgttcaaactagatatatatgttgtcatatactagaaatttgcagtattgaccgaacacattgtccaggttaatttatagaaaagattagttatattaattattgttaatttattctttctcctttacatggtcattcacgtatatattcagaattgtaatcagaaaatctttggatctggaaataaaagctagtaaaaattacatattatgtcacgaactcttagttataatggtggagaatatacatgccactaagcttggatctaacagttttgccttgaaattgaactatacagGATTGTGACAGACATTCCTGGCACCACAGATGCTACATTTGgtaggttaattcacactagttcatgagctaccagtacgttatacagctagtgatgatcagcactgatattgtacgtgtatatactacattaattaacagGAAGAGAAGTGGTGAGCTACGAGATGCCAAGGCCAAACATAGGCATCCACAGGTTTGTGTTTGTTCTCTTCATGCAAAAACGAAGGCAGTCGGTGAACCCGCCTTCCTCACGGGATCACTTCAACACCCGAACCTTCGCAGCCGAAAACGACCTTGGTGTTCCTGTTGCTGCCGTTTACTTCAATGCACAGAGAGAAACGGCAGCAAGAAGACGCTAG

>LineC20_TFL1-D_protein_hap2

MARMSEPLAVGRVIGDVLDSFTPTTKMIVTYNTKLVCNGHELFPSAVTAKPRVEIQGGDMRSFF

TLVMTDPDVPGPSDPYLKEH**TGL***

>LineC24_TFL1-D_DNA

ATGGCAAGAATGTCGGAACCTCTAGCTGTTGGAAGAGTCATAGGAGATGTTCTTGATTCCTTCACCCCAACTACAAAAATGATTGTCACTTACAACACCAAGCTCGTCTGCAATGGACATGAGCTCTTCCCTTCTGCAGTCACCGCCAAACCTAGAGTTGAGATTCAAGGAGGCGACATGAGATCATTCTTCACTCTGgtatacatcattttctttctggcctccttttccgccgtttgttttttttatcagatctaccagatcgctaactttagttattttcacagGTAATGACAGACCCAGATGTTCCTGGCCCTAGTGATCCTT**ATTTGAAAGAGCACCTG‑ACTGg**tatatcttacagttaatactgtctatatattatcaaacctagaaacgaagaaacaaagacgataactattcagataattattaataacttcaatgttcaaactagatatatatgttgtcatatactagaaatttgcagtattgaccgaacacattgtccaggttaatttatagaaaagattagttatattaattattgttaatttattctttctcctttacatggtcattcacgtatatattcagaattgtaatcagaaaatctttggatctggaaataaaagctagtaaaaattacatattatgtcacgaactcttagttataatggtggagaatatacatgccactaagcttggatctaacagttttgccttgaaattgaactatacagGATTGTGACAGACATTCCTGGCACCACAGATGCTACATTTGgtaggttaattcacactagttcatgagctaccagtacgttatacagctagtgatgatcagcactgatattgtacgtgtatatactacattaattaacagGAAGAGAAGTGGTGAGCTACGAGATGCCAAGGCCAAACATAGGCATCCACAGGTTTGTGTTTGTTCTCTTCATGCAAAAACGAAGGCAGTCGGTGAACCCGCCTTCCTCACGGGATCACTTCAACACCCGAACCTTCGCAGCCGAAAACGACCTTGGTGTTCCTGTTGCTGCCGTTTACTTCAATGCACAGAGAGAAACGGCAGCAAGAAGACGCTAG

>LineC24_TFL1-D_protein

MARMSEPLAVGRVIGDVLDSFTPTTKMIVTYNTKLVCNGHELFPSAVTAKPRVEIQGGDMRSFFTLVMTDPDVPGPSDPYLKEHL**TGL***

>LineC22_TFL1-D_DNA_hap1

ATGGCAAGAATGTCGGAACCTCTAGCTGTTGGAAGAGTCATAGGAGATGTTCTTGATTCCTTCACCCCAACTACAAAAATGATTGTCACTTACAACACCAAGCTCGTCTGCAATGGACATGAGCTCTTCCCTTCTGCAGTCACCGCCAAACCTAGAGTTGAGATTCAAGGAGGCGA**CATGAGATCATTCTTC‑CTCTGg**tatacatcattttctttctggcctccttttccgccgtttgttttttttatcagatctaccagatcgctaactttagttattttcacagGTAATGACAGACCCAGATGTTCCTGGCCCTAGTGATCCTTATTTGAAAGAGCACCT‑CACTGgtatatcttacagttaatactgtctatatattatcaaacctagaaacgaagaaacaaagacgataactattcagataattattaataacttcaatgttcaaactagatatatatgttgtcatatactagaaatttgcagtattgaccgaacacattgtccaggttaatttatagaaaagattagttatattaattattgttaatttattctttctcctttacatggtcattcacgtatatattcagaattgtaatcagaaaatctttggatctggaaataaaagctagtaaaaattacatattatgtcacgaactcttagttataatggtggagaatatacatgccactaagcttggatctaacagttttgccttgaaattgaactatacagGATTGTGACAGACATTCCTGGCACCACAGATGCTACATTTGgtaggttaattcacactagttcatgagctaccagtacgttatacagctagtgatgatcagcactgatattgtacgtgtatatactacattaattaacagGAAGAGAAGTGGTGAGCTACGAGATGCCAAGGCCAAACATAGGCATCCACAGGTTTGTGTTTGTTCTCTTCATGCAAAAACGAAGGCAGTCGGTGAACCCGCCTTCCTCACGGGATCACTTCAACACCCGAACCTTCGCAGCCGAAAACGACCTTGGTGTTCCTGTTGCTGCCGTTTACTTCAATGCACAGAGAGAAACGGCAGCAAGAAGACGCTAG

>LineC22_TFL1-D_protein_hap1

MARMSEPLAVGRVIGDVLDSFTPTTKMIVTYNTKLVCNGHELFPSAVTAKPRVEIQGGDMRSFF**LW***

>LineC22_TFL1-D_DNA_hap2

ATGGCAAGAATGTCGGAACCTCTAGCTGTTGGAAGAGTCATAGGAGATGTTCTTGATTCCTTCACCCCAACTACAAAAATGATTGTCACTTACAACACCAAGCTCGTCTGCAATGGACATGAGCTCTTCCCTTCTGCAGTCACCGCCAAACCTAGAGTTGAGATTCAAGGAGGCGACATGAGATCATTCTTCACTCTGgtatacatcattttctttctggcctccttttccgccgtttgttttttttatcagatctaccagatcgctaactttagttattttcacagGTAATGACAGACCCAGATGTTCCTGGCCCTAGTGATCCTTATTTGAAAGAGCACCTG**‑**ACTGgtatatcttacagttaatactgtctatatattatcaaacctagaaacgaagaaacaaagacgataactattcagataattattaataacttcaatgttcaaactagatatatatgttgtcatatactagaaatttgcagtattgaccgaacacattgtccaggttaatttatagaaaagattagttatattaattattgttaatttattctttctcctttacatggtcattcacgtatatattcagaattgtaatcagaaaatctttggatctggaaataaaagctagtaaaaattacatattatgtcacgaactcttagttataatggtggagaatatacatgccactaagcttggatctaacagttttgccttgaaattgaactatacagGATTGTGACAGACATTCCTGGCACCACAGATGCTACATTTGgtaggttaattcacactagttcatgagctaccagtacgttatacagctagtgatgatcagcactgatattgtacgtgtatatactacattaattaacagGAAGAGAAGTGGTGAGCTACGAGATGCCAAGGCCAAACATAGGCATCCACAGGTTTGTGTTTGTTCTCTTCATGCAAAAACGAAGGCAGTCGGTGAACCCGCCTTCCTCACGGGATCACTTCAACACCCGAACCTTCGCAGCCGAAAACGACCTTGGTGTTCCTGTTGCTGCCGTTTACTTCAATGCACAGAGAGAAACGGCAGCAAGAAGACGCTAG

>LineC22_TFL1-D_protein_hap2

MARMSEPLAVGRVIGDVLDSFTPTTKMIVTYNTKLVCNGHELFPSAVTAKPRVEIQGGDMRSFF

TLVMTDPDVPGPSDPYLKEHL**TGL***

>LineC21_TFL1-D_DNA_hap1

ATGGCAAGAATGTCGGAACCTCTAGCTGTTGGAAGAGTCATAGGAGATGTTCTTGATTCCTTCACCCCAACTACAAAAATGATTGTCACTTACAACACCAAGCTCGTCTGCAATGGACATGAGCTCTTCCCTTCTGCAGTCACCGCCAAACCTAGAGTTGAGATTCAAGGAGGCGACATGAGATCATTCTTCACTCTGgtatacatcattttctttctggcctccttttccgccgtttgttttttttatcagatctaccagatcgctaactttagttattttcacagGTAATGACAGACCCAGATGTTCCTGGCCCTAGTGATCCTT**ATTTGAAAGAGCACCTG‑‑‑‑‑g**tatatcttacagttaatactgtctatatattatcaaacctagaaacgaagaaacaaagacgataactattcagataattattaataacttcaatgttcaaactagatatatatgttgtcatatactagaaatttgcagtattgaccgaacacattgtccaggttaatttatagaaaagattagttatattaattattgttaatttattctttctcctttacatggtcattcacgtatatattcagaattgtaatcagaaaatctttggatctggaaataaaagctagtaaaaattacatattatgtcacgaactcttagttataatggtggagaatatacatgccactaagcttggatctaacagttttgccttgaaattgaactatacagGATTGTGACAGACATTCCTGGCACCACAGATGCTACATTTGgtaggttaattcacactagttcatgagctaccagtacgttatacagctagtgatgatcagcactgatattgtacgtgtatatactacattaattaacagGAAGAGAAGTGGTGAGCTACGAGATGCCAAGGCCAAACATAGGCATCCACAGGTTTGTGTTTGTTCTCTTCATGCAAAAACGAAGGCAGTCGGTGAACCCGCCTTCCTCACGGGATCACTTCAACACCCGAACCTTCGCAGCCGAAAACGACCTTGGTGTTCCTGTTGCTGCCGTTTACTTCAATGCACAGAGAGAAACGGCAGCAAGAAGACGCTAG

>LineC21_TFL1-D_protein_hap1

MARMSEPLAVGRVIGDVLDSFTPTTKMIVTYNTKLVCNGHELFPSAVTAKPRVEIQGGDMRSFFTLVMTDPDVPGPSDPYLKEHL**DCDRHSWHHRCYIW***

>LineC21_TFL1-D_DNA_hap2

ATGGCAAGAATGTCGGAACCTCTAGCTGTTGGAAGAGTCATAGGAGATGTTCTTGATTCCTTCACCCCAACTACAAAAATGATTGTCACTTACAACACCAAGCTCGTCTGCAATGGACATGAGCTCTTCCCTTCTGCAGTCACCGCCAAACCTAGAGTTGAGATTCAAGGAGGCGACATGAGATCATTCTTCACTCTGgtatacatcattttctttctggcctccttttccgccgtttgttttttttatcagatctaccagatcgctaactttagttattttcacagGTAATGACAGACCCAGATGTTCCTGGCCCTAGTGATCCTTATTTGAAAGAGCACCTG**T**CACTGgtatatcttacagttaatactgtctatatattatcaaacctagaaacgaagaaacaaagacgataactattcagataattattaataacttcaatgttcaaactagatatatatgttgtcatatactagaaatttgcagtattgaccgaacacattgtccaggttaatttatagaaaagattagttatattaattattgttaatttattctttctcctttacatggtcattcacgtatatattcagaattgtaatcagaaaatctttggatctggaaataaaagctagtaaaaattacatattatgtcacgaactcttagttataatggtggagaatatacatgccactaagcttggatctaacagttttgccttgaaattgaactatacagGATTGTGACAGACATTCCTGGCACCACAGATGCTACATTTGgtaggttaattcacactagttcatgagctaccagtacgttatacagctagtgatgatcagcactgatattgtacgtgtatatactacattaattaacagGAAGAGAAGTGGTGAGCTACGAGATGCCAAGGCCAAACATAGGCATCCACAGGTTTGTGTTTGTTCTCTTCATGCAAAAACGAAGGCAGTCGGTGAACCCGCCTTCCTCACGGGATCACTTCAACACCCGAACCTTCGCAGCCGAAAACGACCTTGGTGTTCCTGTTGCTGCCGTTTACTTCAATGCACAGAGAGAAACGGCAGCAAGAAGACGCTAG

>LineC21_TFL1-D_protein_hap2

MARMSEPLAVGRVIGDVLDSFTPTTKMIVTYNTKLVCNGHELFPSAVTAKPRVEIQGGDMRSFF

TLVMTDPDVPGPSDPYLKEHL**SLDCDRHSWHHRCYIW***

>LineC19_TFL1-D_DNA

ATGGCAAGAATGTCGGAACCTCTAGCTGTTGGAAGAGTCATAGGAGATGTTCTTGATTCCTTCACCCCAACTACAAAAATGATTGTCACTTACAACACCAAGCTCGTCTGCAATGGACATGAGCTCTTCCCTTCTGCAGTCACCGCCAAACCTAGAGTTGAGATTCAAGGAGGCGACATGAGATCATTCTTCACTCTGgtatacatcattttctttctggcctccttttccgccgtttgttttttttatcagatctaccagatcgctaactttagttattttcacagGTAATGACAGACCCAGATGTTCCTGGCCCTAGTGATCCTT**ATTTGAAAGAGCACCTG‑ACTGg**tatatcttacagttaatactgtctatatattatcaaacctagaaacgaagaaacaaagacgataactattcagataattattaataacttcaatgttcaaactagatatatatgttgtcatatactagaaatttgcagtattgaccgaacacattgtccaggttaatttatagaaaagattagttatattaattattgttaatttattctttctcctttacatggtcattcacgtatatattcagaattgtaatcagaaaatctttggatctggaaataaaagctagtaaaaattacatattatgtcacgaactcttagttataatggtggagaatatacatgccactaagcttggatctaacagttttgccttgaaattgaactatacagGATTGTGACAGACATTCCTGGCACCACAGATGCTACATTTGgtaggttaattcacactagttcatgagctaccagtacgttatacagctagtgatgatcagcactgatattgtacgtgtatatactacattaattaacagGAAGAGAAGTGGTGAGCTACGAGATGCCAAGGCCAAACATAGGCATCCACAGGTTTGTGTTTGTTCTCTTCATGCAAAAACGAAGGCAGTCGGTGAACCCGCCTTCCTCACGGGATCACTTCAACACCCGAACCTTCGCAGCCGAAAACGACCTTGGTGTTCCTGTTGCTGCCGTTTACTTCAATGCACAGAGAGAAACGGCAGCAAGAAGACGCTAG

>LineC19_TFL1-D_protein

MARMSEPLAVGRVIGDVLDSFTPTTKMIVTYNTKLVCNGHELFPSAVTAKPRVEIQGGDMRSFFTLVMTDPDVPGPSDPYLKEHL**TGL***

>Line14_TFL1-D_DNA_hap1

ATGGCAAGAATGTCGGAACCTCTAGCTGTTGGAAGAGTCATAGGAGATGTTCTTGATTCCTTCACCCCAACTACAAAAATGATTGTCACTTACAACACCAAGCTCGTCTGCAATGGACATGAGCTCTTCCCTTCTGCAGTCACCGCCAAACCTAGAGTTGAGATTCAAGGAGGCGACATGAGATCATTCTTCACTCTGgtatacatcattttctttctggcctccttttccgccgtttgttttttttatcagatctaccagatcgctaactttagttattttcacagGTAATGACAGACCCAGATGTTCCTGGCCCTAGTGATCCTTATTTGAAAGAGCACCTGGCACTGgtatatcttacagttaatactgtctatatattatcaaacctagaaacgaagaaacaaagacgataactattcagataattattaataacttcaatgttcaaactagatatatatgttgtcatatactagaaatttgcagtattgaccgaacacattgtccaggttaatttatagaaaagattagttatattaattattgttaatttattctttctcctttacatggtcattcacgtatatattcagaattgtaatcagaaaatctttggatctggaaataaaagctagtaaaaattacatattatgtcacgaactcttagttataatggtggagaatatacatgccactaagcttggatctaacagttttgccttgaaattgaactatacagGATTGTGACAGACATTCCTGGCACCACAGATGCTACATTTGgtaggttaattcacactagttcatgagctaccagtacgttatacagctagtgatgatcagcactgatattgtacgtgtatatactacattaattaacagGAAGAGAAGTGGTGAGCTACGAGATGCCAAGGCCAAACATAGGCATCCACAGGTTTGTGTTTGTTCTCTTCATGCAAAAACGAAGGCAGTCGGTGAACCCGCCTTCCTCACGGGATCACTTCAACACCCGAACCTTCGCAGCCGAAAACGACCTTGGTGTTCCTGTTGCTGCCGTTTACTTCAATGCACAGAGAGAAACGGCAGCAAGAAGACGCTAG

>Line14_TFL1-D_protein_hap1

MARMSEPLAVGRVIGDVLDSFTPTTKMIVTYNTKLVCNGHELFPSAVTAKPRVEIQGGDMRSFFTLVMTDPDVPGPSDPYLKEHL**ALDCDRHSWHHRCYIW***

>Line14_TFL1-D_DNA_hap2

ATGGCAAGAATGTCGGAACCTCTAGCTGTTGGAAGAGTCATAGGAGATGTTCTTGATTCCTTCACCCCAACTACAAAAATGATTGTCACTTACAACACCAAGCTCGTCTGCAATGGACATGAGCTCTTCCCTTCTGCAGTCACCGCCAAACCTAGAGTTGAGATTCAAGGAGGCGACATGAGATCATTCTTCACTCTGgtatacatcattttctttctggcctccttttccgccgtttgttttttttatcagatctaccagatcgctaactttagttattttcacagGTAATGACAGACCCAGATGTTCCTGGCCCTAGTGATCCTTATTTGAAAGAGCACC‑‑‑ACTGgtatatcttacagttaatactgtctatatattatcaaacctagaaacgaagaaacaaagacgataactattcagataattattaataacttcaatgttcaaactagatatatatgttgtcatatactagaaatttgcagtattgaccgaacacattgtccaggttaatttatagaaaagattagttatattaattattgttaatttattctttctcctttacatggtcattcacgtatatattcagaattgtaatcagaaaatctttggatctggaaataaaagctagtaaaaattacatattatgtcacgaactcttagttataatggtggagaatatacatgccactaagcttggatctaacagttttgccttgaaattgaactatacagGATTGTGACAGACATTCCTGGCACCACAGATGCTACATTTGgtaggttaattcacactagttcatgagctaccagtacgttatacagctagtgatgatcagcactgatattgtacgtgtatatactacattaattaacagGAAGAGAAGTGGTGAGCTACGAGATGCCAAGGCCAAACATAGGCATCCACAGGTTTGTGTTTGTTCTCTTCATGCAAAAACGAAGGCAGTCGGTGAACCCGCCTTCCTCACGGGATCACTTCAACACCCGAACCTTCGCAGCCGAAAACGACCTTGGTGTTCCTGTTGCTGCCGTTTACTTCAATGCACAGAGAGAAACGGCAGCAAGAAGACGCTAG

>Line14_TFL1-D_protein_hap2

MARMSEPLAVGRVIGDVLDSFTPTTKMIVTYNTKLVCNGHELFPSAVTAKPRVEIQGGDMRSFFTLVMTDPDVPGPSDPYLKEH**‑**HWIVTDIPGTTDATFGREVVSYEMPRPNIGIHRFVFVLFMQKRRQSVNPPSSRDHFNTRTFAAENDLGVPVAAVYFNAQRETAARRR*

>Line15_TFL1-D_DNA

‑‑‑‑‑‑‑‑‑‑‑‑‑‑ttagttattttcacagGTAATGACAGACCCAGATGTTCCTG‑‑‑‑‑‑‑‑‑‑‑‑‑‑‑‑‑‑‑‑‑‑‑‑‑‑‑‑‑‑‑GCACTGgtatatcttacagttaatactgtctatatattatcaaacctagaaacgaagaaacaaagacgataactattcagataattattaataacttcaatgttcaaactagatatatatgttgtcatatactagaaatttgcagtattgaccgaacacattgtccaggttaatttatagaaaagattagttatattaattattgttaatttattctttctcctttacatggtcattcacgtatatattcagaattgtaatcagaaaatctttggatctggaaataaaagctagtaaaaattacatattatgtcacgaactcttagttataatggtggagaatatacatgccactaagcttggatctaacagttttgccttgaaattgaactatacagGATTGTGACAGACATTCCTGGCACCACAGATGCTACATTTGgtaggttaattcacactagttcatgagctaccagtacgttatacagctagtgatgatcagcactgatattgtacgtgtatatactacattaattaacagGAAGAGAAGTGGTGAGCTACGAGATGCCAAGGCCAAACATAGGCATCCACAGGTTTGTGTTTGTTCTCTTCATGCAAAAACGAAGGCAGTCGGTGAACCCGCCTTCCTCACGGGATCACTTCAACACCCGAACCTTCGCAGCCGAAAACGACCTTGGTGTTCCTGTTGCTGCCGTTTACTTCAATGCACAGAGAGAAACGGCAGCAAGAAGACGCTAG

>Line15_TFL1-D_protein

*

>Line38_TFL1-D_DNA

ATGGCAAGAATGTCGGAACCTCTAGCTGTTGGAAGAGTCATAGGAGATGTTCTTGATTCCTTCACCCCAACTACAAAAATGATTGTCACTTACAACACCAAGCTCGTCTGCAATGGACATGAGCTCTTCCCTTCTGCAGTCACCGCCAAACCTAGAGTTGAGATTCAAGGAGGCGACATGAGATCATT‑‑‑‑‑‑‑‑‑‑‑‑‑‑‑‑‑‑‑‑‑‑‑‑‑‑‑‑‑‑‑‑‑‑‑‑‑‑‑‑‑‑‑‑‑‑‑‑‑‑‑‑‑‑‑‑‑‑‑‑‑‑‑‑‑‑‑‑‑‑‑‑‑‑‑‑‑‑‑‑‑‑‑‑‑‑‑‑‑‑‑‑‑‑‑‑‑‑‑‑‑‑‑‑‑‑‑‑‑‑‑‑‑‑‑‑‑‑‑‑‑‑‑‑‑‑‑‑‑‑‑‑‑‑‑‑‑‑‑‑‑‑‑‑‑‑‑‑‑‑‑‑‑‑‑CACTGgtatatcttacagttaatactgtctatatattatcaaacctagaaacgaagaaacaaagacgataactattcagataattattaataacttcaatgttcaaactagatatatatgttgtcatatactagaaatttgcagtattgaccgaacacattgtccaggttaatttatagaaaagattagttatattaattattgttaatttattctttctcctttacatggtcattcacgtatatattcagaattgtaatcagaaaatctttggatctggaaataaaagctagtaaaaattacatattatgtcacgaactcttagttataatggtggagaatatacatgccactaagcttggatctaacagttttgccttgaaattgaactatacagGATTGTGACAGACATTCCTGGCACCACAGATGCTACATTTGgtaggttaattcacactagttcatgagctaccagtacgttatacagctagtgatgatcagcactgatattgtacgtgtatatactacattaattaacagGAAGAGAAGTGGTGAGCTACGAGATGCCAAGGCCAAACATAGGCATCCACAGGTTTGTGTTTGTTCTCTTCATGCAAAAACGAAGGCAGTCGGTGAACCCGCCTTCCTCACGGGATCACTTCAACACCCGAACCTTCGCAGCCGAAAACGACCTTGGTGTTCCTGTTGCTGCCGTTTACTTCAATGCACAGAGAGAAACGGCAGCAAGAAGACGCTAG

>Line38_TFL1-D_protein

MARMSEPLAVGRVIGDVLDSFTPTTKMIVTYNTKLVCNGHELFPSAVTAKPRVEIQGGDMRSF**TGL***
