# Supplementary material for: CRISPR editing of TERMINAL FLOWER 1 recapitulates perpetual flowering in strawberry, mimicking repression by the PERPETUAL FLOWERING locus
Source: Plant Physiol. 2026 Oct 9;202(2):kiag684. doi: 10.1093/plphys/kiag684 (PMC13650783; doi:10.1093/plphys/kiag684)
Supplement: Supplementary file 1 [file kiag684_supplementary_data.zip › SupplementalFigures.pdf]

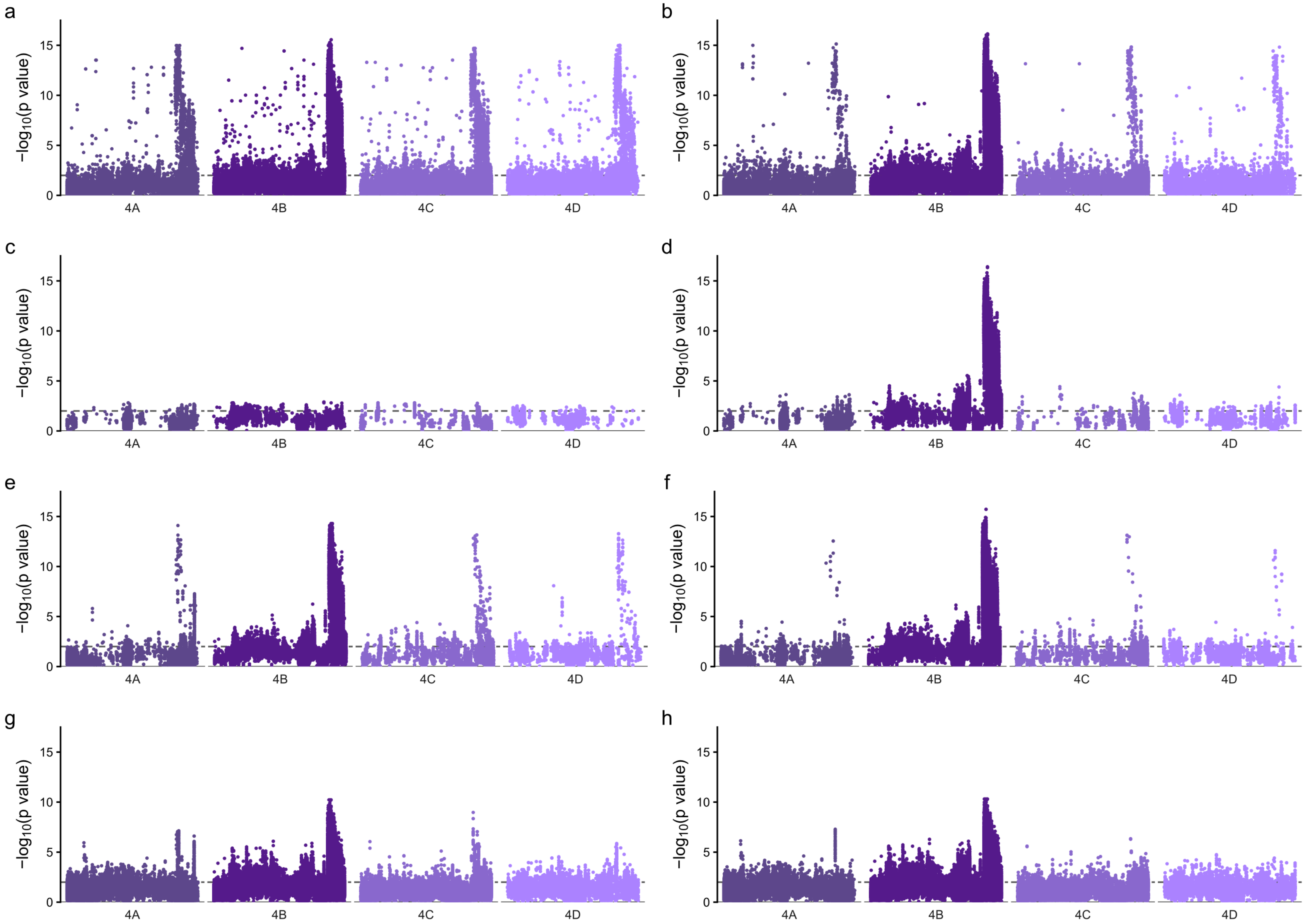

Figure S1. 101-mers provide subgenome specificity. 31-mers filtered to mapping quality greater than 10 map across all homoeologous chromosome 4 copies in each subgenome on both FaRR1-SF (a) and FaRR1-PF (b). Increasing mapping quality to greater than 40 removes off-target mapping on FaRR1-PF (d) but removes all significant mapping on FaRR1-SF (c). Increasing k-mer size to 50, with mapping quality greater than 40 improves specificity on both haplotypes (e,f), while 101-mers provide the greatest specificity, particularly on FaRR1-PF (g,h). For all graphs, p values are adjusted with FDR correction.

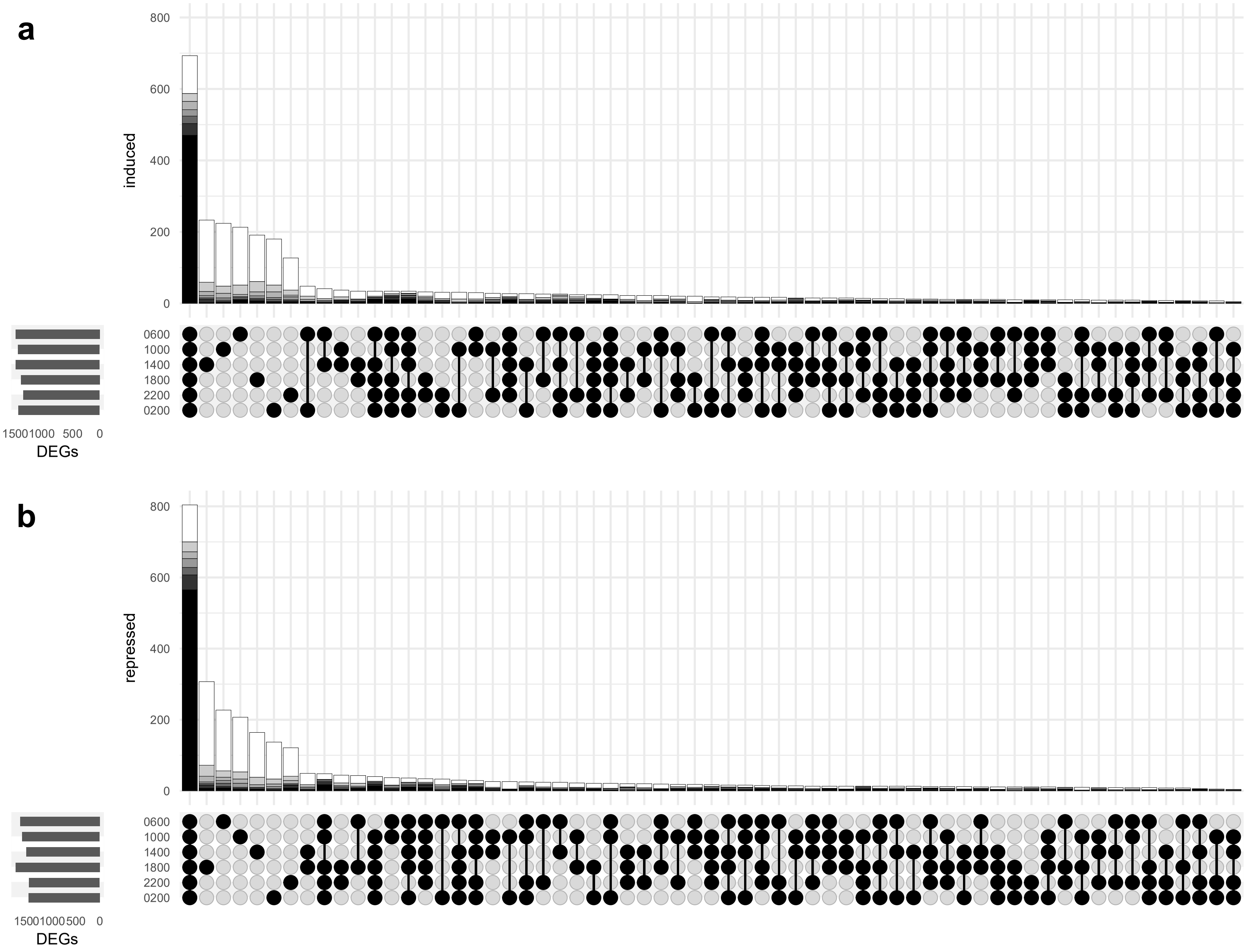

Figure S2. The largest category of DEGs between 'Mojo' and 'Victor' is the set differentially regulated in both tissues at all times. Upset (Lex et al., 2014) diagrams of DEGs induced (a,c) or repressed (b,d) in 'Mojo' relative to 'Victor', in leaves (a,b) or crowns (c,d). In each plot, the intersections of timepoints at which a gene was found differentially expressed is presented, with the bar height showing the number of genes in that intersection. The intensity of color represents the number of times those genes were found differentially expressed in the reciprocal tissue (white = none, black = all six time points).

c

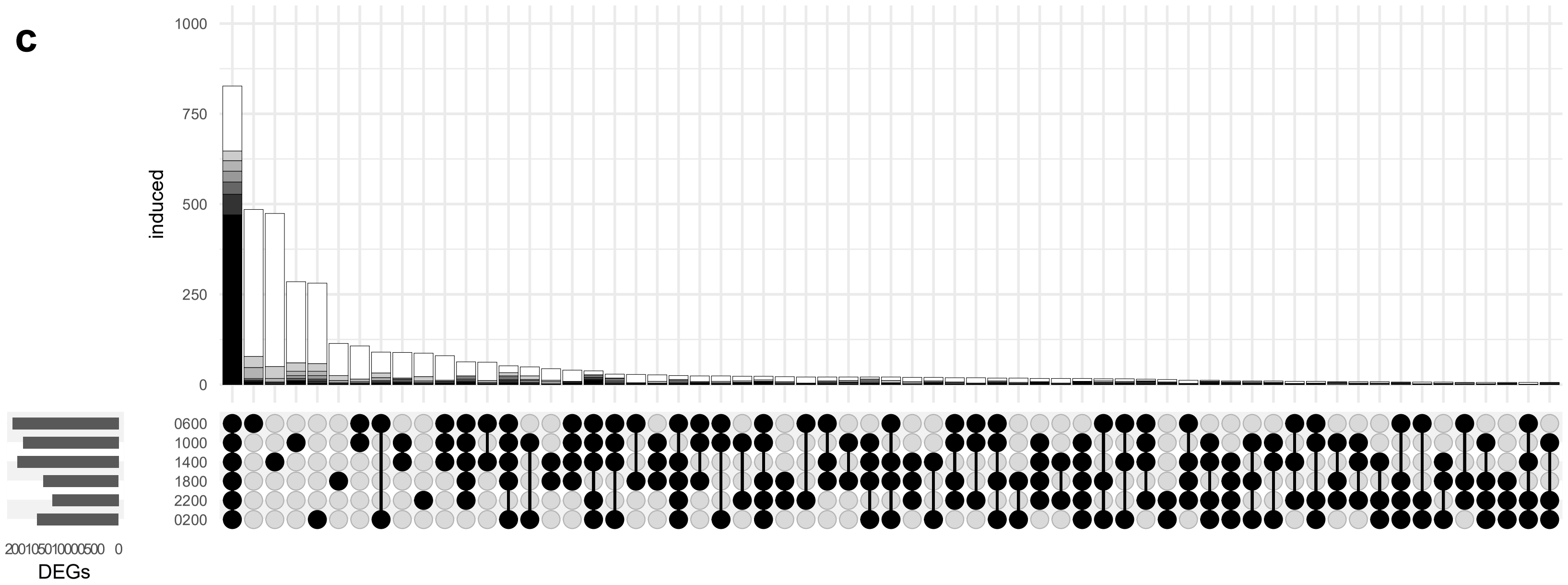

1000
750
500
250
0
induced
0600
1000
1400
1800
2200
0200
DEGs

d

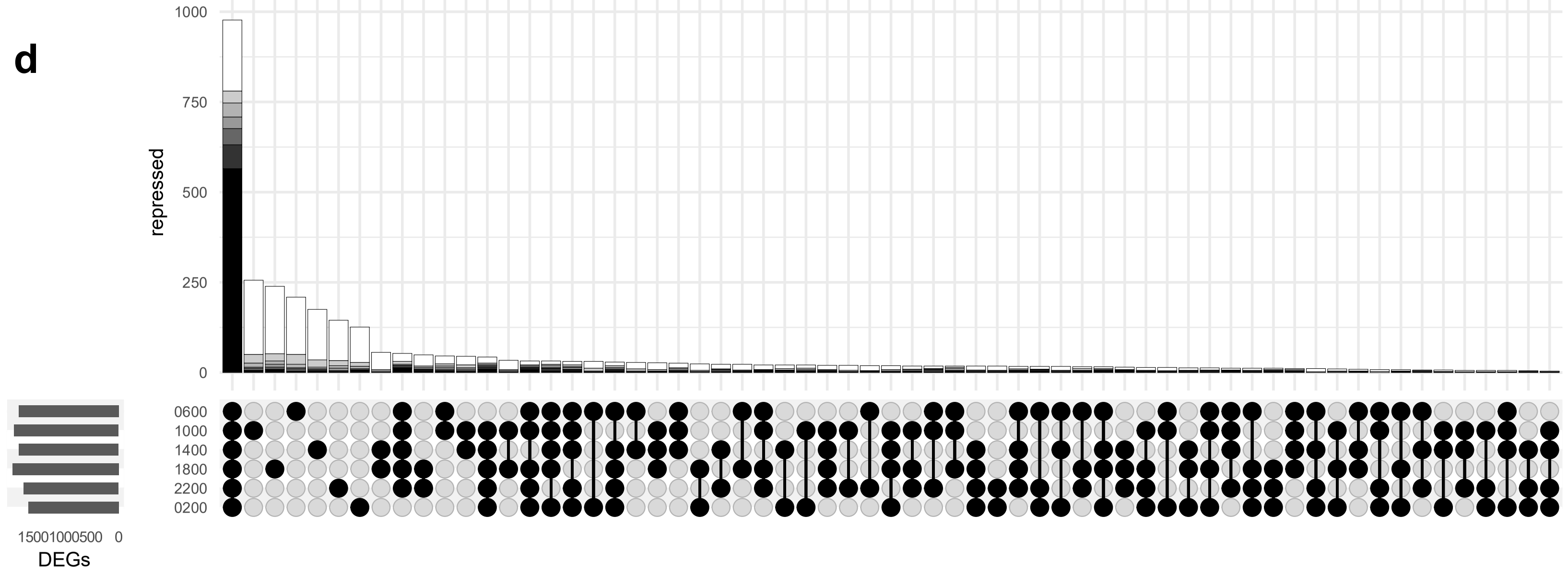

1000
750
500
250
0
repressed
0600
1000
1400
1800
2200
0200
1500
1000
500
0
DEGs

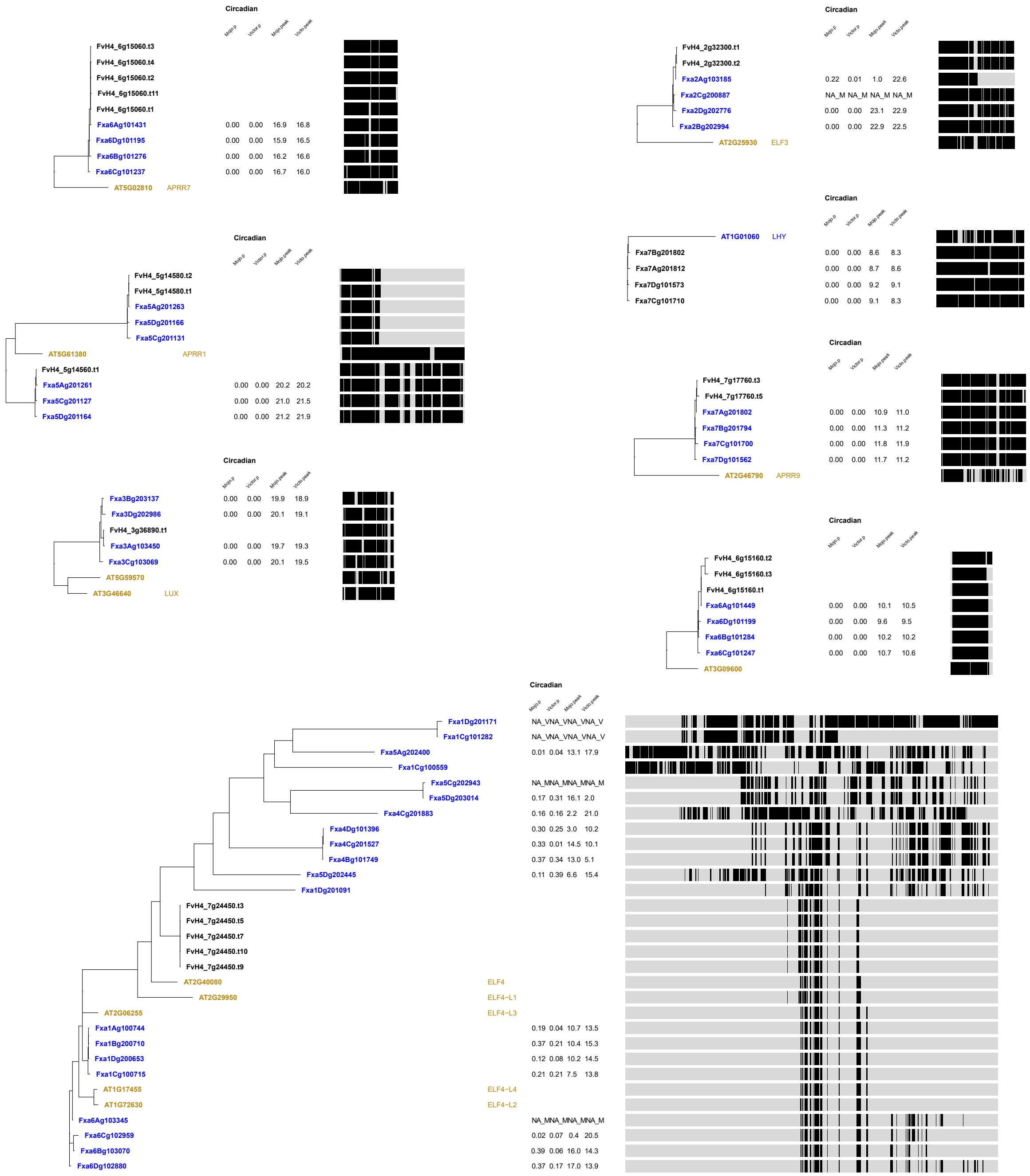

Figure S3. BLAST-align-tree results identifying circadian clock gene homologs in *F.* × *ananassa*. From left-to-right for each gene set: homologs of *A. thaliana* proteins are identified in *F. vesca* and *F.* × *ananassa* by protein sequence similarity and presented in a gene tree. The p-value for rhythmicity and time of peak expression of each *F.* × *ananassa* gene, as calculated by circacompare in this paper's RNAseq experiment, is represented for each tissue and accession. A simplified multiple sequence alignment cartoon, with protein sequence in black and gaps in grey, highlights potentially truncated or domain-altered homologs.

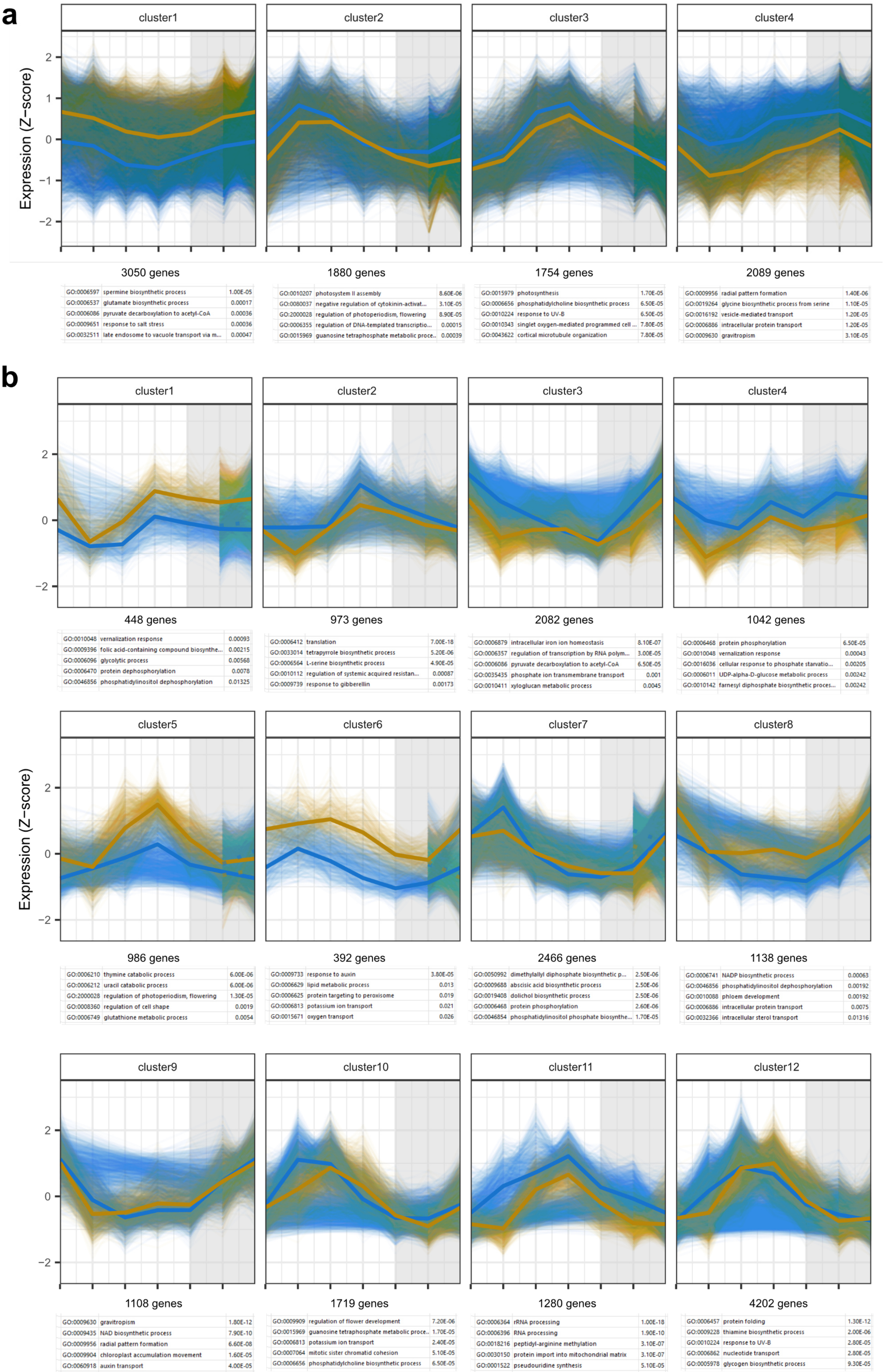

Figure S4. Gene expression clusters of rhythmic genes in crowns (a) and leaves (b), with number of genes per cluster and top 5 enriched GO terms for each cluster. Data in panel a are also shown in Figure 2, here with additional information on cluster size and enriched GO terms.

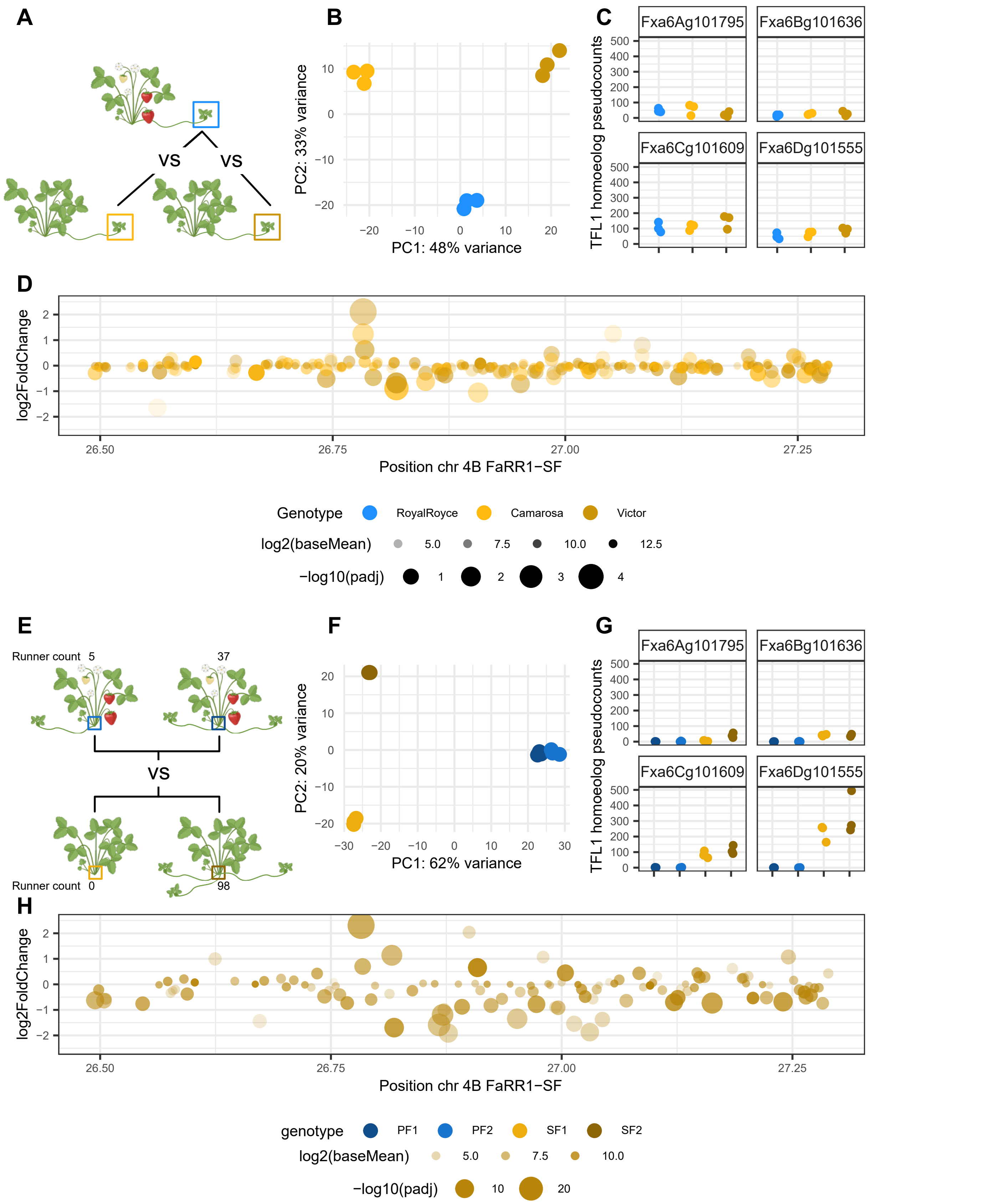

Figure S5. *TFL1* is consistently induced in mature SF crowns. In transcripts of runner tips (immature crown tissue) collected in the open field in July (a), SF and PF individuals are well-separated (b) and differential gene expression is observed in 'Royal Royce' relative to 'Camarosa' (light yellow) and 'Victor' (dark yellow, d), but *TFL1* is expressed at moderate levels in all accessions (c). In transcripts of two sets of siblings, each pair *PFPF* vs *pfpf* - *PFPF* siblings derived from 'Royal Royce' crossed with an unnamed breeding line, and *pfpf* siblings derived from 'UCD Keystone' by an unnamed breeding line - (e) *PFPF* individuals are well-separated from *pfpf* individuals (f), with extensive differential expression in the PF region (h) and high *TFL1* expression specifically in *pfpf* individuals (g). In panels D and H, differential expression of genes in the PF region are shown. Each gene is placed at it's location in the genome (x axis), with position on the y-axis determined by the L2FC of the specified contrast (Panel D, Royal Royce vs. Camarosa, pale yellow; or Royal Royce vs Victor, dark yellow. Panel H both PF siblings vs both SF siblings). The opacity of each point represents the base expression, and the size the FDR-adjusted p value of the contrast. Thus, strongly and reliably differentially expressed genes would be large, opaque points well separated from the x axis. In panel D, opacity and size are shown in greyscale to apply to both Camarosa and Victor contrasts.

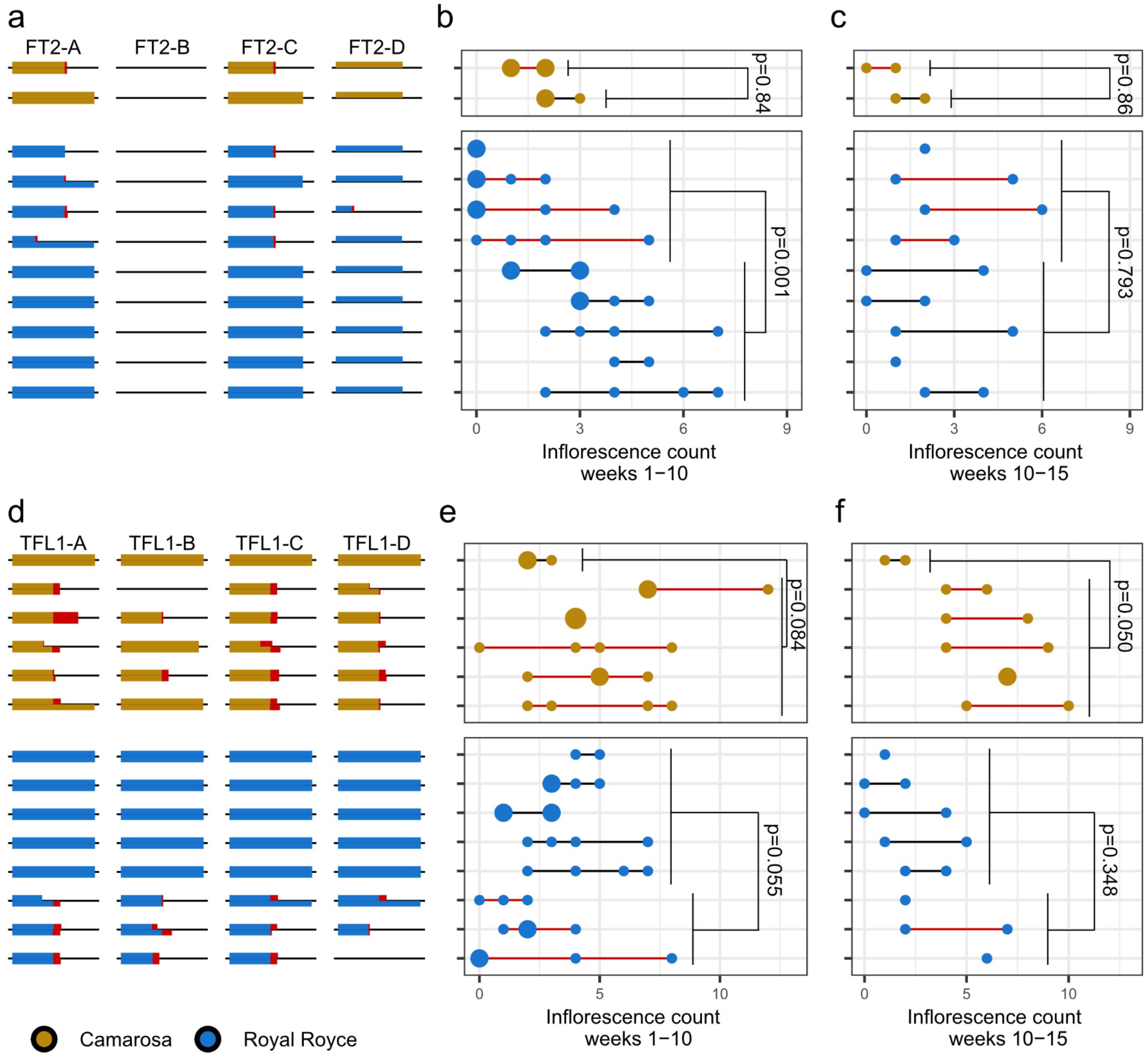

Figure S6. Complete CRISPR information. (a,d) Diagram of FT2 (a) or TFL1 (d) protein from the A, B, C, and D subgenomes in each assayed genotype (row) of Camarosa (*pfpf*, yellow) or Royal Royce (*PFpf*, blue). Yellow or blue indicates parental sequence and red indicates mis-sense translation; a shorter box indicates a premature stop and smaller protein product. Where a plant is heterozygous or bi-allelic, the two products are represented above and below the horizontal black line. (b,e) Inflorescences produced during weeks 1-10 of assay, expanding from floral meristems differentiated during cool short-day pre-experimental conditions. (c,f) Inflorescences produced during weeks 10-15 of assay, expanding from floral meristems differentiated during warm long-day experimental conditions. Significant differences from un-edited control (ANOVA followed by Dunnett's post-hoc comparison) are highlighted. Data is partially overlapping with Figure 5; in this figure edits of *FT2* in Camarosa and *TFL1* in Royal Royce are shown. Unedited plants for each genotype are the same in panels d-f as a-c.

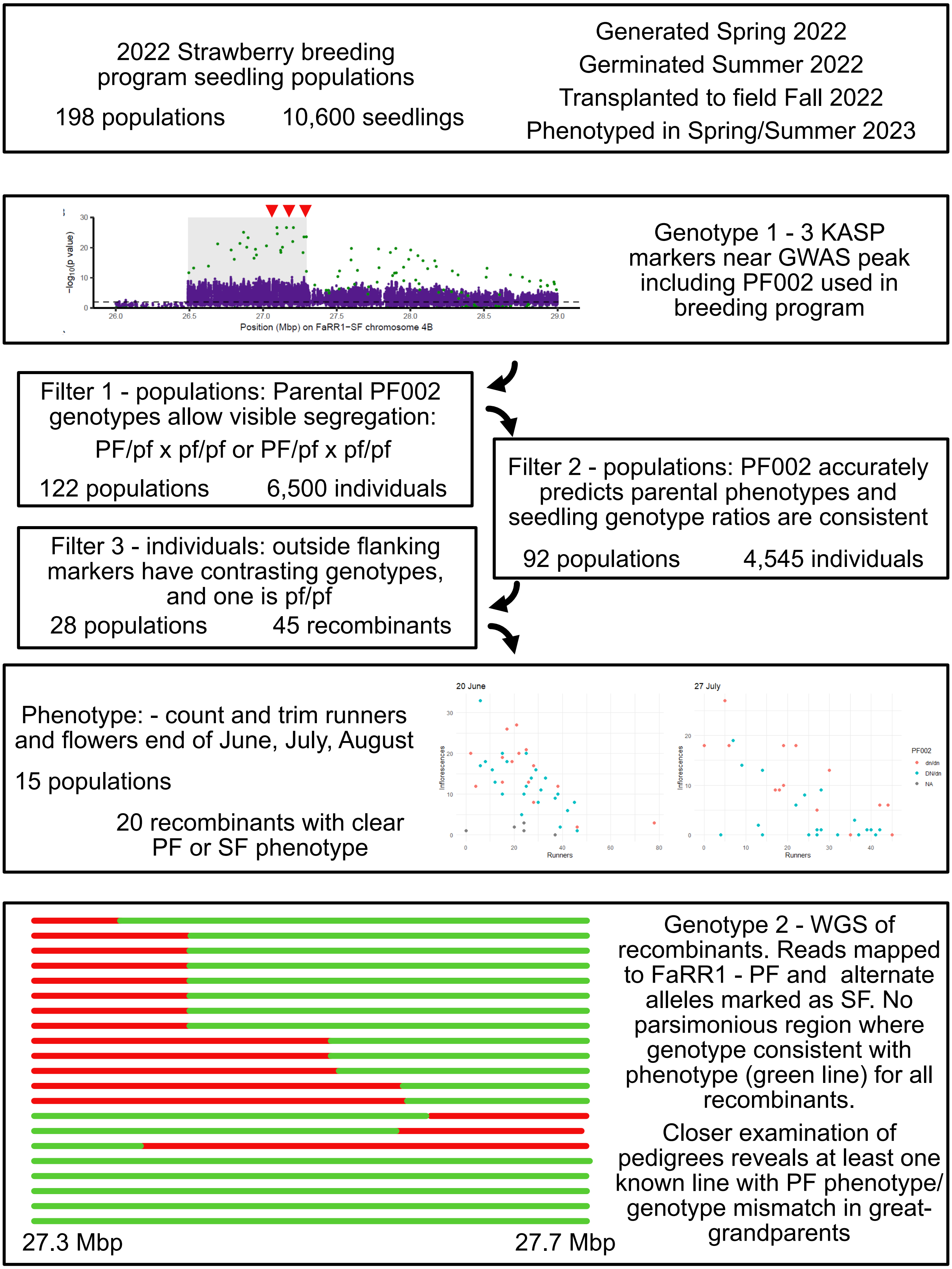

Figure S7. Overview of breeding program recombinants analysis. From top to bottom, boxes outline (i) the population size and timeline of the experiment (ii) the approximate locations of 3 markers used to search for recombinants (iii-v) the filters used to identify recombinants (vi) the phenotyping strategy and results for identified recombinants (vii) the NGS genotyping results.
